# *Bifidobacterium thermophilum* RBL67 impacts on growth and virulence gene expression of *Salmonella enterica* subsp. *enterica* serovar Typhimurium

**DOI:** 10.1186/s12866-016-0659-x

**Published:** 2016-03-18

**Authors:** Sabine A. Tanner, Christophe Chassard, Eugenia Rigozzi, Christophe Lacroix, Marc J. A. Stevens

**Affiliations:** Laboratory of Food Biotechnology, ETH Zurich, Institute of Food, Nutrition and Health, Schmelzbergstrasse 7, Zurich, Switzerland; Present Address: Institut National de la Recherche Agronomique, UR 545 URF, 15000 Aurillac, France

**Keywords:** *Bifidobacterium thermophilum*, *Salmonella* Typhimurium, Co-culture, RNA-seq, Probiotic, Anti-microbial, Virulence

## Abstract

**Background:**

*Bifidobacterium thermophilum* RBL67 (RBL67), a human fecal isolate and health promoting candidate shows antagonistic and protective effects against *Salmonella* and *Listeria* spec. in vitro. However, the underlying mechanisms fostering these effects remain unknown. In this study, the interactions of RBL67 and *Salmonella enterica* subsp. *enterica* serovar Typhimurium N-15 (N-15) were explored by global transcriptional analysis.

**Results:**

Growth experiments were performed in a complex nutritive medium with controlled pH of 6.0 and suitable for balanced growth of both RBL67 and N-15. RBL67 growth was slightly enhanced in presence of N-15. Conversely, N-15 showed reduced growth in the presence of RBL67. Transcriptional analyses revealed higher expression of stress genes and amino acid related function in RBL67 in co-culture with N-15 when compared to mono-culture. Repression of the PhoP regulator was observed in N-15 in presence of RBL67. Further, RBL67 activated virulence genes located on the *Salmonella* pathogenicity islands 1 and 2. Flagellar genes, however, were repressed by RBL67. Sequential expression of flagellar, SPI 1 and fimbrial genes is essential for *Salmonella* infection. Our data revealed that RBL67 triggers expression of SPI 1 and fimbrial determinants prematurely, potentially leading to redundant energy expenditure. In the competitive environment of the gut such energy expenditure could lead to enhanced clearing of *Salmonella*.

**Conclusion:**

Our study provides first insights into probiotic-pathogen interactions on global transcriptional level and suggests that deregulation of virulence gene expression might be an additional protective mechanism of probiotica against infections of the host.

**Electronic supplementary material:**

The online version of this article (doi:10.1186/s12866-016-0659-x) contains supplementary material, which is available to authorized users.

## Background

Probiotics are live organism that, when administered in adequate amounts, confer a health benefit on the host [1]. They exert their beneficial effect via a wide array of mechanisms including direct and indirect antagonism with enteropathogens, improvement of the intestinal barrier function and activation of the mucosal immune system [2, 3]. Direct antagonism with enteropathogens is mediated via production of antimicrobial compounds such as organic acids and bacteriocins, competition for nutrients and minerals, and occupation of adhesion sites [2]. Bifidobacteria and lactobacilli are important constituents of the human gut microbiota and have been associated with a good health status of the host [3, 4]. They are the two major genera used for probiotic applications and have a long history of safe use. Specific strains from bifidobacteria and lactobacilli have been shown to protect against pathogens, with strain specific effects [4].

*Bifidobacterium thermophilum* is a relatively oxygen tolerant *Bifidobacterium* species that has been isolated from bovine rumen, sewage, and from piglet, calf and baby feces [5, 6]. Peptidoglycans from *B. thermophilum* strain P2-91 protect mice against *Escherichia coli* infections and improve cytotoxic activity of mice lymphocytes [7, 8]. Furthermore, chicken were more resistant to *E. coli* infection after oral administration of *B. thermophilum* [9]. The infant feces isolate *B. thermophilum* RBL67 (RBL67) is a promising probiotic candidate which genome was sequenced [10]. The strain can grow under low oxygen, at pHs as low as 4.0 and at temperatures up to 47 °C. Further, it can reach high cell yield numbers in fermentation which makes it suitable to be applied in industrial fermentations [6, 11–14]. Furthermore, RBL67 decreases *S.* Typhimurium counts in an in vitro fermentation model of the gastrointestinal tract [14], reduces severity of rotavirus-associated diarrhea in suckling mice [15], and blocks invasion of *S.* Typhimurium and *L. monocytogenes* to human intestinal cell lines [13, 16]. However, the underlying mechanisms of RBL67-*Salmonella* interaction are not elucidated yet.

*Salmonella* species are a major cause of food-borne diseases with an estimated world-wide annual infection rate of 93.8 million cases and 155,000 deaths [17]. *Salmonella* usually infect humans after ingestion of contaminated food products [18, 19]. *Salmonella enterica* subsp. *enterica* serovar Typhimurium (*S.* Typhimurium) is a *Salmonella* serotype frequently encountered in clinical cases [18]. Its pathogenesis depends on multiple factors including motility and chemotaxis, adhesion, invasion and persistence. The majority of relevant virulence determinants are located on *Salmonella* pathogenicity islands (SPIs) and are regulated by a complex molecular network that transmits environmental signals of conditions prevailing in the host [18]. *Salmonella* invasion is dependent on the gut environment and is enhanced by low oxygen tension, high osmolarity, neutral pH and acetate, whereas cationic peptides, bile, propionate and butyrate suppress invasion [18, 20]. One of the key regulators for *Salmonella* invasion is HilA [21]. HilA expression is affected by environmental signals and enables *Salmonella* to express different invasive phenotypes under different conditions [18, 22, 23]. Modulation of the gut environment via pre- and/or probiotic treatments may alter the gene expression of pathogens like *Salmonella*, either indirectly via production of organic acids or directly via microbe-microbe interactions [2]. Indeed, probiotic strains were reported to modulate the transcriptional response of *Salmonella*. PhoP, a postulated repressor of *hil*A expression was activated and HilA was repressed during growth in the presence of supernatant of *Lactobacillus rhamnosus* GG [23]. However, information about modulation of gene expression in enteropathogens due to direct microbe-microbe interaction is still scarce and unraveling the transcriptomic response of these multifactorial interactions is challenging.

RNA-sequencing (RNA-seq) is a powerful tool to determine the transcriptional response of an organism in a complex culture because interference of signals from other organisms is limited [24]. In this study we investigated the potential of *B. thermophilum* RBL67 to modulate the transcriptome of *S.* Typhimurium N-15. The response of RBL67 and *Salmonella* Typhimurium N-15 in the co-culture was compared to mono-cultures using RNA-seq in attempt to provide insight in the protective mechanism of RBL67 against *Salmonella* infections.

## Methods

### Bacterial strains

*Salmonella* Typhimurium N-15 was isolated from a clinical case in Switzerland in 2007 and obtained from the National Reference Centre for Enteropathogenic Bacteria and Listeria (NENT; Zurich, Switzerland). *Bifidobacterium thermophilum* RBL67 (=LMG S-23614), originally isolated from infant feces [6], was obtained from our own culture collection.

### Batch fermentation conditions

Two sets of fermentations were performed, each set consisting of six fermentations. The first set was composed of three RBL67 mono-cultures and three RBL67-N-15 co-cultures. The second set consisted of three N-15 mono-cultures and another set of three RBL67-N-15co-cultures. The first set of three co-cultures was used for sampling RBL67-RNA at *t* = 5 h and the second for sampling N15-RNA at *t* = 4 h. Bacteria were cultured in 350 mL scale Sixfors bioreactors (Infors AG, Bottmingen, Switzerland) using 310 mL YCFA medium [25] supplemented with 6 g/L glucose (Sigma-Aldrich Chemie GmbH, Buchs, Switzerland). Fermentations were performed at 38 °C with stirring at 200 rpm for 24 h. A constant pH of 6.0 was maintained by automated addition of 2.5 M NaOH. Anaerobic conditions were ensured by purging the headspace with CO_2_. Fermentations were inoculated with 4 % (*v*/*v*) of a 16 h grown pre-culture. Pre-cultures were prepared by propagating strains twice in 10 mL YCFA medium in Hungate tubes to adapt the strains to the medium and anaerobic conditions. The pre-cultures were centrifuged (6000 × g, 5 min), washed in 0.1 % peptone water reduced with 0.05 % L-cysteine hydrochloride (VWR International AG, Dietikon, Switzerland) and resuspended in 2 mL peptone water before inoculation to the fermenter.

Growth was monitored by optical density measurements at 600 nm (OD_600_) using a Biochrom WPA CO8000 cell density meter (Biochrom, Cambridge, United Kingdom). Samples were taken hourly until the stationary growth phase was reached, with a final sample taken after 24 h. Metabolite and sugar concentrations were determined by HPLC analysis (Thermo Fisher Scientific, Wohlen, Switzerland) as described previously [26]. Carbon balance as % of carbons recovered was calculated on the basis of consumed glucose and produced organic acids. Viable cell counts of RBL67 were determined by plating appropriate dilutions on MRS agar (Biolife, Milan, Italy), supplemented with 0.05 % L-cysteine hydrochloride (MRS-C). Viable cell counts of N-15 were determined on MacConkey Agar No. 2 (Oxoid AG, Pratteln, Switzerland). Co-culture effluent samples were plated on MRS-C agar supplemented with 5 g L^−1^ mupirocin (VWR International AG, Dietikon, Switzerland) to select for RBL67 [27], and on MacConkey Agar No. 2 to select for N-15. MRS plates were incubated anaerobically using anaerobic gas pack systems (AnaeroGen TM, Oxoid AG) at 37 °C for 48 h. MacConkey Agar plates were incubated aerobically at 37 °C for 24 h.

Maximum specific growth rates were calculated for each replication separately (*N* = 3) from the slope of the curve of the log cell counts versus time during the exponential growth phase.

### Sampling for RNA extraction

RBL67 and N-15 mono- and co-culture samples were subjected to different procedures to allow optimal RNA extraction of both RBL67 and N-15.

Mono- and co-culture samples of N-15 cultures (20 mL each) were directly transferred to 20 mL 60 % glycerol (Sigma-Aldrich Chemie GmbH, Buchs, Switzerland) at −40 °C, kept on ice for 20 min and centrifuged for 15 min (3220 × g, 4 °C). The supernatant was discarded and the resulting pellets were immediately frozen at −80 °C until RNA extraction. Mono- and co-culture samples of RBL67 cultures were shortly centrifuged (10,000 × g, 20 s). The RBL67 mono-culture pellets were resuspended in 400 μl MRS-C and transferred to a pre-chilled screw cap tube, containing 500 mg glass beads (0.1 mm; Biospec Products Inc., Bartlesville, USA), 500 μl chloroform/phenol (1:1, *v*/*v*), 30 μl 3 M Na-acetate (pH 5.2) and 30 μl SDS 10 % [28]. The pellets of the RBL67 co-culture were resuspended in 12 mL of RNAprotect® Bacteria Reagent (Qiagen AG, Basel, Switzerland), incubated for 5 min at room temperature and centrifuged again (10,000 × g, 20 s). Both samples were then rapidly frozen in liquid nitrogen and stored at −80 °C until RNA extraction.

### RNA-extraction and ribosomal RNA depletion

Frozen pellets from N-15 samples were resuspended in 200 μl 10 mM Tris-buffer (pH 8.0). Total RNA was extracted using the High Pure RNA isolation kit (Roche Diagnostics, Rotkreuz, Switzerland), according to the manufacturer’s instructions. Total RNA of RBL67 mono- and co-culture samples was extracted using a phenol/chloroform extraction method [28], followed by a purification using the High Pure RNA isolation kit (Roche Diagnostics). Prior to RNA extraction the sample from the RBL67 co-culture was resuspended in MRS-C medium and transferred to a pre-chilled mix of 500 mg glass beads (Biospec Products Inc.) and TRI Reagent® (Life Technologies Europe BV, Zug, Switzerland).

RNA quantity and purity was determined on a NanoDrop 1000 Spectrophotometer (Thermo Fisher Scientific, Washington, USA) and RNA integrity was tested with an Agilent 2100 Bioanalyzer (Agilent, Basel, Switzerland). RBL67 samples with a RNA integrity number (RIN) ≥ 9.5 and a 16S/23S-rRNA ratio ≥1.6 were used for ribosomal RNA depletion and subsequent RNA-sequencing. Due to the aberrant nature of ribosomal RNA of *S.* Typhimurium [29], the RIN value and the 16S/23S-rRNA ratio could not be calculated for N-15. Hence we selected samples which were comparable to the profiles reported previously for *Salmonella* [30], i.e. a straight zero line (indicating no RNA degradation), absence of 23S RNA and appearance of two additional peaks neighboring the 16S peak.

Depletion of ribosomal RNA from 10 μg total RNA was performed using the MICROBExpress™ Bacterial mRNA Enrichment Kit (Life Technologies Europe BV, Zug, Switzerland) according to the manufacturer’s instructions. Additionally, EDTA (1 mM) was added to chelate divalent cations present in the RNA solution.

### RNA-sequencing

RNA-sequencing was performed on an Illumina HiSeq 2000 sequencer (Illumina Inc., California, USA) at the Functional Genomics Center Zurich (FGCZ). Libraries were prepared using the TruSeq Stranded mRNA Sample Prep Kit (Illumina) according to the manufacturer’s protocol. The libraries were qualitatively and quantitatively checked using a Qubit® (1.0) Fluorometer (Life Technologies Europe BV, Zug, Switzerland) and a Bioanalyzer 2100 (Agilent, Basel, Switzerland) and were subsequently normalized at 10 nM in Tris-Cl (10 mM, pH 8.5) containing 0.1 % Tween20. Cluster generation was performed using the TruSeq SR Cluster Kit v3-cBot-HS (Illumina) using 8 pM of pooled normalized libraries on the cBOT and stranded sequencing of 100 bp was done using the TruSeq SBS Kit v3-HS (Illumina). Each set of samples (*N* = 6) was analyzed in a separate sequencing lane.

### RNA-Seq data analysis

Illumina raw data reads (100 bp) were separated by barcode and mapped against the genome of RBL67 (GenBank accession no. CP004346) or *Salmonella* Typhimurium LT2 (GenBank accession no. AE006468) using the CLC Genomics Workbench 6.5.1 (CLCbio, Aarhus, Denmark) applying the default settings. Maximum allowance of mismatches was set at 2, minimum length fraction at 0.9 and minimum similarity fraction at 0.8.

Statistical analysis for differential gene expression of the mono- and co-cultures was done with the statistical software R (http://www.R-project.org) using the GLM method [31] included in the Bioconductor EdgeR software package [32–34] based on negative binomial distribution. Genes with low read numbers (sum of reads in all samples <3 counts per million (cpm)) or with high read numbers (number of reads >50,000 cpm in each sample) were filtered out before data normalization. A false discovery rate (FDR) value <0.05 and a differential expression of at least 2 fold (1 < log_2_ ratio < −1) was used as cut off for significant differentially expressed genes in mono-culture and co-culture [35]. Proteins of RBL67 and LT2 were assigned to gene ontology categories (GO) using Blast2GO at standard settings [36]. GO categories enrichment analyses were performed and visualized using the BiNGO plugin [37] in Cytoscape (v.3.0.1, [38]) applying the hypergeometric test with Benjamini and Hochberg false discovery rate correction option. The significance cutoff for overrepresented gene ontology categories was a corrected *p*-value of <0.05.

Virulence factors of *Salmonella* LT2 were identified by genome wide blast against the virulence factor database (VFDB) [39], using a cut off E-value of 1^−20^. Significant enrichment of virulence factors was calculated using the Fisher’s Exact Test Calculator for 2 × 2 Contingency at www.research.microsoft.com/en-us/um/redmond/projects/mscompbio/fisherexacttest/.

The RNAseq data discussed in this publication have been deposited in NCBI’s Gene Expression Omnibus [40] and are accessible through GEO Series accession number GSE65716 (http://www.ncbi.nlm.nih.gov/geo/query/acc.cgi?acc=GSE65716).

### Statistical analysis

Statistical analysis for cell counts (log_10_ transformation) and growth rates were performed using JMP 10.0 (SAS Institute., Cary, NC). Cell counts and maximum specific growth rates of mono-and co-cultures were tested for significant differences using the non-parametric Kruskal-Wallis (*P*-value <0.05).

## Results

### Growth characteristics of RBL67 in mono- and co-culture with N-15

**Fig. 1:**
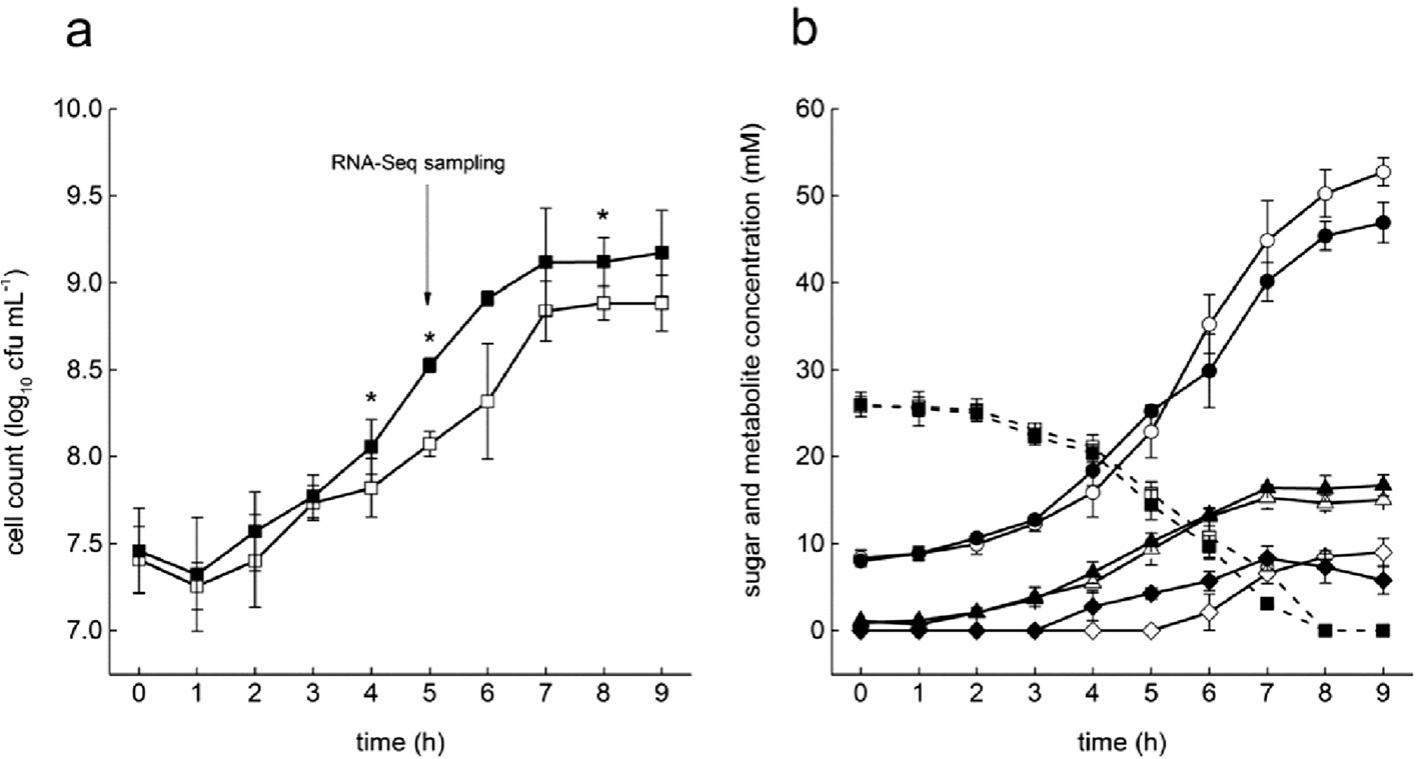
Cell counts and metabolic profiles of RBL67 in mono-culture and in co-cultures with N-15. **a** Cell counts in mono- (*open symbols*) and co-culture (*closed symbols*). **b** Metabolite concentration in mono- (*open symbols*) and co-culture (*closed symbols*). Means ± SD from three biological replicates are presented. *Cell counts significantly different between mono- and co-culture with the non-parametric Kruskal-Wallis Test (*P* < 0.05); *square*: glucose; *circle*: acetate; *triangle*: lactate and *diamonds*: formate

To analyze interactions between *B. thermophilum* RBL67 and *Salmonella* N-15, both strains were grown in pH controlled mono- and co-cultures (pH 6.0) and growth characteristics were compared. The maximum specific growth rate of RBL67 in mono-culture (μ_max_ = 0.26 ± 0.05 h^−1^) was significantly lower compared to that in co-culture (μ_max_ = 0.33 ± 0.01 h^−1^). The stationary growth phase was reached after approximately 8 h in both cultures, with final cell counts of 8.88 ± 0.10 log_10_ cfu mL^−1^ and 9.12 ± 0.14 log_10_ cfu mL^−1^ in the mono- and co-culture, respectively (Fig. 1a).

Glucose consumption and metabolite profiles were similar for RBL67 in mono- and co-culture (Fig. 1b). In both cultures, glucose was depleted after 8 h which corresponded to the onset of the stationary growth phase, indicating growth limitation by the carbon source. The main metabolites produced in mono-cultures were 50 ± 3 mM acetate, 15 ± 1 mM lactate, and 9 ± 0.3 mM formate after 8 h, corresponding to a calculated carbon recovery of 103 %. A slightly lower acetate concentration was observed in co-culture: 45 ± 2 mM acetate. Further, 16 ± 2 mM lactate and 7 ± 2 mM formate were produced after 8 h, corresponding to a carbon recovery of 100 %.

Taken together, RBL67 growth was slightly enhanced in the co-culture with *Salmonella* compared to mono-culture and only small differences in organic acid production were observed.

### Global transcriptional response of RBL67 to co-culture with N-15

To elucidate the response of RBL67 to N-15 on a global level, the transcriptome profiles of *B. thermophilum* RBL67 grown in mono- and in co-culture were compared. Samples were taken after 5 h of growth, a time point at which RBL67 displayed exponential growth in both cultures (Fig. 1). Viable RBL67 cell counts at harvesting point were 8.07 ± 0.07 and 8.53 ± 0.04 log_10_ cfu mL^−1,^ in the mono- and in co-culture, respectively. RNA sequencing of RBL67 cultures resulted in a mean read number of 37,365,651 and 31,752,403 for mono- and co-cultures, respectively. Thereof, 93 % of the reads deriving from the mono-cultures and 79 % of the reads from the co-cultures could be mapped onto the RBL67 genome. Differential gene expression analysis revealed 57 genes being significantly differentially expressed in mono- compared to co-cultures (Tables 1 and 2). An operon involved in lipid export (D805_0155-D805_0157), sugar transport (D805_1600-D805_1602), and an operon of undefined function (D805_1659-D805_1660), together with its putative regulator of the HxlR family (D805_1658) were higher expressed in co- culture (Table 1). Further, a stress response was triggered in co-cultures as revealed by higher expression of the heat shock protein regulator HspR (D805_1678), the SOS-response repressor and protease LexA (D805_0599) and the protease ClpB (D805_1594). The latter gene harbors a HspR-associated inverted repeat (HAIR) in its upstream region and is therefore likely activated by HspR. Additional functions of RBL67 genes higher expressed in co-cultures with *Salmonella* N-15 were related to metal transport (D805_1209) and amino acid metabolism (D805_1238 and D805_1530), including a glutamate-5-kinase (D805_1238) which catalyzes the first step for proline biosynthesis from glutamate.

**Table 1:** *Bifidobacterium thermophilum* RBL67 genes higher expressed in co-culture with N-15 compared to mono-culture

Locus tag	Function	logFC^a^	logCPM^b^	FDR^c^
D805_0058	Oligopeptide transport ATP-binding protein OppF (TC 3.A.1.5.1)	−1.52	4.09	4E-06
D805_0077	hypothetical protein	−1.17	6.36	4.8E-07
D805_0155	Transcriptional regulator, MarR family	−1.95	6.80	1.2E-17
D805_0156	hypothetical protein	−1.50	3.64	0.00401
D805_0157	Lipid A export ATP-binding/permease protein MsbA	−1.17	8.10	6.2E-06
D805_0382	hypothetical protein	−1.13	7.94	1.4E-06
D805_0466	FIG 00672402: hypothetical protein	−1.00	7.21	9.5E-08
D805_0503	possible conserved integral membrane protein	−1.14	3.08	0.01355
D805_0599	SOS-response repressor and protease LexA (EC 3.4.21.88)	−1.24	6.59	7.9E-09
D805_0600	hypothetical protein	−2.14	6.18	2.6E-27
D805_0707	Inner membrane protein	−1.14	6.16	3.4E-06
D805_1209	Zinc ABC transporter, periplasmic-binding protein ZnuA	−1.15	4.69	1.3E-05
D805_1238	Cystathionine beta-synthase (EC 4.2.1.22)	−1.23	6.23	2.7E-09
D805_1392	putative aminotransferase	−1.23	4.66	1.2E-05
D805_1393	hypothetical protein	−1.03	4.30	0.00118
D805_1530	Glutamate 5-kinase (EC 2.7.2.11)	−1.08	8.00	3.9E-09
D805_1531	COG0536: GTP-binding protein Obg	−1.03	9.16	2.5E-06
D805_1591	DNA recombination protein RmuC	−1.27	7.58	3.6E-12
D805_1594	ClpB protein	−1.06	8.68	2.1E-06
D805_1600	Maltodextrin glucosidase (EC 3.2.1.20)	−1.17	6.23	5.6E-08
D805_1601	ABC-type sugar transport system, permease component	−1.85	4.02	3.9E-09
D805_1602	MSM (multiple sugar metabolism) operon regulatory protein	−1.68	3.28	1.1E-05
D805_1621	Sortase A, LPXTG specific	−1.09	3.52	0.00557
D805_1622	hypothetical protein	−1.44	4.42	5.6E-08
D805_1637	COG family: predicted phosphohydrolases	−1.63	6.91	1.8E-21
D805_1658	Transcriptional regulator, HxlR family	−1.07	4.37	0.00624
D805_1659	Rrf2-linked NADH-flavin reductase	−2.01	5.23	2.9E-14
D805_1660	COG2110, Macro domain, possibly ADP-ribose binding module	−1.83	4.90	2.4E-16
D805_1678	HspR, transcriptional repressor of DnaK operon	−1.08	5.37	4.9E-08
D805_1702	transport protein	−1.80	6.58	3.5E-23

^a^ *logFC* log_2_ fold change, ^b^ *logCPM* log_2_ counts per million, ^c^ *FDR* false discovery rate

**Table 2:** *Bifidobacterium thermophilum* RBL67 genes higher expressed in mono-culture compared to co-culture with N-15

Locus tag	Function	logFC^a^	logCPM^b^	FDR^c^
D805_0063	FIG 00519111: hypothetical protein	1.76	5.67	1.8E-12
D805_0064	HTH domain protein	2.18	3.98	4.1E-09
D805_0075	hypothetical protein	1.04	5.15	0.00011
D805_0178	Ribonucleotide reductase of class Ib (aerobic), alpha subunit (EC 1.17.4.1)	1.21	7.11	0.04089
D805_0341	Transcriptional regulator, GntR family domain/Aspartate aminotransferase (EC 2.6.1.1)	1.93	3.72	0.02045
D805_0345	Manganese transport protein MntH	1.84	3.71	0.04055
D805_0351	Glycosyl transferase, group 2 family protein	1.49	4.48	0.00041
D805_0352	Glycosyltransferase (EC:2.4.1.-)	1.74	4.96	1.3E-07
D805_0354	glycosyl transferase, group 1 family protein	2.07	4.79	3.0E-10
D805_0355	hypothetical protein	1.34	6.14	4.5E-06
D805_0356	Glycosyltransferase (EC 2.4.1.-)	2.14	4.96	1.5E-15
D805_0512	hypothetical protein	2.01	2.39	0.03026
D805_0524	D-lactate dehydrogenase (EC 1.1.1.28)	2.85	3.15	0.00049
D805_0525	Aspartate aminotransferase (EC 2.6.1.1)	1.91	3.11	0.01352
D805_0652	Oligopeptide transport system permease protein OppC (EC 3.A.1.5.1)	2.25	2.47	0.00227
D805_0656	hypothetical protein	1.57	3.01	0.01267
D805_0665	hypothetical protein	1.18	5.85	2.0E-05
D805_0693	Acetyltransferase, GNAT family	2.23	6.96	2.7E-31
D805_0694	hypothetical protein	2.80	3.46	1.5E-14
D805_0698	hypothetical protein	1.19	4.02	0.00369
D805_0837	putative TraA-like conjugal transfer protein	2.75	3.13	0.00038
D805_0885	Ferric iron ABC transporter, iron-binding protein	1.63	2.52	0.00624
D805_0928	hypothetical protein	1.90	2.59	0.00374
D805_0948	hypothetical protein	2.08	2.96	0.00118
D805_1220	hypothetical protein	2.60	3.03	0.00015
D805_1313	Methionine ABC transporter permease protein	1.52	2.03	0.04091
D805_1771	hypothetical protein	1.01	4.56	0.01571

^a^ *logFC* log_2_ fold change, ^b^ *logCPM* log_2_ counts per million, ^c^ *FDR* false discovery rate

Twenty-seven genes were down regulated in co-cultures compared to monoculture, of which 12 were classified as hypothetical proteins (Table 2). A putative operon encoding glycosyltransferases (ORF D805_0351-D805_0356), three genes involved in amino acid metabolism (D805_0341, D805_0525 and D805_1313), including the glutamate producing enzyme aspartate aminotransferase (EC 2.6.1.1) and two metal transporters (D805_0345 and D805_0885) were higher expressed.

Mapping the co-culture reads to the *Salmonella* Typhimurium LT2 genome resulted in less than 5 million reads mapped (data not shown), indicating that the majority of the RNA isolated form the co-culture after 5 h consisted of bifidobacterial RNA.

### Growth characteristics of N-15 in mono- and co-culture with RBL67

**Fig. 2:**
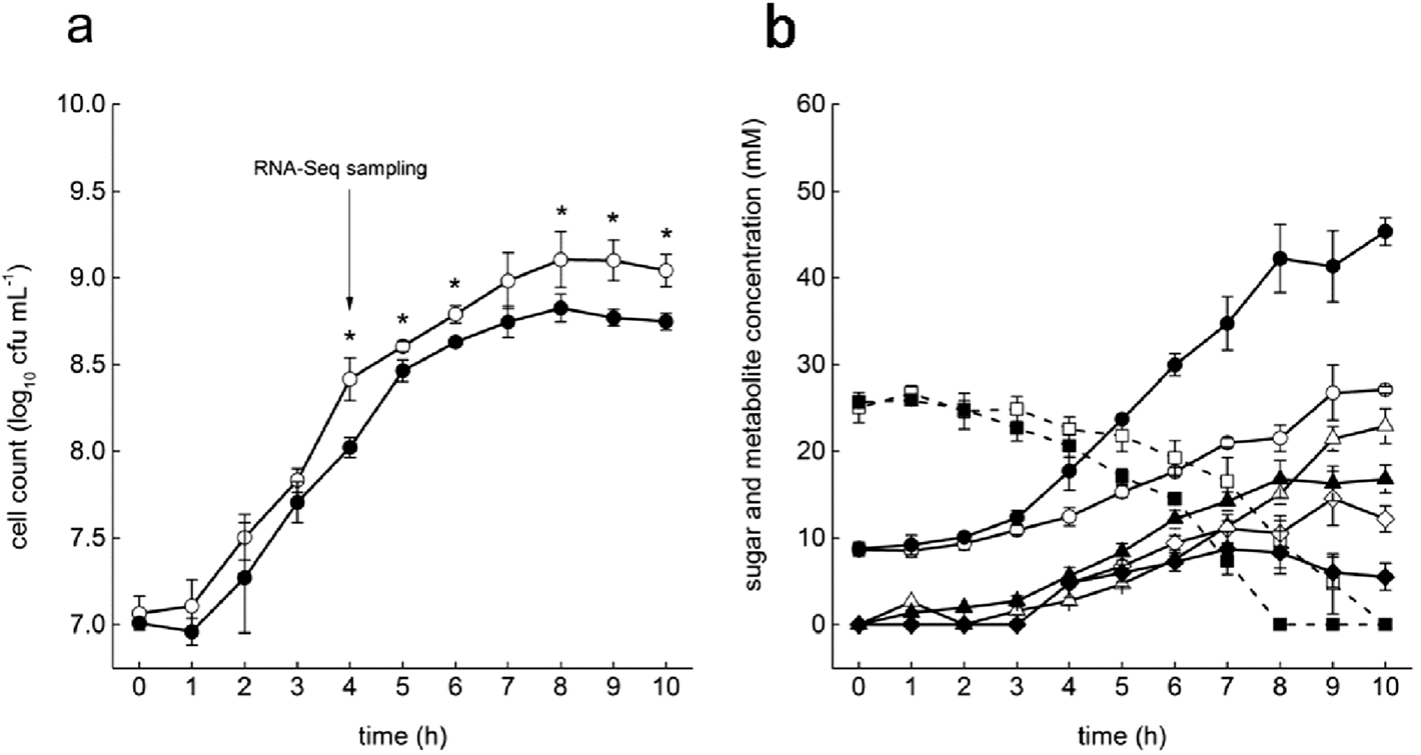
Cell counts and metabolic profiles of N-15 in mono-culture and in co-cultures with RBL67. **a** Cell counts in mono- (*open symbols*) and co-culture (*closed symbols*). **b** Metabolite concentration in mono- (*open symbols*) and co-culture (*closed symbols*). Means ± SD from three biological replicates are presented. *Cell counts significantly different between mono- and co-culture with the non-parametric Kruskal-Wallis Test (*P* < 0.05); *square*: glucose; *circle*: acetate; *triangle*: lactate and *diamonds*: formate

In a next step *Salmonella* N-15 was grown in a mono- and co-culture with RBL67, the latter being a repetition of the co-culture presented above. *Salmonella* N-15 had similar maximum specific growth rates of μ_max_ = 0.39 ± 0.02 h^−1^ and 0.38 ± 0.04 h^−1^ in mono- and co-culture, respectively. In the late exponential phase after approximately 5 h of fermentation, the growth rate in the co-culture was smaller compared to mono-culture (Fig. 2a). The difference in growth resulted in a higher *Salmonella* cell count of 9.10 ± 0.16 log_10_ cfu mL^−1^ in the mono-culture compared to 8.82 ± 0.08 log_10_ cfu mL^−1^ in the co-culture. Glucose was depleted after 10 and 8 h for mono- and co-cultures, respectively (Fig. 2b). The main metabolites produced by N-15 in mono-culture were 27 ± 0.4 mM acetate, 23 ± 2 mM lactate and 12 ± 2 mM formate, corresponding to a calculated carbon recovery of 93 %. The metabolite production in the co-culture was: 42 ± 4 mM acetate, 17 ± 2 mM lactate and 8 ± 3 mM formate. These values are virtually the same to those from the first co-culture experiments (Fig. 1b). The calculated carbon mass balance in the co-culture was 99 %.

*Salmonella* reached slightly lower cell numbers in the co-culture with RBL67 compared to its mono-culture, but was further not affected by the presence of RBL67 concerning growth speed.

### Global transcriptional response of N-15 to co-culture with RBL67

Because RNA-seq analyses of the co-culture after 5 h growth resulted in low read mapping the transcriptome of N-15 in mono-a-culture and in co-culture with RBL67 was analyzed after 4 h growth. This time-point corresponds to cell counts of 8.42 ± 0.12 and 8.02 ± 0.06 log_10_ cfu mL^−1^ for mono- and co-cultures, respectively. Acetate concentrations at sampling point was 17.8 ± 2.2 mM in the co-culture, slightly higher than the 12.5 ± 1.1 mM in the mono-culture Moreover, at this point *Salmonella* is growing exponentially and at comparable speed in both cultures (Fig. 2b). From the total mean read numbers of 38,838,013 (mono-culture) and 30,020,491 (co-culture), 91 and 52 % could be mapped onto the genome and plasmid of the sequenced strain *Salmonella* Typhimurium LT2, respectively. In total 701 genes were higher expressed in mono- culture and 1278 genes in the co-culture (Additional file 1: Tables S1 and S2).

GO category enrichment analysis revealed 88 categories significantly overrepresented in co-culture of which 47 belonged to the cluster “biological processes”, 29 to “molecular function” and 29 to “cellular component”. Within the cluster biological processes the categories “localization” (GO:051179), “establishment of localization” (GO:051234) and “transport” (GO:006810) were significantly overrepresented (Table 3). Detailed analysis of these categories revealed that they each contained the same 281 genes. At a lower hierarchical level, the category “protein secretion by the type III secretion system” was highly overrepresented (GO:030254, *N* = 49 genes). Further, “siderophore transport” and “carbohydrate transport systems”, including “PEP-dependent sugar phosphotransferase systems” (GO:009401, *N* = 33) were overrepresented in co-culture. Other categories overrepresented in biological processes included “multi-organism process” (GO:051704, *N* = 47), “pathogenesis” (GO:009405, *N* = 26) and “interspecies interaction between organisms” (GO:044419, *N* = 35). The majority of the genes (*N* = 26) in the latter category were also found in GO:052049: “interaction with host via protein secreted by type III secretion system”. The 26 genes assigned to this category were also present in already mentioned GO:030254: “type III secretion system” category.

**Table 3:** Gene Ontology (GO) categories of the *Salmonella* Typhimurium N-15 transcriptome significantly overrepresented in the co-culture with RBL67 compared to mono-culture

GO category	*p*-value	N_genes_ in category	Description of category
Biological process			
GO:051234	4.77E-28	281	establishment of localization
GO:006810	4.77E-28	281	transport
GO:051179	6.29E-25	281	localization
GO:030254	8.34E-15	42	protein secretion by the type III secretion system
GO:051704	1.80E-14	47	multi-organism process
GO:051701	3.20E-12	35	interaction with host
GO:044419	3.20E-12	35	interspecies interaction between organisms
GO:044403	3.20E-12	35	symbiosis, encompassing mutualism through parasitism
GO:008643	8.15E-11	52	carbohydrate transport
GO:046903	4.51E-10	49	secretion
GO:032940	4.51E-10	49	secretion by cell
GO:009306	4.51E-10	49	protein secretion
GO:052047	5.68E-10	26	interaction with other organism via secreted substance involved in symbiotic interaction
GO:052049	5.68E-10	26	interaction with host via protein secreted by type III secretion system
GO:052048	5.68E-10	26	interaction with host via secreted substance involved in symbiotic interaction
GO:052210	5.68E-10	26	interaction with other organism via protein secreted by type III secretion system involved in symbiotic interaction
GO:044046	5.68E-10	26	interaction with host via substance released outside of symbiont
GO:051649	4.63E-09	49	establishment of localization in cell
GO:051641	8.25E-09	49	cellular localization
GO:009405	9.76E-08	26	pathogenesis
GO:015031	2.55E-06	49	protein transport
GO:045184	2.55E-06	49	establishment of protein localization
GO:033036	2.96E-06	50	macromolecule localization
GO:008104	3.67E-06	49	protein localization
GO:009401	8.19E-06	33	phosphoenolpyruvate-dependent sugar phosphotransferase system
GO:007047	2.10E-04	12	cellular cell wall organization
GO:045229	2.10E-04	12	external encapsulating structure organization
GO:071555	7.60E-04	12	cell wall organization
GO:009242	2.19E-03	7	colanic acid biosynthetic process
GO:052126	2.19E-03	7	movement in host environment
GO:052192	2.19E-03	7	movement in environment of other organism involved in symbiotic interaction
GO:044409	2.19E-03	7	entry into host
GO:046377	2.19E-03	7	colanic acid metabolic process
GO:051828	2.19E-03	7	entry into other organism involved in symbiotic interaction
GO:022610	3.83E-03	17	biological adhesion
GO:007155	3.83E-03	17	cell adhesion
GO:030001	6.91E-03	33	metal ion transport
GO:006814	8.26E-03	16	sodium ion transport
GO:009235	1.23E-02	14	cobalamin metabolic process
GO:009236	1.23E-02	14	cobalamin biosynthetic process
GO:015891	2.34E-02	5	siderophore transport
GO:019184	2.34E-02	5	nonribosomal peptide biosynthetic process
GO:006811	2.34E-02	47	ion transport
GO:015672	2.61E-02	27	monovalent inorganic cation transport
GO:006812	3.45E-02	37	cation transport
GO:006778	4.86E-02	16	porphyrin metabolic process
GO:006779	4.86E-02	16	porphyrin biosynthetic process
Molecular function			
GO:005215	4.79E-20	215	transporter activity
GO:015144	1.57E-09	39	carbohydrate transmembrane transporter activity
GO:022892	1.31E-08	111	substrate-specific transporter activity
GO:022891	9.42E-08	99	substrate-specific transmembrane transporter activity
GO:051119	9.81E-08	34	sugar transmembrane transporter activity
GO:022857	1.61E-07	107	transmembrane transporter activity
GO:008324	1.82E-07	64	cation transmembrane transporter activity
GO:005402	1.79E-06	29	cation:sugar symporter activity
GO:015075	5.82E-06	70	ion transmembrane transporter activity
GO:015291	6.89E-06	44	secondary active transmembrane transporter activity
GO:015294	6.89E-06	34	solute:cation symporter activity
GO:015293	6.89E-06	34	symporter activity
GO:015295	6.89E-06	27	solute:hydrogen symporter activity
GO:005351	6.89E-06	27	sugar:hydrogen symporter activity
GO:022804	5.84E-05	71	active transmembrane transporter activity
GO:015082	6.07E-03	13	di-, tri-valent inorganic cation transmembrane transporter activity
GO:046873	7.20E-03	23	metal ion transmembrane transporter activity
GO:022890	9.53E-03	28	inorganic cation transmembrane transporter activity
GO:015149	1.06E-02	8	hexose transmembrane transporter activity
GO:015145	1.06E-02	8	monosaccharide transmembrane transporter activity
GO:015343	2.20E-02	5	siderophore-iron transmembrane transporter activity
GO:042927	2.20E-02	5	siderophore transporter activity
GO:005381	2.59E-02	8	iron ion transmembrane transporter activity
GO:046915	2.59E-02	11	transition metal ion transmembrane transporter activity
GO:042879	2.59E-02	6	aldonate transmembrane transporter activity
GO:015128	2.59E-02	6	gluconate transmembrane transporter activity
GO:005506	3.93E-02	16	iron ion binding
GO:046943	4.82E-02	26	carboxylic acid transmembrane transporter activity
GO:005342	4.82E-02	26	organic acid transmembrane transporter activity
Cellular component			
GO:016020	6.15E-12	381	membrane
GO:030257	3.05E-10	26	type III protein secretion system complex
GO:005886	2.84E-08	314	plasma membrane
GO:044425	5.28E-06	129	membrane part
GO:016021	5.28E-06	124	integral to membrane
GO:031224	5.28E-06	124	intrinsic to membrane
GO:009279	4.20E-05	47	cell outer membrane
GO:019867	3.67E-04	47	outer membrane
GO:043234	4.76E-04	50	protein complex
GO:009289	9.12E-04	15	pilus
GO:044462	2.45E-03	104	external encapsulating structure part
GO:043190	3.32E-02	4	ATP-binding cassette (ABC) transporter complex

In the “molecular function” cluster, “transporter activity” was significantly overrepresented (GO:005215, *N* = 215), with transmembrane transporters being highly abundant (Table 3) The “cellular component” cluster included membrane-associated functions (GO:016020, *N* = 381) including again the overrepresented “type III protein secretion system complex” (GO:030257, *N* = 26) (Table 3). The GO enrichment in the “molecular function” and “cellular component” clusters was similar to that of the “biological processes” cluster.

Summarizing, the transcriptomic analyses of N-15 in co-culture compared to mono-culture revealed responses involved in carbohydrate and metal transport and in extracellular function, mainly secretion of proteins (secretion, cell wall organization, interaction with other organisms).

In the mono-culture, 133 GO categories were significantly enriched of which 101 were assigned to “biological processes”, 6 into “molecular functions” and 26 into “cellular components” (Table 4). Overrepresented categories included “cellular process” in the biological processes cluster (GO:009987; *N* = 276) and “structural molecule activity” in the molecular function cluster (GO:005198; *N* = 39). In the cellular components cluster the “intracellular parts” (GO:044424; *N* = 391), which includes the categories “ribosome” (GO:005840; *N* = 33) and “flagellum” (GO:019861; *N* = 19), were overrepresented. GO category 019861 (“flagellum”) includes the flagellar biosynthesis encoding operons *flg* and *fli* and *cheZ*. Overall, GO-categories related to flagella and to cell growth like “cellular processes” and “ribosomes” and were significantly overrepresented in the mono-culture transcriptome of *Salmonella* N-15.

**Table 4:** Gene Ontology (GO) categories of *Salmonella* Typhimurium N-15 transcriptome significantly overrepresented in the mono-culture compared to co-culture with RBL67

GO category	*p*-value	N_genes_ in category	Description of category
Biological process			
GO:044249	1.69E-20	168	cellular biosynthetic process
GO:009058	2.27E-18	174	biosynthetic process
GO:010467	1.16E-11	70	gene expression
GO:009987	2.05E-11	276	cellular process
GO:044237	1.12E-10	236	cellular metabolic process
GO:006412	1.07E-09	49	translation
GO:044238	2.26E-08	227	primary metabolic process
GO:034645	5.65E-08	74	cellular macromolecule biosynthetic process
GO:009059	1.43E-07	75	macromolecule biosynthetic process
GO:044267	2.58E-07	66	cellular protein metabolic process
GO:006633	1.86E-06	12	fatty acid biosynthetic process
GO:019538	4.61E-06	82	protein metabolic process
GO:044260	5.80E-06	118	cellular macromolecule metabolic process
GO:008299	5.80E-06	12	isoprenoid biosynthetic process
GO:006720	5.80E-06	12	isoprenoid metabolic process
GO:008610	7.04E-06	32	lipid biosynthetic process
GO:008152	1.73E-05	286	metabolic process
GO:044255	1.80E-05	32	cellular lipid metabolic process
GO:043170	1.93E-05	138	macromolecule metabolic process
GO:006629	2.55E-05	34	lipid metabolic process
GO:006631	4.06E-05	12	fatty acid metabolic process
GO:048870	4.72E-04	13	cell motility
GO:051674	4.72E-04	13	localization of cell
GO:001539	4.72E-04	13	ciliary or flagellar motility
GO:044283	6.74E-04	62	small molecule biosynthetic process
GO:006928	8.07E-04	13	cellular component movement
GO:006350	9.66E-04	9	transcription
GO:043064	1.53E-03	8	flagellum organization
GO:009141	2.48E-03	10	nucleoside triphosphate metabolic process
GO:016070	2.77E-03	30	RNA metabolic process
GO:009108	3.33E-03	17	coenzyme biosynthetic process
GO:030030	3.43E-03	8	cell projection organization
GO:009142	4.02E-03	9	nucleoside triphosphate biosynthetic process
GO:006351	5.55E-03	7	transcription, DNA-dependent
GO:040011	5.89E-03	17	locomotion
GO:044281	8.06E-03	106	small molecule metabolic process
GO:009296	8.41E-03	6	flagellum assembly
GO:034641	9.65E-03	117	cellular nitrogen compound metabolic process
GO:006139	9.87E-03	77	nucleobase, nucleoside, nucleotide and nucleic acid metabolic process
GO:032774	9.87E-03	7	RNA biosynthetic process
GO:019720	9.87E-03	7	Mo-molybdopterin cofactor metabolic process
GO:032324	9.87E-03	7	molybdopterin cofactor biosynthetic process
GO:043545	9.87E-03	7	molybdopterin cofactor metabolic process
GO:051189	9.87E-03	7	prosthetic group metabolic process
GO:006777	9.87E-03	7	Mo-molybdopterin cofactor biosynthetic process
GO:016053	1.01E-02	31	organic acid biosynthetic process
GO:046394	1.01E-02	31	carboxylic acid biosynthetic process
GO:009219	1.10E-02	4	pyrimidine deoxyribonucleotide metabolic process
GO:009394	1.10E-02	4	2′-deoxyribonucleotide metabolic process
GO:042180	1.26E-02	58	cellular ketone metabolic process
GO:030031	1.55E-02	6	cell projection assembly
GO:006732	1.55E-02	20	coenzyme metabolic process
GO:042559	1.55E-02	7	pteridine and derivative biosynthetic process
GO:042558	1.55E-02	7	pteridine and derivative metabolic process
GO:046034	1.55E-02	7	ATP metabolic process
GO:015985	1.55E-02	7	energy coupled proton transport, down electrochemical gradient
GO:015986	1.55E-02	7	ATP synthesis coupled proton transport
GO:006119	1.55E-02	7	oxidative phosphorylation
GO:006754	1.55E-02	7	ATP biosynthetic process
GO:044271	1.65E-02	52	cellular nitrogen compound biosynthetic process
GO:006950	1.72E-02	26	response to stress
GO:022607	1.93E-02	16	cellular component assembly
GO:044085	2.28E-02	22	cellular component biogenesis
GO:009152	2.52E-02	10	purine ribonucleotide biosynthetic process
GO:009201	2.53E-02	7	ribonucleoside triphosphate biosynthetic process
GO:009206	2.53E-02	7	purine ribonucleoside triphosphate biosynthetic process
GO:009145	2.53E-02	7	purine nucleoside triphosphate biosynthetic process
GO:006807	2.99E-02	121	nitrogen compound metabolic process
GO:019748	3.10E-02	4	secondary metabolic process
GO:009234	3.10E-02	4	menaquinone biosynthetic process
GO:009233	3.10E-02	4	menaquinone metabolic process
GO:042362	3.10E-02	4	fat-soluble vitamin biosynthetic process
GO:042371	3.10E-02	4	vitamin K biosynthetic process
GO:042373	3.10E-02	4	vitamin K metabolic process
GO:006775	3.10E-02	4	fat-soluble vitamin metabolic process
GO:009150	3.10E-02	10	purine ribonucleotide metabolic process
GO:006164	3.35E-02	11	purine nucleotide biosynthetic process
GO:009165	3.40E-02	15	nucleotide biosynthetic process
GO:009205	3.58E-02	7	purine ribonucleoside triphosphate metabolic process
GO:009199	3.58E-02	7	ribonucleoside triphosphate metabolic process
GO:009144	3.58E-02	7	purine nucleoside triphosphate metabolic process
GO:019752	3.59E-02	53	carboxylic acid metabolic process
GO:043436	3.59E-02	53	oxoacid metabolic process
GO:009211	3.59E-02	3	pyrimidine deoxyribonucleoside triphosphate metabolic process
GO:009200	3.59E-02	3	deoxyribonucleoside triphosphate metabolic process
GO:009120	3.59E-02	3	deoxyribonucleoside metabolic process
GO:046125	3.59E-02	3	pyrimidine deoxyribonucleoside metabolic process
GO:009221	3.59E-02	3	pyrimidine deoxyribonucleotide biosynthetic process
GO:009263	3.59E-02	3	deoxyribonucleotide biosynthetic process
GO:009265	3.59E-02	3	2′-deoxyribonucleotide biosynthetic process
GO:009260	3.68E-02	10	ribonucleotide biosynthetic process
GO:006163	3.85E-02	11	purine nucleotide metabolic process
GO:019438	3.88E-02	12	aromatic compound biosynthetic process
GO:015992	3.97E-02	8	proton transport
GO:006818	3.97E-02	8	hydrogen transport
GO:006082	4.00E-02	54	organic acid metabolic process
GO:090304	4.05E-02	52	nucleic acid metabolic process
GO:043648	4.38E-02	9	dicarboxylic acid metabolic process
GO:016043	4.38E-02	22	cellular component organization
GO:009259	4.64E-02	10	ribonucleotide metabolic process
GO:032787	4.92E-02	16	monocarboxylic acid metabolic process
Molecular function			
GO:005198	2.25E-10	39	structural molecule activity
GO:003735	1.05E-08	32	structural constituent of ribosome
GO:046983	1.50E-02	10	protein dimerization activity
GO:003774	2.16E-02	10	motor activity
GO:016810	2.24E-02	16	hydrolase activity, acting on carbon-nitrogen (but not peptide) bonds
GO:016814	3.39E-02	6	hydrolase activity, acting on carbon-nitrogen (but not peptide) bonds, in cyclic amidines
Cellular component			
GO:044424	7.14E-22	391	intracellular part
GO:005622	8.72E-21	395	intracellular
GO:005737	1.19E-16	371	cytoplasm
GO:043228	7.58E-16	53	non-membrane-bounded organelle
GO:043232	7.58E-16	53	intracellular non-membrane-bounded organelle
GO:043229	7.58E-16	57	intracellular organelle
GO:043226	7.58E-16	57	organelle
GO:005840	1.95E-10	33	ribosome
GO:030529	5.54E-10	33	ribonucleoprotein complex
GO:044444	1.68E-09	41	cytoplasmic part
GO:032991	2.06E-07	59	macromolecular complex
GO:019861	1.39E-06	19	flagellum
GO:009288	1.47E-05	15	bacterial-type flagellum
GO:044422	3.09E-04	16	organelle part
GO:033279	9.66E-04	9	ribosomal subunit
GO:016469	3.31E-03	8	proton-transporting two-sector ATPase complex
GO:042995	7.19E-03	19	cell projection
GO:044446	7.85E-03	9	intracellular organelle part
GO:015934	8.09E-03	5	large ribosomal subunit
GO:009426	1.60E-02	3	bacterial-type flagellum basal body, distal rod
GO:009424	1.60E-02	3	bacterial-type flagellum hook
GO:009317	1.60E-02	3	acetyl-CoA carboxylase complex
GO:044463	3.10E-02	7	cell projection part
GO:044461	3.10E-02	7	bacterial-type flagellum part
GO:044460	3.10E-02	7	flagellum part
GO:030694	4.73E-02	3	bacterial-type flagellum basal body, rod

### Effect of RBL67 to the virulence response of N-15

GO enrichment analysis revealed enriched differential expression of some virulence genes, such as the 42 genes belonging to “protein secretion by the type III secretion system” (GO:030254) in the co-culture. Therefore we analyzed the regulation of all putative virulence factors of *Salmonella* LT2. A comparison to the virulence database VFDB revealed 151 genes in LT2 putatively involved in virulence [39]. Of these 151 genes, one was higher expressed in mono-culture, i.e. *phoP* encoding the transcriptional regulator PhoP, a member of the two-component system PhoQ-PhoP. The PhoQ encoding gene was also overexpressed in the mono-culture, although not significant (FDR = 0.063, Additional file 1: Table S2).

In the co-culture, 122 virulence genes were higher expressed, a significant enrichment of expressed virulence genes (*p* = 7 × 10^−39^ in Fisher’s test). The large majority of genes were involved in secretion systems (*N*  = 66) and fimbrial adherence determinants (*N* = 51). The pathogeny island 1 (SPI-1) encodes 39 genes [39] of which 30 were significantly higher expressed in the co-culture including the complete type III secretion system 1 (TTSS-1) consisting of *sipB*, *sipD*, *prgIHK*, *invACBGH*, *spaSRQPO*, and *sicAP*. Only *avrAI*, *sprB*, *hilC*, *orgC* and *hilD* were not higher expressed in co-culture. Additionally genes located on SPI-2 were higher expressed in co-culture, including the TTSS-2 genes *ssrAB*, s*saBCDE*, s*saGHIJKLMVNOPQRSTU*, *sseAB*, *sseCDE*, *sseFG*, *sscA*, and *sscB*. Further the main activation complex of type 1 fimbriae *fimY*, *fimW* and *fimZ* where higher expressed in co-culture, albeit the latter not significant.

## Discussion

Antagonism and protective effects of selected *B. thermophilum* strains against enterobacteriaceae have been observed in several studies [7–9, 13, 14], but the underlying mechanisms of this antagonism are unknown. In this study we used RNA-sequencing to investigate the global transcriptional response of RBL67 and *Salmonella* N-15 in mono- and co-culture. To our knowledge we present the first study investigating the interaction of a probiotic *Bifidobacterium* strain with enteropathogenic *S.* Typhimurium using RNA-sequencing.

RNA-sequencing was previously shown to be a powerful method to investigate genome-wide transcript analysis in mixed-culture experiments [35]. In our study we could map at least 10 million reads specifically to one of the genomes, which is clearly above the 5 million reads needed for differential expression analyses in bacterial genomes [41]. The transcriptome of N-15 mapped to the genome *of Salmonella* Typhimurium LT2 had a similar efficiency as the mapping of the transcriptome of RBL67 to the RBL67 genome, suggesting that mapping reads to a closely related genome is possible. The pathogenicity islands of *Salmonella* Typhimurium strains are conserved and difference in virulence factors contents mainly occurs on plasmid [42]. We could map RNAseq reads against the plasmid of LT-2, indicating that N-15 has a virulence-genes-encoding plasmid similar to that of LT2. Hence, both strains seem highly similar and the RNAseq data presented resemble closely the transcriptome profile of *Salmonella* Typhimurium strain N-15. Sampling points were chosen when growth speed, cell number and metabolite concentrations were similar in both cultures to allow accurate transcriptomic profiling. Further, fermentations were performed under pH controlled condition to exclude low pH effects.

RBL67 growth was slightly but significantly enhanced in presence of *Salmonella* N-15. Also growth of other *Bifidobacterium* species (*B. globosum*, *B. animalis*, *B. breve*) was shown to be stimulated by *S.* Typhimurium and *S.* Enteriditis, albeit under pH uncontrolled conditions [43]. A glutamate producing enzyme was repressed and a glutamate consuming enzyme activated in RBL67 in co-culture, suggesting a change in glutamate availability in the presence of N-15. Interestingly, *Salmonella* accumulates glutamate under various conditions [44] and lysing *Salmonella* cells could provide *B. thermophilum* with additional glutamate resulting in the change in amino acid metabolism and possibly also in the observed growth rate. The elevated expression of 3 stress genes suggests that RBL67 is exposed to weak stress in the presence of *Salmonella* N-15, but the enhanced growth performance indicates that the microbe was able to cope with the stress in the co-culture.

The *Salmonella* N-15 transcriptome was clearly affected by presence of RBL67. Many virulence genes were higher expressed in *Salmonella* N-15 during co-culture with RBL67 and such increased expression may enhance infection rate. However, this would contradict with previous results showing reduced invasion capacity of *Salmonella* to HT29-MTX cells in presence of RBL67 [13]. *Salmonella* virulence is tightly controlled and the activity of virulence factors at the right time, correct place and in appropriate amounts is crucial for virulence [45]. Further, environmental factors such as acetate can trigger virulence gene expression in *Salmonella* [20]. A low concentration of 15 mM acetate at pH 6.7 induces the three invasion determinants *hilA*, *invF* and *sipC* in *S.* Typhimurium and the induction is dependent on acetate kinase (*ackA*) and phosphotransacetylase (*pta*) activity [46]. The genes *hilA*, and *invF* were higher expressed in co-culture but *ackA* and *pta* were down regulated in co-cultures (Additional file 1: Table S2) and therefore *hilA* was likely not activated by acetate. The two-component system PhoQ-PhoP was down-regulated in the co-culture. PhoQ-PhoP is a repressor of *hilA*, a key regulator for *Salmonella* invasion [18, 21] and the higher expression of *hilA* observed in the co-culture seems therefore due to a repressor release mediated by PhoQ-PhoP.

Invasion of *Salmonella* follows sequential expression of first flagellar genes, followed by genes encoded on SPI-1, and eventually type 1 fimbrial genes [47]. Flagellar genes were repressed while genes of SPI-1 and type 1 fimbriae genes were activated in co-culture compared to mono-culture. This shows that N-15 in co-culture is further progressed in the sequential expression for infection and the balance in virulence gene expression is disturbed by the presence of RBL67. In fact, the expression of SPI-1 and SPI-2 and repression of flagellar genes observed in the co-culture resembles the transcriptional profile of *Salmonella* cells in fibroblast after infection [48]. Further, an early activation of the type III secretion system-1 (TTSS-1) located on SPI-1 was observed in co-culture. A TTSS-1 expressing *S.* Typhimurium subpopulation is essential for infection, but this subpopulation is also vulnerable to overgrowth by the non-TTSS-1 expressing subpopulation [49]. An imbalance in the regulation of TTSS-1 results in an inappropriate fraction of TTSS-1 expressing cells and eventually to a decreased infection rate [50]. This results *in situ* in reduced invasion of human intestinal cells and ultimately eliminates *Salmonella* from the lumen [50]. In vitro, a reduced infection of human intestinal cells by *Salmonella* in presence of RBL67 and repression of *Salmonella* by RBL67 in a continuous intestinal fermentation model was reported [13, 14].

Our data provide a first clue on a possible mechanism that could contributes to the antagonistic effects of RBL67 against *Salmonella* spec and other pathogens [7–9, 13–15]. The expression of virulence gene at early stage is a burden for the pathogen and may result in lower infection rate and subsequent wash-out from the lumen. In addition, the repression of flagellar genes reduces motility thereby preventing colonization of other areas. Whether the imbalance in virulence gene expression observed in vitro also occurs in situ remains to be elucidated. The effect may be reinforced by simultaneous protection by other probiotic mechanisms such as competition for adhesion sites and nutrients, and acetate production.

## Conclusion

Our study provides first insights into the transcriptome response of *B. thermophilum* RBL67 and *S.* Typhimurium grown in co-cultures under simplified conditions and reveals possible molecular mechanisms of probiotic-pathogen interaction. Our data show that RBL67 has a huge impact on the transcriptome of *Salmonella* and causes in an imbalanced virulence gene expression. This imbalance in the cascade pathway of virulence could represent a novel possible mechanism of how probiotic organisms can protect the host against infections.

### Availability of data and materials

Data presented in this study are available under NCBI BioProject Record PRJNA274782 accessible through http://www.ncbi.nlm.nih.gov/bioproject/PRJNA274782. Gene expression data are directly accessible through GEO Series accession number GSE65716 (http://www.ncbi.nlm.nih.gov/geo/query/acc.cgi?acc=GSE65716).

## Additional file

Additional file 1: Table S1.
*Salmonella* Typhimurium N-15 genes higher expressed in mono-culture. **Table S2:** *Salmonella* Typhimurium N-15 genes higher expressed in co-culture with RBL 67. (DOC 2173 kb)

## References

[1] Hill C, Guarner F, Reid G, Gibson GR, Merenstein DJ, Pot B, Morelli L, Canani RB, Flint HJ, Salminen S (2014). Expert consensus document. The International Scientific Association for Probiotics and Prebiotics consensus statement on the scope and appropriate use of the term probiotic. Nat Rev Gastroenterol Hepatol.

[2] O’Toole PW, Cooney JC (2008). Probiotic bacteria influence the composition and function of the intestinal microbiota. Interdiscip Perspect Infect Dis.

[3] Walsh CJ, Guinane CM, O’Toole PW, Cotter PD. Beneficial modulation of the gut microbiota. FEBS Lett. 2014;17;588(22):4120–30. 10.1016/j.febslet.2014.03.035 24681100

[4] Gaggia F, Mattarelli P, Biavati B (2010). Probiotics and prebiotics in animal feeding for safe food production. Int J Food Microbiol.

[5] Biavati B, Mattarelli P, Whitman WB, Kämpfer P, Goodfellow M, Garrity GM, Ludwig W (2009). Genus I. *Bifidobacterium*. Bergey’s manual of systematic bacteriology The Actinobacteria, vol. 5.

[6] Toure R, Kheadr E, Lacroix C, Moroni O, Fliss I (2003). Production of antibacterial substances by bifidobacterial isolates from infant stool active against *Listeria monocytogenes*. J Appl Microbiol.

[7] Sasaki T, Fukami S, Namioka S (1994). Enhanced resistance of mice to *Escherichia coli* infection induced by administration of peptidoglycan derived from *Bifidobacterium thermophilum*. J Vet Med Sci.

[8] Sasaki T, Fukami S, Namioka S (1994). Enhancement of cytotoxic activity of lymphocytes in mice by oral administration of peptidoglycan (PG) derived from *Bifidobacterium thermophilum*. J Vet Med Sci.

[9] Kobayashi C, Yokoyama H, Nguyen SV, Hashi T, Kuroki M, Kodama Y (2002). Enhancement of chicken resistance against *Escherichia coli* infection by oral administration of *Bifidobacterium thermophilum* preparations. Avian Dis.

[10] Jans C, Lacroix C, Follador R, Stevens MJ (2013). Complete genome sequence of the probiotic *Bifidobacterium thermophilum* strain RBL67. Genome Announc.

[11] von Ah U. Identification of Bifidobacterium thermophilum RBL67 isolated from baby feces and partial purification of its bacteriocin. PhD thesis. Zurich, Switzerland: ETH Zurich, Switzerland; 2006.

[12] von Ah U, Mozzetti V, Lacroix C, Kheadr EE, Fliss I, Meile L (2007). Classification of a moderately oxygen-tolerant isolate from baby faeces as *Bifidobacterium thermophilum*. BMC Microbiol.

[13] Zihler A, Gagnon M, Chassard C, Lacroix C (2011). Protective effect of probiotics on *Salmonella* infectivity assessed with combined *in vitro* gut fermentation-cellular models. BMC Microbiol.

[14] Zihler A, Le Blay G, Chassard C, Braegger C, Lacroix C. *Bifidobacterium thermophilum* RBL67 inhibits *S.* Typhimurium in an *in vitro* model of *Salmonella* infection in children. J Food Nutr Disord. 2014, in press.

[15] Gagnon M. Rôle des probiotiques lors d’infections entériques d’origine bactérienne et virale: analyses *in vitro* et études *in vivo* chez des modèles murines. PhD thesis. Québec: Université de Laval; 2007.

[16] Moroni O, Kheadr E, Boutin Y, Lacroix C, Fliss I (2006). Inactivation of adhesion and invasion of food-borne *Listeria monocytogenes* by bacteriocin-producing *Bifidobacterium* strains of human origin. Appl Environ Microbiol.

[17] Majowicz SE, Musto J, Scallan E, Angulo FJ, Kirk M, O’Brien SJ, Jones TF, Fazil A, Hoekstra RM, International Collaboration on Enteric Disease ‘Burden of Illness’ Studies (2010). The global burden of nontyphoidal Salmonella gastroenteritis. Clin Infect Dis.

[18] Fabrega A, Vila J (2013). *Salmonella enterica* serovar Typhimurium skills to succeed in the host: virulence and regulation. Clin Microbiol Rev.

[19] EFSA, ECDC (2014). The European Union summary report on trends and sources of zoonoses, zoonotic agents and food-borne outbreaks in 2012. EFSA Journal.

[20] Altier C (2005). Genetic and environmental control of *Salmonella* invasion. J Microbiol.

[21] Ellermeier JR, Slauch JM (2007). Adaptation to the host environment: regulation of the SPI1 type III secretion system in *Salmonella enterica* serovar Typhimurium. Curr Opin Microbiol.

[22] Lucas RL, Lee CA (2000). Unravelling the mysteries of virulence gene regulation in *Salmonella typhimurium*. Mol Microbiol.

[23] de Keersmaecker SC, Marchal K, Verhoeven TL, Engelen K, Vanderleyden J, Detweiler CS (2005). Microarray analysis and motif detection reveal new targets of the *Salmonella enterica* serovar Typhimurium HilA regulatory protein, including hilA itself. J Bacteriol.

[24] Gong J, Yang CB (2012). Advances in the methods for studying gut microbiota and their relevance to the research of dietary fiber functions. Food Res Int.

[25] Duncan SH, Hold GL, Barcenilla A, Stewart CS, Flint HJ (2002). *Roseburia intestinalis* sp. nov., a novel saccharolytic, butyrate-producing bacterium from human faeces. Int J Syst Evol Microbiol.

[26] Tanner SA, Zihler Berner A, Rigozzi E, Grattepanche F, Chassard C, Lacroix C (2014). *In vitro* continuous fermentation model (PolyFermS) of the swine proximal colon for simultaneous testing on the same gut microbiota. PLoS One.

[27] Rada V, Sirotek K, Petr J (1999). Evaluation of selective media for bifidobacteria in poultry and rabbit caecal samples. J Veterinary Med Ser B.

[28] Stevens MJ, Wiersma A, de Vos WM, Kuipers OP, Smid EJ, Molenaar D, Kleerebezem M (2008). Improvement of *Lactobacillus plantarum* aerobic growth as directed by comprehensive transcriptome analysis. Appl Environ Microbiol.

[29] Winkler ME (1979). Ribosomal ribonucleic acid isolated from Salmonella typhimurium: absence of the intact 23S species. J Bacteriol.

[30] Smith NH, Crichton PB, Old DC, Higgins CF (1988). Ribosomal-RNA patterns of *Escherichia coli*, *Salmonella typhimurium* and related *Enterobacteriaceae*. J Med Microbiol.

[31] McCarthy DJ, Chen Y, Smyth GK (2012). Differential expression analysis of multifactor RNA-Seq experiments with respect to biological variation. Nucleic Acids Res.

[32] Robinson MD, Smyth GK (2007). Moderated statistical tests for assessing differences in tag abundance. Bioinformatics.

[33] Robinson MD, Smyth GK (2008). Small-sample estimation of negative binomial dispersion, with applications to SAGE data. Biostatistics.

[34] Robinson MD, McCarthy DJ, Smyth GK (2010). edgeR: a Bioconductor package for differential expression analysis of digital gene expression data. Bioinformatics.

[35] Rosenthal AZ, Matson EG, Eldar A, Leadbetter JR (2011). RNA-seq reveals cooperative metabolic interactions between two termite-gut spirochete species in co-culture. ISME J.

[36] Conesa A, Gotz S, Garcia-Gomez JM, Terol J, Talon M, Robles M (2005). Blast2GO: a universal tool for annotation, visualization and analysis in functional genomics research. Bioinformatics.

[37] Maere S, Heymans K, Kuiper M (2005). BiNGO: a Cytoscape plugin to assess overrepresentation of gene ontology categories in biological networks. Bioinformatics.

[38] Shannon P, Markiel A, Ozier O, Baliga NS, Wang JT, Ramage D, Amin N, Schwikowski B, Ideker T (2003). Cytoscape: a software environment for integrated models of biomolecular interaction networks. Genome Res.

[39] Chen L, Xiong Z, Sun L, Yang J, Jin Q (2012). VFDB 2012 update: toward the genetic diversity and molecular evolution of bacterial virulence factors. Nucleic Acids Res.

[40] Edgar R, Domrachev M, Lash AE (2002). Gene Expression Omnibus: NCBI gene expression and hybridization array data repository. Nucleic Acids Res.

[41] next-generation-sequencing-guide (2015). Available at https://genohub.com/next-generation-sequencing-guide/. Accessed 15 Oct 2015.

[42] Dhanani AS, Block G, Dewar K, Forgetta V, Topp E, Beiko RG, Diarra MS (2015). Genomic comparison of non-typhoidal Salmonella enterica Serovars Typhimurium, Enteritidis, Heidelberg, Hadar and Kentucky isolates from broiler chickens. PLoS One.

[43] Bielecka M, Biedrzycka E, Smoragiewicz W, Smieszek M (1998). Interaction of *Bifidobacterium* and *Salmonella* during associated growth. Int J Food Microbiol.

[44] Yan D, Ikeda TP, Shauger AE, Kustu S (1996). Glutamate is required to maintain the steady-state potassium pool in Salmonella typhimurium. Proc Natl Acad Sci U S A.

[45] Kato A, Groisman EA, Utsumi R (2008). The PhoQ/PhoP regulatory network of *Salmonella enterica*. Bacterial signal transduction: networks and drug targets.

[46] Lawhon SD, Maurer R, Suyemoto M, Altier C (2002). Intestinal short-chain fatty acids alter *Salmonella typhimurium* invasion gene expression and virulence through BarA/SirA. Mol Microbiol.

[47] Saini S, Slauch JM, Aldridge PD, Rao CV (2010). Role of cross talk in regulating the dynamic expression of the flagellar *Salmonella* pathogenicity island 1 and type 1 fimbrial genes. J Bacteriol.

[48] Nunez-Hernandez C, Tierrez A, Ortega AD, Pucciarelli MG, Godoy M, Eisman B, Casadesus J, Garcia-del Portillo F (2013). Genome expression analysis of nonproliferating intracellular Salmonella enterica serovar Typhimurium unravels an acid pH-dependent PhoP-PhoQ response essential for dormancy. Infect Immun.

[49] Sturm A, Heinemann M, Arnoldini M, Benecke A, Ackermann M, Benz M, Dormann J, Hardt WD (2011). The cost of virulence: retarded growth of *Salmonella* Typhimurium cells expressing type III secretion system 1. PLoS Pathog.

[50] Diard M, Garcia V, Maier L, Remus-Emsermann MN, Regoes RR, Ackermann M, Hardt WD (2013). Stabilization of cooperative virulence by the expression of an avirulent phenotype. Nature.

