# Supplementary material for: Bifidobacterium thermophilum RBL67 impacts on growth and virulence gene expression of Salmonella enterica subsp. enterica serovar Typhimurium
Source: BMC Microbiol. 2016 Mar 18;16:46. doi: 10.1186/s12866-016-0659-x (PMC4797131; doi:10.1186/s12866-016-0659-x)
Supplement: Additional file 1: Table S1. — Salmonella Typhimurium N-15 genes higher expressed in mono-culture. Table S2: Salmonella Typhimurium N-15 genes higher expressed in co-culture with RBL 67. (DOC 2173 kb) [file 12866_2016_659_MOESM1_ESM.doc]

Table S1: *Salmonella* Typhimurium N-15 genes higher expressed in mono-culture.

| **ORF** | **Gene** | **Function** | **logFC** | **logCPM** | **FDR** |
| --- | --- | --- | --- | --- | --- |
| STM0004 | thrC | threonine synthase | 1.30 | 7.25 | 1.32E-02 |
| STM0005 | yaaA | hypothetical protein | 1.57 | 7.34 | 5.00E-03 |
| STM0007 | talB | transaldolase B | 2.49 | 9.46 | 4.94E-03 |
| STM0008 | mogA | molybdenum cofactor biosynthesis protein MogA | 1.12 | 6.14 | 9.07E-03 |
| STM0043 | rpsT | 30S ribosomal protein S20 | 2.63 | 10.23 | 1.12E-02 |
| STM0045 | ribF | bifunctional riboflavin kinase/FMNadenylyltransferase | 1.82 | 7.42 | 1.57E-03 |
| STM0046 | ileS | isoleucyl-tRNA synthetase | 1.73 | 10.24 | 3.68E-02 |
| STM0047 | lspA | lipoprotein signal peptidase | 1.59 | 7.42 | 1.10E-02 |
| STM0048 | slpA | FKBP-type peptidyl-prolyl cis-trans isomerase | 1.60 | 6.41 | 1.95E-04 |
| STM0049 | ispH | 4-hydroxy-3-methylbu t-2-enyl diphosphatereductase | 1.25 | 6.89 | 6.29E-03 |
| STM0051 | rihC | ribonucleoside hydrolase RihC | 1.03 | 6.81 | 2.47E-02 |
| STM0064 | dapB | dihydrodipicolinate reductase | 1.13 | 6.44 | 8.57E-03 |
| STM0092 | surA | peptidyl-prolyl cis-trans isomerase SurA | 1.64 | 8.28 | 2.28E-02 |
| STM0095 | rluA | 23S rRNA/tRNA pseudouridine synthase A | 1.21 | 6.05 | 2.02E-03 |
| STM0118 | fruR | DNA-binding transcriptional regulator FruR | 1.49 | 7.58 | 2.91E-02 |
| STM0121 | ftsL | cell division protein FtsL | 1.21 | 6.66 | 1.48E-02 |
| STM0124 | murF | UDP-N-acetylmuramoyl -tripeptide--D-alanyl-D-alanin e ligase | 1.44 | 7.74 | 3.88E-02 |
| STM0128 | murG | undecaprenyldiphosph o-muramoylpentapeptidebeta-N- acetylglucosaminyltransferase | 1.18 | 7.26 | 2.06E-02 |
| STM0133 | ftsZ | cell division protein FtsZ | 2.17 | 9.38 | 8.02E-03 |
| STM0134 | lpxC | UDP-3-O-[3-hydroxymy ristoyl] N-acetylglucosaminedeacetylase | 2.35 | 9.64 | 8.35E-03 |
| STM0135 | yacA | SecA regulator SecM | 1.15 | 6.62 | 1.99E-02 |
| STM0139 | yacF | hypothetical protein | 1.29 | 6.79 | 5.42E-03 |
| STM0151 | pdhR | transcriptional regulator PdhR | 1.64 | 6.95 | 2.30E-03 |
| STM0153 | aceF | dihydrolipoamide acetyltransferase | 1.52 | 8.40 | 3.54E-02 |
| STM0154 | lpdA | dihydrolipoamide dehydrogenase | 1.54 | 8.93 | 4.41E-02 |
| STM0164 | STM0164 | transcriptional regulator | 1.61 | 6.97 | 8.32E-03 |
| STM0170 | hpt | hypoxanthine-guanine phosphoribosyltransferase | 1.57 | 6.88 | 2.21E-03 |
| STM0171 | yadF | carbonic anhydrase | 1.04 | 6.83 | 2.37E-02 |
| STM0180 | panD | aspartate alpha-decarboxylase | 1.96 | 6.83 | 1.73E-04 |
| STM0202 | hemL | glutamate-1-semialde hyde aminotransferase | 1.53 | 7.94 | 3.92E-02 |
| STM0204.S | yadR | iron-sulfur cluster insertion protein ErpA | 1.92 | 7.27 | 3.46E-03 |
| STM0211 | yaeH | hypothetical protein | 1.10 | 5.19 | 4.10E-02 |
| STM0213 | dapD | 2,3,4,5-tetrahydropy ridine-2,6-dicarboxylateN-succ inyltransferase | 2.04 | 8.28 | 1.04E-02 |
| STM0219 | frr | ribosome recycling factor | 1.88 | 8.79 | 2.98E-02 |
| STM0220 | dxr | 1-deoxy-D-xylulose 5-phosphate reductoisomerase | 1.51 | 7.11 | 6.19E-03 |
| STM0225 | hlpA | outer membrane protein OmpH | 1.73 | 9.20 | 4.83E-02 |
| STM0227 | fabZ | (3R)-hydroxymyristoy l-ACP dehydratase | 2.23 | 7.18 | 1.19E-04 |
| STM0228 | lpxA | UDP-N-acetylglucosam ine acyltransferase | 1.67 | 8.02 | 2.07E-02 |
| STM0232 | accA | acetyl-CoA carboxylase carboxyltransferasesubunit alpha | 1.94 | 8.33 | 1.01E-02 |
| STM0237 | rof | Rho-binding antiterminator | 1.36 | 6.46 | 5.83E-03 |
| STM0238 | yaeP | hypothetical protein | 2.21 | 5.79 | 1.05E-03 |
| STM0240 | yaeJ | peptidyl-tRNA hydrolase domain-containingprotein | 1.08 | 4.80 | 1.35E-02 |
| STM0243 | yaeB | regulatory protein | 1.11 | 6.05 | 3.88E-03 |
| STM0244 | rcsF | outer membrane lipoprotein | 1.01 | 6.54 | 3.13E-02 |
| STM0245 | metQ | DL-methionine transporter substrate-bindingsubunit | 1.33 | 7.41 | 2.72E-02 |
| STM0258 | yafD | hypothetical protein | 1.49 | 6.91 | 1.14E-02 |
| STM0310 | gmhA | phosphoheptose isomerase | 1.19 | 6.56 | 8.15E-03 |
| STM0312 | yafK | hypothetical protein | 1.15 | 6.93 | 4.76E-02 |
| STM0316 | pepD | aminoacyl-histidine dipeptidase | 1.82 | 9.62 | 3.01E-02 |
| STM0327 | STM0327 | hypothetical protein | 1.54 | 6.07 | 1.24E-03 |
| STM0386 | proC | pyrroline-5-carboxyl ate reductase | 1.69 | 6.91 | 1.38E-03 |
| STM0397 | phoB | transcriptional regulator PhoB | 1.11 | 6.78 | 3.16E-02 |
| STM0406 | yajC | preprotein translocase subunit YajC | 2.20 | 7.76 | 1.69E-03 |
| STM0408 | secF | preprotein translocase subunit SecF | 1.55 | 7.97 | 3.93E-02 |
| STM0415 | nrdR | transcriptional regulator NrdR | 1.63 | 6.73 | 6.56E-04 |
| STM0416 | ribD | bifunctionaldiaminoh ydroxyphosphoribosylaminopyrim idinedeaminase/5-amino-6-(5-ph osphoribosylamino)uracilreduct ase | 1.72 | 7.50 | 4.75E-03 |
| STM0418 | nusB | transcription antitermination protein NusB | 1.45 | 7.02 | 1.71E-02 |
| STM0423 | ispA | geranyltranstransfer ase | 1.22 | 7.12 | 3.45E-02 |
| STM0424 | xseB | exodeoxyribonuclease VII small subunit | 1.82 | 4.86 | 2.37E-04 |
| STM0434 | apbA | 2-dehydropantoate 2-reductase | 1.34 | 6.83 | 1.22E-02 |
| STM0435 | yajQ | nucleotide-binding protein | 1.78 | 8.21 | 2.80E-02 |
| STM0448 | clpP | ATP-dependent Clp protease proteolytic subunit | 1.72 | 7.62 | 1.76E-02 |
| STM0449 | clpX | ATP-dependent protease ATP-binding subunit ClpX | 2.39 | 9.27 | 3.17E-03 |
| STM0450 | lon | DNA-binding ATP-dependent protease La | 1.79 | 9.47 | 2.35E-02 |
| STM0451 | hupB | transcriptional regulator HU subunit beta | 3.26 | 9.54 | 5.80E-04 |
| STM0462 | glnK | nitrogen regulatory protein P-II 2 | 1.09 | 4.86 | 1.94E-02 |
| STM0474 | ybaJ | hypothetical protein | 1.70 | 6.42 | 5.34E-04 |
| STM0475 | acrB | acridine efflux pump | 1.61 | 9.83 | 4.41E-02 |
| STM0477 | acrR | DNA-binding transcriptional repressor AcrR | 1.13 | 6.00 | 1.02E-02 |
| STM0483 | apt | adenine phosphoribosyltransferase | 1.49 | 6.28 | 5.70E-03 |
| STM0484 | dnaX | DNA polymerase III subunits gamma and tau | 1.36 | 7.38 | 1.17E-02 |
| STM0485 | ybaB | hypothetical protein | 2.32 | 7.07 | 2.06E-04 |
| STM0486 | recR | recombination protein RecR | 2.29 | 7.25 | 2.42E-04 |
| STM0487.S | htpG | heat shock protein 90 | 1.78 | 10.02 | 3.60E-02 |
| STM0489 | hemH | ferrochelatase | 2.54 | 8.64 | 3.11E-03 |
| STM0495 | ybaK | hypothetical protein | 1.38 | 5.86 | 5.25E-04 |
| STM0496 | ybaP | hypothetical protein | 1.11 | 5.65 | 6.03E-03 |
| STM0499 | cueR | DNA-binding transcriptional regulator CueR | 1.34 | 6.26 | 1.68E-03 |
| STM0500 | ybbJ | hypothetical protein | 1.41 | 6.00 | 3.44E-03 |
| STM05020 | STM05020 | hypothetical protein | 2.30 | 5.84 | 8.77E-07 |
| STM0504 | ybbN | thioredoxin protein | 1.67 | 6.85 | 1.03E-03 |
| STM0516 | allR | DNA-binding transcriptional repressor AllR | 1.07 | 6.26 | 1.60E-02 |
| STM05225 | STM05225 | hypothetical protein | 2.44 | 4.22 | 5.59E-04 |
| STM0534 | purE | phosphoribosylaminoi midazole carboxylasecatalytic subunit | 1.28 | 5.22 | 2.91E-02 |
| STM0541 | ybcJ | hypothetical protein | 2.20 | 5.60 | 1.22E-06 |
| STM0542 | folD | bifunctional 5,10-methylene-tetrahydrofolat edehydrogenase/ 5,10-methylene-tetrahydrofolat ecyclohydrolase | 1.53 | 7.42 | 1.48E-02 |
| STM05615 | STM05615 | hypothetical protein | 1.23 | 4.81 | 5.14E-03 |
| STM0578 | nfnB | nitroreductase | 1.52 | 6.94 | 5.13E-03 |
| STM0608 | ahpC | alkyl hydroperoxide reductase subunit C | 2.30 | 10.71 | 4.01E-02 |
| STM0609 | ahpF | alkyl hydroperoxide reductase F52a subunit | 1.65 | 8.81 | 3.16E-02 |
| STM0614 | ybdQ | universal stress protein | 1.93 | 8.50 | 3.47E-02 |
| STM0616 | rnk | nucleoside diphosphate kinase regulator | 1.32 | 5.71 | 2.84E-03 |
| STM0617 | rna | ribonuclease I | 1.51 | 7.17 | 8.48E-03 |
| STM0630 | ccrB | camphor resistance protein CrcB | 1.16 | 5.69 | 5.71E-03 |
| STM0632 | tatE | twin arginine translocase protein E | 2.18 | 7.09 | 6.31E-04 |
| STM0633 | lipA | lipoyl synthase | 1.74 | 8.15 | 3.21E-02 |
| STM0635.S | lipB | lipoate-protein ligase B | 1.16 | 6.51 | 1.23E-02 |
| STM0636 | ybeD | hypothetical protein | 1.55 | 7.19 | 9.63E-03 |
| STM0637 | dacA | D-alanyl-D-alanine carboxypeptidase | 1.30 | 7.12 | 2.24E-02 |
| STM0642 | ybeB | hypothetical protein | 1.69 | 5.39 | 2.48E-04 |
| STM0646 | holA | DNA polymerase III subunit delta | 1.67 | 7.10 | 3.05E-03 |
| STM0647 | rlpB | LPS-assembly lipoprotein RlpB | 1.57 | 7.11 | 7.98E-03 |
| STM0667 | ybeX | transporter | 1.49 | 7.56 | 3.31E-02 |
| STM0668 | ybeY | metalloprotease | 1.50 | 6.21 | 1.81E-03 |
| STM0669 | phoL | phosphate starvation-inducible protein | 1.42 | 7.40 | 3.08E-02 |
| STM0680 | asnB | asparagine synthetase B | 1.71 | 7.71 | 1.57E-02 |
| STM0681 | nagD | UMP phosphatase | 1.12 | 6.25 | 4.38E-03 |
| STM0694 | fldA | flavodoxin FldA | 1.71 | 7.59 | 2.64E-02 |
| STM0695 | ybfE | LexA regulated protein | 1.68 | 5.31 | 2.19E-03 |
| STM0708 | ybfA | hypothetical protein | 2.21 | 5.15 | 2.68E-06 |
| STM0712 | ybgJ | carboxylase | 1.28 | 5.85 | 1.48E-02 |
| STM0713 | ybgK | carboxylase | 1.24 | 6.82 | 2.89E-02 |
| STM0731 | STM0731 | inner membrane protein | 1.73 | 6.10 | 3.48E-03 |
| STM0737 | sucB | dihydrolipoamide succinyltransferase | 1.30 | 7.14 | 1.44E-02 |
| STM0740 | cydA | cytochrome d terminal oxidase polypeptidesubunit I | 2.04 | 9.69 | 1.62E-02 |
| STM0741 | cydB | cytochrome d terminal oxidase polypeptidesubunit II | 1.96 | 8.86 | 1.23E-02 |
| STM0742 | ybgT | outer membrane lipoprotein | 2.48 | 6.01 | 9.77E-04 |
| STM0744 | ybgC | acyl-CoA thioester hydrolase YbgC | 1.67 | 6.58 | 3.23E-04 |
| STM0745 | tolQ | colicin uptake protein TolQ | 1.31 | 7.17 | 3.84E-02 |
| STM0746 | tolR | colicin uptake protein TolR | 1.26 | 6.19 | 9.73E-03 |
| STM0748 | tolB | translocation protein TolB | 2.64 | 9.24 | 8.52E-04 |
| STM0749 | pal | peptidoglycan-associ ated outer membranelipoprotein | 2.34 | 10.25 | 2.93E-02 |
| STM0750 | ybgF | tol-pal system protein YbgF | 2.13 | 7.94 | 9.72E-03 |
| STM0760 | aroG | phospho-2-dehydro-3- deoxyheptonate aldolase | 1.95 | 7.29 | 1.73E-03 |
| STM0774 | galK | galactokinase | 1.65 | 6.96 | 1.60E-03 |
| STM0780 | STM0780 | hypothetical protein | 2.23 | 7.46 | 7.38E-03 |
| STM0781 | modA | molybdate transporter periplasmic protein | 1.85 | 7.45 | 8.40E-03 |
| STM0782 | modB | molybdate ABC transporter permease | 1.38 | 6.24 | 1.91E-03 |
| STM0783 | modC | molybdate transporter ATP-binding protein | 1.47 | 6.61 | 1.38E-03 |
| STM0786 | ybhC | pectinesterase | 1.42 | 7.83 | 4.56E-02 |
| STM0802 | moaA | molybdenum cofactor biosynthesis protein A | 1.27 | 7.03 | 1.54E-02 |
| STM0803 | moaB | molybdopterin biosynthetic protein B | 1.06 | 6.82 | 4.57E-02 |
| STM0804 | moaC | molybdenum cofactor biosynthesis protein MoaC | 1.59 | 6.64 | 3.33E-03 |
| STM0805 | moaD | molybdopterin synthase small subunit | 1.56 | 5.21 | 3.70E-04 |
| STM0806 | moaE | molybdopterin guanine dinucleotide biosynthesisprotein MoaE | 1.74 | 6.00 | 7.45E-05 |
| STM0807 | ybhL | permease | 1.72 | 7.90 | 1.70E-02 |
| STM0822 | ybiB | glycosyl transferase family protein | 1.01 | 5.69 | 2.12E-02 |
| STM0823 | ybiJ | hypothetical protein | 1.72 | 4.85 | 6.67E-04 |
| STM0846 | moeA | molybdopterin biosynthesis protein MoeA | 1.21 | 6.65 | 6.15E-03 |
| STM0847 | iaaA | isoaspartyl peptidase | 1.09 | 6.14 | 4.07E-02 |
| STM0872 | grxA | glutaredoxin | 1.86 | 7.85 | 1.51E-02 |
| STM0888 | artM | arginine ABC transporter permease ArtM | 1.43 | 5.97 | 1.19E-03 |
| STM0889 | artQ | arginine ABC transporter permease ArtQ | 1.58 | 6.19 | 2.91E-04 |
| STM0930 | orfB | hypothetical protein | 1.40 | 5.33 | 1.80E-03 |
| STM0931 | ybjR | aminidase | 1.59 | 6.45 | 4.58E-04 |
| STM0934 | ltaA | L-threonine aldolase | 1.75 | 6.21 | 1.17E-03 |
| STM0943 | cspD | stress response protein | 3.31 | 6.09 | 9.45E-11 |
| STM0945 | clpA | ATP-dependent Clp protease ATP-binding subunit | 1.90 | 9.38 | 2.25E-02 |
| STM0953 | infA | translation initiation factor IF-1 | 2.26 | 8.26 | 1.04E-02 |
| STM0959 | lrp | leucine-responsive transcriptional regulator | 1.71 | 7.33 | 1.98E-02 |
| STM0960 | ftsK | DNA translocase FtsK | 1.42 | 8.66 | 4.41E-02 |
| STM0962 | ycaJ | recombination factor protein RarA | 1.38 | 7.05 | 6.10E-03 |
| STM0963 | serS | seryl-tRNA synthetase | 2.42 | 9.38 | 3.95E-03 |
| STM0970 | pflA | pyruvate formate lyase-activating enzyme 1 | 1.53 | 7.32 | 2.12E-02 |
| STM0974 | focA | formate transporter | 1.75 | 8.75 | 4.17E-02 |
| STM0977 | serC | phosphoserine aminotransferase | 2.10 | 8.30 | 1.08E-02 |
| STM0980 | cmk | cytidylate kinase | 1.88 | 7.43 | 8.40E-03 |
| STM0982 | ihfB | integration host factor subunit beta | 1.88 | 7.15 | 9.72E-03 |
| STM0987 | ycaR | hypothetical protein | 2.23 | 5.99 | 2.10E-05 |
| STM0988 | kdsB | 3-deoxy-manno-octulo sonate cytidylyltransferase | 1.67 | 7.32 | 3.52E-03 |
| STM0991 | smtA | metallothionein SmtA | 1.08 | 6.29 | 7.91E-03 |
| STM0992 | mukF | condesin subunit F | 1.16 | 6.77 | 9.72E-03 |
| STM0993 | mukE | condesin subunit E | 1.26 | 6.15 | 1.92E-03 |
| STM0995 | ycbB | murein L,D-transpeptidase | 1.75 | 7.68 | 1.72E-02 |
| STM0997 | ycbL | metallo-beta-lactama se | 1.53 | 7.20 | 3.45E-03 |
| STM0998 | aspC | aromatic amino acid aminotransferase | 1.86 | 8.52 | 2.34E-02 |
| STM1004 | pncB | nicotinate phosphoribosyltransferase | 1.02 | 6.87 | 2.00E-02 |
| STM1005 | STM1005 | integrase | 1.17 | 7.43 | 4.45E-02 |
| STM1066 | rmf | ribosome modulation factor | 1.22 | 4.20 | 9.02E-03 |
| STM1067 | fabA | 3-hydroxydecanoyl-AC P dehydratase | 2.01 | 6.77 | 3.57E-04 |
| STM1069 | ycbG | hypothetical protein | 1.24 | 5.08 | 6.83E-03 |
| STM1076 | mgsA | methylglyoxal synthase | 1.50 | 5.61 | 4.03E-04 |
| STM1078 | STM1078 | hypothetical protein | 1.17 | 5.93 | 6.83E-03 |
| STM1079 | yccV | heat shock protein HspQ | 1.05 | 5.91 | 2.77E-02 |
| STM1111 | yccD | chaperone-modulator protein CbpM | 1.41 | 6.71 | 2.14E-02 |
| STM1113 | scsA | suppression of copper sensitivity protein A | 1.07 | 4.40 | 1.51E-02 |
| STM1119 | wraB | TrpR binding protein WrbA | 1.67 | 7.73 | 4.07E-02 |
| STM1123 | STM1123 | hypothetical protein | 1.53 | 5.19 | 2.18E-03 |
| STM1136 | ycdX | hydrolase | 1.47 | 6.24 | 6.89E-04 |
| STM1147 | STM1147 | hypothetical protein | 1.11 | 6.07 | 1.45E-02 |
| STM1155 | htrB | lipid A biosynthesis lauroyl acyltransferase | 1.18 | 6.25 | 2.53E-03 |
| STM1160 | solA | N-methyltryptophan oxidase | 1.69 | 6.21 | 6.25E-04 |
| STM1161.S | bssS | biofilm formation regulatory protein BssS | 1.59 | 5.69 | 1.38E-03 |
| STM1162 | dinI | DNA damage-inducible protein I | 2.16 | 5.26 | 1.47E-06 |
| STM1164 | yceB | hypothetical protein | 1.50 | 5.91 | 3.09E-03 |
| STM1165 | grxB | glutaredoxin | 1.54 | 6.89 | 9.29E-03 |
| STM1168 | yceH | hypothetical protein | 1.33 | 5.99 | 4.81E-03 |
| STM1169 | mviM | virulence protein | 1.08 | 6.01 | 2.13E-02 |
| STM1171 | flgN | FlgK/FlgL export chaperone | 2.27 | 7.48 | 3.68E-03 |
| STM1172 | flgM | anti-sigma-28 factor FlgM | 2.47 | 7.08 | 1.49E-03 |
| STM1173 | flgA | flagellar basal body P-ring biosynthesis proteinFlgA | 1.84 | 6.44 | 1.34E-04 |
| STM1174 | flgB | flagellar basal body rod protein FlgB | 1.80 | 6.07 | 4.29E-04 |
| STM1175 | flgC | flagellar basal body rod protein FlgC | 1.16 | 5.94 | 1.75E-02 |
| STM1179 | flgG | flagellar basal body rod protein FlgG | 2.00 | 7.60 | 7.88E-03 |
| STM1180 | flgH | flagellar basal body L-ring protein | 1.25 | 6.03 | 5.05E-03 |
| STM1181 | flgI | flagellar basal body P-ring protein | 1.18 | 6.86 | 1.32E-02 |
| STM1183 | flgK | flagellar hook-associated protein FlgK | 2.18 | 9.43 | 1.53E-02 |
| STM1184 | flgL | flagellar hook-associated protein FlgL | 2.54 | 8.94 | 4.18E-03 |
| STM1190 | yceD | hypothetical protein | 1.83 | 8.99 | 4.95E-02 |
| STM1191 | rpmF | 50S ribosomal protein L32 | 2.59 | 8.38 | 1.70E-02 |
| STM1192 | plsX | glycerol-3-phosphate acyltransferase PlsX | 1.24 | 7.13 | 3.59E-02 |
| STM1193 | fabH | 3-oxoacyl-ACP synthase | 1.70 | 7.67 | 1.79E-02 |
| STM1194 | fabD | acyl carrier protein S-malonyltransferase | 1.98 | 8.25 | 1.74E-02 |
| STM1195 | fabG | 3-ketoacyl-ACP reductase | 1.90 | 8.93 | 2.15E-02 |
| STM1197 | fabF | 3-oxoacyl-(acyl carrier protein) synthase II | 2.63 | 9.83 | 4.88E-03 |
| STM1200 | tmk | thymidylate kinase | 1.20 | 6.13 | 3.11E-03 |
| STM1201 | holB | DNA polymerase III subunit delta' | 1.41 | 6.01 | 3.23E-04 |
| STM1203 | ptsG | PTS system glucose-specific transporter subunitIIBC | 2.38 | 9.98 | 1.10E-02 |
| STM1206 | ycfL | outer membrane lipoprotein | 2.03 | 6.52 | 1.22E-04 |
| STM1210 | ycfP | hypothetical protein | 1.32 | 6.80 | 1.58E-02 |
| STM1211 | ndh | respiratory NADH dehydrogenase 2 | 1.49 | 7.79 | 2.83E-02 |
| STM1213 | ycfQ | transcriptional repressor | 1.73 | 6.28 | 3.77E-04 |
| STM1214 | ycfR | outer membrane protein | 2.41 | 5.63 | 2.37E-05 |
| STM1216 | mfd | transcription-repair coupling factor | 1.44 | 8.45 | 4.15E-02 |
| STM1218 | lolD | lipoprotein transporter ATP-binding subunit | 1.13 | 5.29 | 1.65E-02 |
| STM1220 | ycfX | N-acetyl-D-glucosami ne kinase | 1.29 | 6.30 | 1.09E-03 |
| STM1221 | cobB | NAD-dependent deacetylase | 1.72 | 6.70 | 9.25E-05 |
| STM1229 | ycfD | hypothetical protein | 1.38 | 6.68 | 4.09E-03 |
| STM1231 | phoP | DNA-binding transcriptional regulator PhoP | 2.13 | 7.72 | 8.29E-03 |
| STM1233 | ycfC | hypothetical protein | 1.70 | 6.20 | 1.39E-03 |
| STM1238 | icdA | isocitrate dehydrogenase | 1.12 | 7.27 | 4.39E-02 |
| STM1276 | STM1276 | hypothetical protein | 1.36 | 4.54 | 8.78E-03 |
| STM1277 | yeaO | hypothetical protein | 1.39 | 4.95 | 5.42E-03 |
| STM1280 | yeaL | inner membrane protein | 1.49 | 4.73 | 2.89E-03 |
| STM1289 | yeaD | aldose 1-epimerase | 1.65 | 8.08 | 3.56E-02 |
| STM1292 | yeaC | hypothetical protein | 1.67 | 4.82 | 3.26E-03 |
| STM1297 | selD | selenophosphate synthetase | 1.76 | 7.21 | 4.34E-03 |
| STM1300 | STM1300 | hypothetical protein | 2.24 | 5.78 | 2.93E-03 |
| STM1301 | STM1301 | pyrimidine (deoxy)nucleoside triphosphatepyrophosphohydrola se | 2.51 | 6.05 | 2.56E-05 |
| STM1302 | xthA | exonuclease III | 1.48 | 6.64 | 2.41E-03 |
| STM1310 | nadE | NAD synthetase | 1.57 | 6.51 | 2.51E-03 |
| STM1311 | osmE | DNA-binding transcriptional activator OsmE | 1.81 | 6.10 | 3.15E-03 |
| STM1322 | yniC | 2-deoxyglucose-6-pho sphatase | 1.57 | 6.05 | 3.11E-04 |
| STM1324 | STM1324 | hypothetical protein | 1.25 | 5.74 | 1.12E-02 |
| STM1326 | pfkB | 6-phosphofructokinas e | 1.62 | 6.94 | 2.89E-03 |
| STM1336 | rplT | 50S ribosomal protein L20 | 2.40 | 10.51 | 4.04E-02 |
| STM1339 | ihfA | integration host factor subunit alpha | 1.94 | 7.38 | 1.71E-02 |
| STM1367 | ydiH | hypothetical protein | 2.53 | 6.80 | 1.55E-05 |
| STM1368 | STM1368 | Na+-dicarboxylate symporter | 1.59 | 7.16 | 1.75E-03 |
| STM1377 | lpp | murein lipoprotein | 3.39 | 12.59 | 1.18E-02 |
| STM1389 | orf319 | inner membrane protein | 1.50 | 6.47 | 3.44E-03 |
| STM1426 | ribE | riboflavin synthase subunit alpha | 1.23 | 6.83 | 1.07E-02 |
| STM1433 | ydhD | hypothetical protein | 1.91 | 6.19 | 1.87E-03 |
| STM1434 | rnt | ribonuclease T | 1.51 | 6.44 | 2.42E-03 |
| STM1435 | gloA | glyoxalase I | 2.07 | 6.69 | 2.07E-04 |
| STM1448 | pdxH | pyridoxamine 5'-phosphate oxidase | 2.01 | 7.80 | 1.22E-02 |
| STM1451 | gst | glutathionine S-transferase | 1.72 | 6.64 | 2.58E-03 |
| STM1453 | nth | endonuclease III | 1.14 | 5.61 | 5.41E-03 |
| STM1455 | ydgP | electron transport complex protein RnfG | 1.05 | 5.14 | 3.73E-02 |
| STM1460 | ydgK | inner membrane protein | 1.65 | 6.48 | 1.90E-03 |
| STM1462.S | ydgJ | oxidoreductase | 1.30 | 7.24 | 2.31E-02 |
| STM1463 | add | adenosine deaminase | 1.83 | 6.70 | 5.05E-05 |
| STM1467 | manA | mannose-6-phosphate isomerase | 1.27 | 7.31 | 3.66E-02 |
| STM1479 | pntA | NAD(P) transhydrogenase subunit alpha | 1.92 | 8.48 | 1.46E-02 |
| STM1480 | pntB | pyridine nucleotide transhydrogenase | 2.10 | 7.84 | 3.02E-03 |
| STM1500 | ynfD | outer membrane protein | 1.06 | 4.64 | 2.60E-02 |
| STM1502 | speG | spermidine N1-acetyltransferase | 1.40 | 6.85 | 9.63E-03 |
| STM1503 | ynfB | hypothetical protein | 1.28 | 7.07 | 4.52E-02 |
| STM1509 | ydfZ | hypothetical protein | 3.38 | 8.38 | 4.25E-04 |
| STM1510 | ydfH | regulatory protein | 1.24 | 5.80 | 6.97E-03 |
| STM1518 | marB | hypothetical protein | 1.01 | 3.84 | 4.76E-02 |
| STM1524 | yneI | succinate semialdehyde dehydrogenase | 1.97 | 8.32 | 9.86E-03 |
| STM1525 | yneH | glutaminase | 1.31 | 5.90 | 6.77E-04 |
| STM1565 | rpsV | 30S ribosomal subunit S22 | 2.72 | 6.55 | 9.81E-05 |
| STM1567 | adhP | alcohol dehydrogenase | 1.12 | 6.96 | 4.27E-02 |
| STM1572 | nmpC | outer membrane porin precursor | 2.54 | 12.81 | 3.62E-02 |
| STM1575 | STM1575 | transcriptional regulator | 1.00 | 4.68 | 2.03E-02 |
| STM1592 | ydcY | hypothetical protein | 1.76 | 4.83 | 1.36E-03 |
| STM1642 | acpD | azoreductase | 1.09 | 5.83 | 4.96E-02 |
| STM1652 | ynaF | universal stress protein | 2.18 | 7.12 | 3.73E-04 |
| STM1658 | ydaL | hypothetical protein | 1.74 | 5.91 | 6.08E-04 |
| STM1660.S | fnr | fumarate/nitrate reduction transcriptionalregulator | 1.45 | 7.34 | 3.78E-02 |
| STM1661 | ydaA | universal stress protein UspE | 1.85 | 7.65 | 1.82E-02 |
| STM1662 | ynaJ | inner membrane protein | 1.01 | 3.50 | 3.01E-02 |
| STM1682 | tpx | thiol peroxidase | 1.74 | 6.14 | 2.07E-04 |
| STM1683 | tyrR | DNA-binding transcriptional regulator TyrR | 1.06 | 6.97 | 2.37E-02 |
| STM1686 | pspE | thiosulfate:cyanide sulfurtransferase | 1.40 | 4.83 | 2.72E-03 |
| STM1690 | pspA | phage shock protein PspA | 1.28 | 6.25 | 9.98E-03 |
| STM1700 | fabI | enoyl-(acyl carrier protein) reductase | 2.14 | 8.98 | 1.04E-02 |
| STM1705 | osmB | lipoprotein | 1.05 | 3.99 | 2.18E-02 |
| STM1707 | pyrF | orotidine 5'-phosphate decarboxylase | 1.13 | 5.84 | 2.85E-02 |
| STM1709 | yciS | inner membrane protein | 1.44 | 5.27 | 6.61E-03 |
| STM1711 | ribA | GTP cyclohydrolase II | 1.17 | 6.61 | 1.97E-02 |
| STM1713 | cysB | transcriptional regulator CysB | 1.53 | 6.33 | 1.79E-03 |
| STM1714 | topA | DNA topoisomerase I | 1.51 | 8.11 | 3.17E-02 |
| STM1715 | yciN | hypothetical protein | 2.41 | 5.85 | 6.25E-06 |
| STM1719 | yciL | 23S rRNA pseudouridylate synthase B | 1.20 | 6.14 | 6.02E-03 |
| STM1720 | yciO | hypothetical protein | 1.43 | 6.56 | 3.00E-03 |
| STM1734 | yciC | hypothetical protein | 1.56 | 6.26 | 5.60E-04 |
| STM1737 | tonB | transporter | 1.05 | 5.37 | 1.01E-02 |
| STM1738 | yciI | YciI-like protein | 2.20 | 5.25 | 2.60E-06 |
| STM1740 | STM1740 | dsDNA-mimic protein | 1.34 | 5.23 | 1.61E-02 |
| STM1746.S | oppA | oligopeptide transport protein | 1.86 | 7.48 | 1.83E-02 |
| STM1749 | adhE | bifunctional acetaldehyde-CoA/alcoholdehydr ogenase | 2.36 | 12.69 | 3.38E-02 |
| STM1753 | hnr | response regulator of RpoS | 1.04 | 5.97 | 1.05E-02 |
| STM1755 | ychJ | hypothetical protein | 1.20 | 5.83 | 5.64E-03 |
| STM1756 | purU | formyltetrahydrofola te deformylase | 1.13 | 6.64 | 2.60E-02 |
| STM1769 | ychN | sulfur reduction protein | 2.20 | 6.20 | 4.61E-05 |
| STM1772 | kdsA | 2-dehydro-3-deoxypho sphooctonate aldolase | 1.61 | 7.97 | 3.20E-02 |
| STM1775 | hemK | N5-glutamine S-adenosyl-L-methionine-depend entmethyltransferase | 1.20 | 5.71 | 9.29E-03 |
| STM1777 | hemA | glutamyl-tRNA reductase | 2.03 | 8.06 | 9.21E-03 |
| STM1778 | lolB | outer membrane lipoprotein LolB | 1.39 | 6.15 | 5.79E-03 |
| STM1779 | ipk | 4-diphosphocytidyl-2 -C-methyl-D-erythritolkinase | 3.01 | 7.73 | 5.45E-05 |
| STM1784 | ychF | GTP-dependent nucleic acid-binding protein EngD | 1.67 | 8.00 | 3.92E-02 |
| STM1790 | STM1790 | hydrogenase-1 operon protein HyaE | 1.79 | 7.46 | 1.93E-02 |
| STM1794 | STM1794 | membrane protein | 2.29 | 6.23 | 3.68E-03 |
| STM1801 | ycgO | potassium/proton antiporter | 1.33 | 6.82 | 6.40E-03 |
| STM1805 | fadR | fatty acid metabolism regulator | 1.06 | 6.18 | 8.45E-03 |
| STM1806 | nhaB | sodium/proton antiporter | 1.20 | 6.98 | 1.07E-02 |
| STM1807 | dsbB | disulfide bond formation protein B | 1.53 | 5.69 | 3.74E-04 |
| STM1808 | STM1808 | hypothetical protein | 1.06 | 4.49 | 2.64E-02 |
| STM1809 | STM1809 | hypothetical protein | 1.44 | 6.53 | 3.21E-02 |
| STM1814 | minC | septum formation inhibitor | 1.61 | 6.47 | 1.21E-03 |
| STM1815 | minD | cell division inhibitor MinD | 1.92 | 7.89 | 1.17E-02 |
| STM1816 | minE | cell division topological specificity factorMinE | 2.46 | 6.28 | 2.56E-06 |
| STM1822 | yoaB | translation initiation inhibitor | 1.32 | 5.89 | 7.48E-03 |
| STM1826 | sdaA | L-serine deaminase I/L-threonine deaminase I | 1.40 | 7.49 | 2.70E-02 |
| STM1830 | manX | PTS system mannose-specific transporter subunitIIAB | 2.33 | 9.86 | 1.21E-02 |
| STM1831 | manY | PTS system mannose-specific transporter subunitIIC | 2.95 | 9.52 | 7.13E-04 |
| STM1832 | manZ | PTS system mannose-specific transporter subunitIID | 2.79 | 9.38 | 2.61E-03 |
| STM1834 | yebN | hypothetical protein | 1.38 | 6.77 | 5.48E-03 |
| STM1837 | cspC | cold shock-like protein CspC | 1.94 | 7.40 | 1.43E-02 |
| STM1838 | yobF | hypothetical protein | 2.37 | 6.42 | 3.89E-04 |
| STM1844 | htpX | heat shock protein HtpX | 1.62 | 7.72 | 2.18E-02 |
| STM1847 | yebR | nucleotide-binding protein | 1.38 | 6.65 | 8.61E-03 |
| STM1873 | STM1873 | hypothetical protein | 1.09 | 5.09 | 1.57E-02 |
| STM1884 | eda | keto-hydroxyglutarat e-aldolase/keto-deoxy-phosphog luconate aldolase | 1.87 | 7.07 | 1.83E-03 |
| STM1886 | zwf | glucose-6-phosphate 1-dehydrogenase | 1.64 | 7.99 | 2.48E-02 |
| STM1888 | pykA | pyruvate kinase | 1.93 | 9.13 | 2.20E-02 |
| STM1893 | znuB | high-affinity zinc transporter membrane protein | 1.10 | 6.06 | 6.80E-03 |
| STM1894 | ruvB | Holliday junction DNA helicase RuvB | 1.64 | 6.53 | 3.56E-04 |
| STM1895 | ruvA | Holliday junction DNA helicase RuvA | 1.17 | 5.62 | 8.44E-03 |
| STM1898 | ruvC | Holliday junction resolvase | 2.20 | 6.11 | 1.47E-06 |
| STM1900 | ntpA | dATP pyrophosphohydrolase | 1.18 | 6.46 | 1.30E-02 |
| STM1901 | aspS | aspartyl-tRNA synthetase | 1.82 | 9.08 | 2.89E-02 |
| STM1904 | yecN | inner membrane protein | 1.23 | 4.88 | 3.62E-03 |
| STM1905 | yecO | tRNA (cmo5U34)-methyltransferase | 1.35 | 6.12 | 4.60E-04 |
| STM1906 | yecP | tRNA (mo5U34)-methyltransferase | 1.27 | 6.54 | 3.86E-03 |
| STM1915 | cheZ | chemotaxis regulator CheZ | 2.01 | 8.26 | 1.77E-02 |
| STM1916 | cheY | chemotaxis regulatory protein CheY | 1.78 | 7.30 | 7.94E-03 |
| STM1918 | cheR | chemotaxis methyltransferase CheR | 1.51 | 7.11 | 1.53E-02 |
| STM1919 | cheM | methyl accepting chemotaxis protein II | 2.05 | 8.77 | 1.28E-02 |
| STM1920 | cheW | purine-binding chemotaxis protein | 1.52 | 7.62 | 4.34E-02 |
| STM1921 | cheA | chemotaxis protein CheA | 2.02 | 9.47 | 1.40E-02 |
| STM1922 | motB | flagellar motor protein MotB | 2.04 | 7.95 | 8.19E-03 |
| STM1923 | motA | flagellar motor protein MotA | 1.88 | 8.23 | 1.70E-02 |
| STM1924.S | flhC | transcriptional activator FlhC | 1.93 | 7.81 | 1.42E-02 |
| STM1925 | flhD | transcriptional activator FlhD | 2.15 | 7.84 | 1.06E-02 |
| STM1927 | yecG | universal stress protein UspC | 1.91 | 5.47 | 1.79E-03 |
| STM1932 | ftnB | ferritin-like protein | 1.67 | 6.50 | 1.12E-02 |
| STM1936 | yecH | hypothetical protein | 1.11 | 5.30 | 2.43E-02 |
| STM1945 | pgsA | phosphatidylglycerop hosphate synthetase | 2.36 | 6.33 | 3.31E-06 |
| STM1946 | uvrC | excinuclease ABC subunit C | 1.47 | 7.83 | 2.92E-02 |
| STM1947 | uvrY | response regulator | 2.28 | 8.06 | 3.82E-03 |
| STM1949 | yecF | hypothetical protein | 1.75 | 6.23 | 5.61E-03 |
| STM1953 | yedO | D-cysteine desulfhydrase | 1.07 | 5.77 | 1.17E-02 |
| STM1956 | fliA | flagellar biosynthesis sigma factor | 2.01 | 9.16 | 4.73E-02 |
| STM1961 | fliS | flagellar protein FliS | 2.55 | 6.76 | 1.04E-04 |
| STM1962 | fliT | flagellar biosynthesis protein FliT | 1.60 | 6.46 | 1.56E-03 |
| STM1965 | yedE | inner membrane protein | 1.31 | 5.87 | 4.61E-03 |
| STM1966 | yedF | hypothetical protein | 1.51 | 4.07 | 5.55E-03 |
| STM1968 | fliE | flagellar hook-basal body protein FliE | 1.27 | 5.18 | 4.39E-03 |
| STM1970 | fliG | flagellar motor switch protein G | 1.41 | 6.91 | 2.94E-03 |
| STM1971 | fliH | flagellar assembly protein H | 1.29 | 6.22 | 9.27E-03 |
| STM1972 | fliI | flagellum-specific ATP synthase | 1.46 | 6.32 | 2.65E-04 |
| STM1974 | fliK | flagellar hook-length control protein | 1.66 | 7.18 | 2.39E-03 |
| STM1975 | fliL | flagellar basal body-associated protein FliL | 1.59 | 5.74 | 5.61E-03 |
| STM1978 | fliO | flagellar biosynthesis protein FliO | 1.42 | 5.16 | 2.67E-03 |
| STM1979 | fliP | flagellar biosynthesis protein FliP | 1.30 | 5.95 | 1.45E-03 |
| STM2059 | yeeX | hypothetical protein | 2.45 | 8.71 | 6.86E-03 |
| STM2070 | yeeZ | dehydratase | 1.23 | 6.79 | 1.40E-02 |
| STM2091 | rfbG | CDP glucose 4,6-dehydratase | 1.61 | 9.32 | 4.52E-02 |
| STM2121 | dcd | deoxycytidine triphosphate deaminase | 2.05 | 6.42 | 6.25E-06 |
| STM2122 | udk | uridine kinase | 1.12 | 6.57 | 2.19E-02 |
| STM2154 | mrp | ATPase | 2.05 | 8.97 | 8.99E-03 |
| STM2168 | pbpG | D-alanyl-D-alanine endopeptidase | 1.38 | 6.75 | 7.20E-03 |
| STM2183 | cdd | cytidine deaminase | 1.20 | 6.52 | 1.46E-02 |
| STM2184 | sanA | hypothetical protein | 1.15 | 6.36 | 2.07E-02 |
| STM2193 | folE | GTP cyclohydrolase I | 2.21 | 7.83 | 3.54E-03 |
| STM2194 | yeiG | esterase | 1.08 | 6.37 | 1.99E-02 |
| STM2202 | yeiH | inner membrane protein | 1.34 | 6.67 | 4.39E-03 |
| STM2204 | fruA | PTS system fructose-specific transporter subunitIIBC | 2.38 | 10.22 | 7.13E-03 |
| STM2205 | fruK | 1-phosphofructokinas e | 2.05 | 9.32 | 1.73E-02 |
| STM2214 | spr | outer membrane lipoprotein | 2.26 | 7.55 | 1.22E-03 |
| STM2224 | rplY | 50S ribosomal protein L25 | 2.98 | 9.00 | 2.63E-03 |
| STM2246 | narP | transcriptional regulator NarP | 1.13 | 5.83 | 7.74E-03 |
| STM2262 | eco | ecotin | 1.72 | 6.16 | 5.37E-05 |
| STM2279 | yfaE | 2Fe-2S ferredoxin | 1.49 | 4.85 | 9.12E-04 |
| STM2303 | STM2303 | hypothetical protein | 1.26 | 5.60 | 8.67E-03 |
| STM2305 | menE | O-succinylbenzoic acid--CoA ligase | 1.82 | 7.50 | 2.61E-03 |
| STM2306 | menC | O-succinylbenzoate synthase | 1.96 | 7.88 | 4.34E-03 |
| STM2307 | menB | naphthoate synthase | 1.73 | 7.92 | 2.61E-02 |
| STM2309 | menD | 2-succinyl-5-enolpyr uvyl-6-hydroxy-3-cyclohexene-1 -carboxylate synthase | 1.52 | 7.55 | 1.66E-02 |
| STM2314 | STM2314 | chemotaxis signal transduction protein | 1.81 | 7.87 | 2.58E-02 |
| STM2316.S | nuoN | NADH dehydrogenase subunit N | 1.50 | 7.72 | 2.29E-02 |
| STM2318 | nuoL | NADH dehydrogenase subunit L | 1.90 | 8.39 | 8.68E-03 |
| STM2319 | nuoK | NADH dehydrogenase subunit K | 1.40 | 5.34 | 1.42E-03 |
| STM2321 | nuoI | NADH dehydrogenase subunit I | 1.45 | 6.43 | 1.76E-03 |
| STM2322 | nuoH | NADH dehydrogenase subunit H | 1.49 | 7.32 | 7.56E-03 |
| STM2324 | nuoF | NADH dehydrogenase I subunit F | 1.66 | 7.58 | 1.23E-02 |
| STM2327 | nuoB | NADH dehydrogenase subunit B | 1.62 | 6.86 | 2.82E-03 |
| STM2328 | nuoA | NADH dehydrogenase subunit A | 1.54 | 6.17 | 4.29E-04 |
| STM2334 | yfbT | phosphatase | 2.43 | 7.30 | 1.03E-04 |
| STM2335 | yfbU | hypothetical protein | 2.26 | 7.59 | 5.92E-03 |
| STM2338 | pta | phosphate acetyltransferase | 2.23 | 11.75 | 3.47E-02 |
| STM2346 | STM2346 | NTP pyrophosphohydrolase | 1.06 | 7.04 | 2.50E-02 |
| STM2347 | yfcE | phosphodiesterase | 1.89 | 6.84 | 5.61E-04 |
| STM2354 | hisJ | histidine ABC transporter substrate-bindingprotein HisJ | 1.16 | 7.07 | 3.58E-02 |
| STM2363 | cvpA | colicin V production protein | 1.06 | 6.42 | 3.78E-02 |
| STM2364 | dedD | hypothetical protein | 1.45 | 6.76 | 2.92E-03 |
| STM2366 | accD | acetyl-CoA carboxylase subunit beta | 2.23 | 7.87 | 1.91E-03 |
| STM2369 | usg | semialdehyde dehydrogenase | 1.18 | 7.21 | 4.34E-02 |
| STM2378 | fabB | 3-oxoacyl-ACP synthase | 2.28 | 9.21 | 6.03E-03 |
| STM2380 | yfcL | hypothetical protein | 1.10 | 5.74 | 6.13E-03 |
| STM2384 | aroC | chorismate synthase | 1.21 | 7.32 | 2.85E-02 |
| STM2387 | sixA | phosphohistidine phosphatase | 1.16 | 6.59 | 1.08E-02 |
| STM2390 | yfcZ | hypothetical protein | 2.64 | 9.06 | 2.68E-03 |
| STM2392 | vacJ | lipoprotein precursor | 1.51 | 6.96 | 3.90E-03 |
| STM2402 | yfdZ | aminotransferase | 1.35 | 7.53 | 3.88E-02 |
| STM2414 | yfeD | negative regulator | 1.38 | 5.57 | 5.36E-03 |
| STM2415 | gltX | glutamyl-tRNA synthetase | 1.97 | 9.75 | 2.38E-02 |
| STM2428 | zipA | cell division protein ZipA | 2.02 | 8.55 | 1.07E-02 |
| STM2430 | cysK | cysteine synthase A | 1.01 | 6.70 | 2.35E-02 |
| STM2431 | ptsH | PTS system phosphohistidinoprotein-hexose phosphotransferase Hpr | 3.59 | 9.14 | 4.81E-04 |
| STM2432 | ptsI | phosphoenolpyruvate- protein phosphotransferase | 2.08 | 11.39 | 3.72E-02 |
| STM2433 | crr | PTS system glucose-specific transporter subunitIIA | 2.65 | 9.49 | 5.24E-03 |
| STM2449.S | STM2449.S | acetyltransferase | 1.04 | 6.17 | 1.02E-02 |
| STM2483 | dapE | succinyl-diaminopime late desuccinylase | 1.04 | 6.72 | 2.18E-02 |
| STM2488 | nlpB | lipoprotein | 2.24 | 8.86 | 5.70E-03 |
| STM2489 | dapA | dihydrodipicolinate synthase | 2.00 | 9.28 | 2.01E-02 |
| STM2490 | gcvR | glycine cleavage system transcriptionalrepressor | 1.51 | 6.10 | 8.92E-03 |
| STM2491 | bcp | thioredoxin-dependen t thiol peroxidase | 1.90 | 6.76 | 5.42E-04 |
| STM2492 | STM2492 | glycerate kinase | 1.62 | 4.85 | 2.91E-03 |
| STM2495 | yfgD | arsenate reductase | 1.91 | 5.71 | 3.07E-05 |
| STM2496 | yfgE | DNA replication initiation factor | 1.26 | 6.63 | 3.16E-03 |
| STM2502 | ppx | exopolyphosphatase | 1.18 | 7.01 | 1.94E-02 |
| STM2506 | STM2506 | inner membrane protein | 1.51 | 4.05 | 8.01E-03 |
| STM2510 | guaA | GMP synthase | 1.83 | 10.16 | 4.43E-02 |
| STM2520 | yfgL | outer membrane protein assembly complex subunitYfgL | 2.07 | 8.54 | 5.94E-03 |
| STM2523 | ispG | 4-hydroxy-3-methylbu t-2-en-1-yl diphosphatesynthase | 1.67 | 8.03 | 2.22E-02 |
| STM2524 | yfgA | cytoskeletal protein RodZ | 1.81 | 7.53 | 4.26E-03 |
| STM2536 | pepB | aminopeptidase B | 1.32 | 7.48 | 4.21E-02 |
| STM2537 | yfhJ | hypothetical protein | 1.31 | 5.27 | 1.01E-02 |
| STM2539 | hscA | chaperone protein HscA | 1.54 | 8.20 | 3.33E-02 |
| STM2541 | iscA | iron-sulfur cluster assembly protein | 1.37 | 7.22 | 3.69E-02 |
| STM2544 | yfhP | DNA-binding transcriptional regulator IscR | 2.18 | 6.68 | 2.35E-05 |
| STM2545 | STM2545 | tRNA (cytidine/uridine-2'-O-)-methy ltransferaseTrmJ | 1.56 | 6.96 | 1.22E-03 |
| STM2561 | glnB | nitrogen regulatory protein P-II 1 | 1.50 | 5.43 | 7.30E-04 |
| STM2563 | yfhG | hypothetical protein | 1.23 | 6.42 | 4.90E-03 |
| STM2577 | acpS | 4'-phosphopantethein yl transferase | 1.69 | 6.82 | 6.07E-04 |
| STM2578 | pdxJ | pyridoxine 5'-phosphate synthase | 2.17 | 7.38 | 3.49E-04 |
| STM2581 | rnc | ribonuclease III | 1.73 | 7.35 | 8.90E-03 |
| STM2582 | lepB | signal peptidase I | 1.25 | 7.18 | 3.66E-02 |
| STM2640 | rpoE | RNA polymerase sigma factor RpoE | 1.41 | 6.79 | 2.46E-03 |
| STM2646 | yfiD | autonomous glycyl radical cofactor GrcA | 2.91 | 9.74 | 1.62E-03 |
| STM2647 | ung | uracil-DNA glycosylase | 1.62 | 6.65 | 1.59E-03 |
| STM2649 | trxC | thioredoxin 2 | 2.37 | 8.67 | 1.09E-02 |
| STM2650 | yfiP | hypothetical protein | 1.51 | 6.69 | 1.69E-03 |
| STM2660 | clpB | protein disaggregation chaperone | 1.59 | 10.09 | 4.81E-02 |
| STM2661 | yfiH | hypothetical protein | 1.08 | 6.39 | 1.59E-02 |
| STM2663 | yfiO | outer membrane protein assembly complex subunitYfiO | 1.54 | 7.90 | 4.53E-02 |
| STM2665 | yfiA | translation inhibitor protein RaiA | 3.52 | 9.69 | 4.38E-03 |
| STM2673 | rplS | 50S ribosomal protein L19 | 2.11 | 9.76 | 3.67E-02 |
| STM2675 | rimM | 16S rRNA-processing protein RimM | 2.67 | 9.64 | 5.94E-03 |
| STM2676 | rpsP | 30S ribosomal protein S16 | 2.84 | 9.06 | 3.57E-03 |
| STM2681 | grpE | heat shock protein GrpE | 1.56 | 8.52 | 4.75E-02 |
| STM2685 | smpA | hypothetical protein | 1.54 | 7.35 | 2.01E-02 |
| STM2686 | yfjF | hypothetical protein | 1.40 | 5.74 | 1.18E-03 |
| STM2687 | yfjG | hypothetical protein | 1.57 | 6.90 | 2.63E-03 |
| STM2800 | STM2800 | inner membrane protein | 1.88 | 7.60 | 1.18E-02 |
| STM2817 | luxS | S-ribosylhomocystein ase | 2.36 | 7.16 | 3.06E-04 |
| STM2820 | yqaB | fructose-1-phosphata se | 1.62 | 7.10 | 3.63E-03 |
| STM2826 | csrA | carbon storage regulator | 2.72 | 8.02 | 3.33E-03 |
| STM2843 | hydN | electron transport protein HydN | 2.11 | 8.12 | 8.90E-03 |
| STM2844 | STM2844 | hypothetical protein | 1.35 | 7.13 | 5.50E-03 |
| STM2846 | hycH | hydrogenase 3 large subunit processing protein | 1.88 | 6.43 | 2.38E-04 |
| STM2847 | hycG | hydrogenase | 2.38 | 8.03 | 3.86E-03 |
| STM2849 | hycE | hydrogenase 3 large subunit | 2.25 | 9.74 | 7.42E-03 |
| STM2850 | hycD | hydrogenase 3 membrane subunit | 2.20 | 8.64 | 4.42E-03 |
| STM2851 | hycC | formate hydrogenlyase subunit 3 | 2.43 | 9.42 | 2.92E-03 |
| STM2852 | hycB | hydrogenase-3 iron-sulfur subunit | 2.38 | 8.30 | 2.19E-03 |
| STM2853 | hycA | formate hydrogenlyase regulatory protein HycA | 2.11 | 7.98 | 1.14E-02 |
| STM2855 | hypB | hydrogenase nickel incorporation protein HypB | 1.71 | 8.04 | 3.01E-02 |
| STM2857 | hypD | hydrogenase formation protein | 1.86 | 8.07 | 1.38E-02 |
| STM2858 | hypE | hydrogenase formation protein | 1.82 | 7.71 | 9.43E-03 |
| STM2924 | rpoS | RNA polymerase sigma factor RpoS | 1.54 | 8.49 | 4.43E-02 |
| STM2927 | surE | stationary phase survival protein SurE | 1.72 | 6.93 | 3.74E-04 |
| STM2929 | ispF | 2-C-methyl-D-erythri tol 2,4-cyclodiphosphatesynthase | 1.01 | 5.94 | 1.28E-02 |
| STM2930 | ispD | 2-C-methyl-D-erythri tol 4-phosphatecytidylyltransferas e | 1.19 | 6.76 | 2.06E-02 |
| STM2952 | eno | phosphopyruvate hydratase | 2.16 | 12.42 | 4.76E-02 |
| STM2955.S | STM2955.S | transcriptional regulator | 1.24 | 5.57 | 1.76E-02 |
| STM2965 | yqcC | hypothetical protein | 1.03 | 4.81 | 2.67E-02 |
| STM2968 | queF | 7-cyano-7-deazaguani ne reductase | 1.05 | 6.40 | 1.34E-02 |
| STM2969 | ygdH | nucleotide binding | 1.99 | 8.42 | 1.21E-02 |
| STM2970 | sdaC | serine transport protein | 2.01 | 9.21 | 1.66E-02 |
| STM2971 | sdaB | L-serine dehydratase/L-threonine deaminase 2 | 1.92 | 8.56 | 1.84E-02 |
| STM3001 | thyA | thymidylate synthase | 1.08 | 7.00 | 3.73E-02 |
| STM3004 | ygdP | dinucleoside polyphosphate hydrolase | 1.05 | 6.60 | 2.08E-02 |
| STM3034 | STM3034 | antitoxin VapB | 1.52 | 5.27 | 1.80E-03 |
| STM3040 | lysS | lysyl-tRNA synthetase | 2.08 | 10.98 | 2.89E-02 |
| STM3045 | fldB | flavodoxin FldB | 1.36 | 6.63 | 3.20E-03 |
| STM3046 | ygfX | inner membrane protein | 1.26 | 6.27 | 3.58E-03 |
| STM3047 | ygfY | hypothetical protein | 1.30 | 5.99 | 5.09E-03 |
| STM3048 | ygfZ | global regulator | 1.77 | 7.76 | 1.43E-02 |
| STM3053 | gcvP | glycine dehydrogenase | 1.81 | 8.95 | 2.55E-02 |
| STM3054 | gcvH | glycine cleavage system protein H | 2.33 | 6.84 | 2.80E-04 |
| STM3055 | gcvT | glycine cleavage system aminomethyltransferaseT | 1.46 | 7.88 | 5.00E-02 |
| STM3057 | ubiH | 2-octaprenyl-6-metho xyphenyl hydroxylase | 2.16 | 7.84 | 2.81E-03 |
| STM3058 | pepP | proline aminopeptidase P II | 1.46 | 8.01 | 3.83E-02 |
| STM3059.S | ygfB | hypothetical protein | 1.33 | 6.93 | 2.39E-02 |
| STM3069 | pgk | phosphoglycerate kinase | 2.41 | 11.27 | 1.57E-02 |
| STM3070 | epd | erythrose 4-phosphate dehydrogenase | 1.79 | 9.00 | 1.61E-02 |
| STM3072 | STM3072 | inner membrane protein | 1.19 | 5.39 | 2.01E-02 |
| STM3076 | tktA | transketolase | 1.90 | 10.80 | 3.81E-02 |
| STM3078 | speB | agmatinase | 1.59 | 7.84 | 2.94E-02 |
| STM3095 | gshB | glutathione synthetase | 1.32 | 7.18 | 1.21E-02 |
| STM3101 | yggT | integral membrane protein | 1.32 | 6.86 | 1.46E-02 |
| STM3103 | yggV | deoxyribonucleotide triphosphatepyrophosphatase | 1.27 | 6.64 | 6.84E-03 |
| STM3143 | hybG | hydrogenase 2 accessory protein HypG | 1.31 | 6.65 | 9.92E-03 |
| STM3145 | hybE | hydrogenase 2-specific chaperone | 2.17 | 6.66 | 7.52E-06 |
| STM3147 | hybC | hydrogenase 2 large subunit | 1.75 | 9.27 | 2.55E-02 |
| STM3148 | hybB | hydrogenase 2 b cytochrome subunit | 1.29 | 7.64 | 4.52E-02 |
| STM3150 | hypO | hydrogenase 2 small subunit | 1.73 | 8.48 | 2.27E-02 |
| STM3162 | yghB | hypothetical protein | 1.03 | 6.33 | 9.86E-03 |
| STM3172 | sufI | repressor protein for FtsI | 1.09 | 7.37 | 4.39E-02 |
| STM3176 | ygiW | outer membrane protein | 1.61 | 7.50 | 3.25E-02 |
| STM3180 | ygiN | hypothetical protein | 1.69 | 6.34 | 2.07E-04 |
| STM3182 | yqiA | esterase YqiA | 1.24 | 6.30 | 3.26E-03 |
| STM3183 | icc | cyclic 3',5'-adenosine monophosphatephosphodiesterase | 1.54 | 7.20 | 3.76E-03 |
| STM3187 | ygiB | hypothetical protein | 2.50 | 7.28 | 1.30E-04 |
| STM3188 | ygiC | glutathionylspermidi ne synthase | 1.37 | 7.74 | 4.07E-02 |
| STM3208 | gcp | DNA-binding/iron metalloprotein/AP endonuclease | 1.43 | 6.99 | 3.78E-03 |
| STM3209 | rpsU | 30S ribosomal protein S21 | 3.03 | 8.68 | 3.01E-03 |
| STM3211.S | rpoD | RNA polymerase sigma factor RpoD | 1.57 | 9.54 | 4.44E-02 |
| STM3229 | yqjD | inner membrane protein | 1.98 | 6.83 | 4.40E-04 |
| STM3230 | yqjE | inner membrane protein | 1.33 | 6.13 | 4.17E-03 |
| STM3231 | yqjK | inner membrane protein | 2.04 | 5.94 | 1.12E-05 |
| STM3234 | yhaH | inner membrane protein | 1.03 | 5.46 | 3.69E-02 |
| STM3237 | yhaL | hypothetical protein | 1.33 | 4.25 | 1.93E-02 |
| STM3238 | yhaN | inner membrane protein | 2.18 | 9.17 | 4.23E-03 |
| STM3273 | yhbT | lipid carrier protein | 1.65 | 7.43 | 6.50E-03 |
| STM3282 | pnp | polynucleotide phosphorylase/polyadenylase | 1.67 | 10.15 | 4.56E-02 |
| STM3283 | rpsO | 30S ribosomal protein S15 | 2.68 | 8.53 | 5.36E-03 |
| STM3284 | truB | tRNA pseudouridine synthase B | 1.56 | 7.37 | 7.23E-03 |
| STM3285 | rbfA | ribosome-binding factor A | 1.72 | 6.40 | 6.67E-04 |
| STM3293 | secG | preprotein translocase subunit SecG | 1.78 | 8.46 | 3.81E-02 |
| STM3296 | hflB | ATP-dependent metalloprotease | 2.06 | 10.51 | 1.58E-02 |
| STM3297 | rrmJ | 23S rRNA methyltransferase J | 2.37 | 8.53 | 4.14E-03 |
| STM3298.S | yhbY | RNA-binding protein YhbY | 1.72 | 7.00 | 5.01E-03 |
| STM3299 | greA | transcription elongation factor GreA | 1.60 | 7.33 | 1.27E-02 |
| STM3305 | ispB | octaprenyl diphosphate synthase | 1.62 | 7.80 | 1.78E-02 |
| STM3308 | yrbA | transcriptional regulator | 1.25 | 5.48 | 8.83E-03 |
| STM3309 | yrbB | hypothetical protein | 1.23 | 6.20 | 2.11E-02 |
| STM3318 | yhbN | lipopolysaccharide transport periplasmic proteinLptA | 1.27 | 7.28 | 4.10E-02 |
| STM3319 | yhbG | ABC transporter ATP-binding protein | 1.24 | 7.03 | 2.64E-02 |
| STM3321 | yhbH | sigma(54) modulation protein | 1.65 | 7.21 | 1.69E-02 |
| STM3322 | ptsN | PTS system transporter subunit IIA-likenitrogen-regulatory protein PtsN | 1.16 | 6.85 | 2.12E-02 |
| STM3323 | yhbJ | hypothetical protein | 1.24 | 7.33 | 2.09E-02 |
| STM3327 | yhbL | isoprenoid biosynthesis protein | 1.40 | 7.19 | 2.62E-02 |
| STM3341 | sspB | ClpXP protease specificity-enhancing factor | 1.95 | 7.55 | 3.25E-03 |
| STM3342 | sspA | stringent starvation protein A | 2.06 | 8.59 | 1.01E-02 |
| STM3344 | rpsI | 30S ribosomal protein S9 | 2.43 | 9.99 | 1.17E-02 |
| STM3347 | yhcB | cytochrome d ubiquinol oxidase subunit III | 2.31 | 7.85 | 3.20E-03 |
| STM3359 | mdh | malate dehydrogenase | 1.47 | 7.63 | 3.39E-02 |
| STM3374 | mreB | rod shape-determining protein MreB | 1.67 | 8.33 | 2.83E-02 |
| STM3374.1n | STM3374.1n | hypothetical protein | 1.87 | 5.22 | 2.02E-05 |
| STM3376 | yhdH | oxidoreductase | 1.25 | 7.34 | 2.61E-02 |
| STM3379 | accB | acetyl-CoA carboxylase biotin carboxyl carrierprotein subunit | 1.81 | 8.04 | 2.39E-02 |
| STM3402 | yrdC | ribosome maturation factor | 1.67 | 5.93 | 3.55E-04 |
| STM3403 | yrdD | DNA topoisomerase | 1.57 | 6.81 | 3.62E-03 |
| STM3404 | smg | hypothetical protein | 2.29 | 7.71 | 5.67E-03 |
| STM3406 | def | peptide deformylase | 1.51 | 7.81 | 4.17E-02 |
| STM3407 | fmt | methionyl-tRNA formyltransferase | 1.52 | 7.73 | 3.17E-02 |
| STM3414 | rplQ | 50S ribosomal protein L17 | 2.39 | 10.35 | 2.14E-02 |
| STM3415 | rpoA | DNA-directed RNA polymerase subunit alpha | 2.34 | 11.85 | 2.78E-02 |
| STM3417 | rpsK | 30S ribosomal protein S11 | 2.64 | 10.15 | 1.23E-02 |
| STM3420 | secY | preprotein translocase subunit SecY | 2.51 | 11.69 | 1.10E-02 |
| STM3421 | rplO | 50S ribosomal protein L15 | 2.20 | 10.44 | 2.60E-02 |
| STM3422 | rpmD | 50S ribosomal protein L30 | 2.78 | 8.60 | 5.82E-03 |
| STM3423 | rpsE | 30S ribosomal protein S5 | 2.40 | 10.16 | 1.23E-02 |
| STM3424 | rplR | 50S ribosomal protein L18 | 2.64 | 9.76 | 1.32E-02 |
| STM3432 | rpmC | 50S ribosomal protein L29 | 2.93 | 9.97 | 4.65E-03 |
| STM3433 | rplP | 50S ribosomal protein L16 | 2.34 | 10.28 | 2.27E-02 |
| STM3434 | rpsC | 30S ribosomal protein S3 | 2.59 | 11.25 | 1.27E-02 |
| STM3435 | rplV | 50S ribosomal protein L22 | 2.47 | 10.47 | 1.63E-02 |
| STM3437 | rplB | 50S ribosomal protein L2 | 2.44 | 11.28 | 2.21E-02 |
| STM3439 | rplD | 50S ribosomal protein L4 | 2.46 | 10.92 | 1.27E-02 |
| STM3440 | rplC | 50S ribosomal protein L3 | 2.05 | 11.17 | 4.66E-02 |
| STM3447 | rpsG | 30S ribosomal protein S7 | 2.22 | 10.09 | 3.57E-02 |
| STM3448 | rpsL | 30S ribosomal protein S12 | 2.51 | 10.39 | 2.23E-02 |
| STM3451 | yheN | sulfur transfer complex subunit TusD | 1.02 | 5.68 | 1.21E-02 |
| STM3453 | fkpA | FKBP-type peptidyl-prolyl cis-trans isomerase | 1.74 | 9.08 | 3.12E-02 |
| STM3454 | slyX | hypothetical protein | 1.17 | 5.19 | 1.22E-02 |
| STM3455 | slyD | FKBP-type peptidyl-prolyl cis-trans isomerase | 1.83 | 9.08 | 2.49E-02 |
| STM3465 | yhfA | hypothetical protein | 1.34 | 6.00 | 1.06E-03 |
| STM3466 | crp | cAMP-regulatory protein | 2.03 | 7.82 | 9.74E-03 |
| STM3481 | trpS | tryptophanyl-tRNA synthetase | 1.42 | 8.32 | 4.98E-02 |
| STM3484 | dam | DNA adenine methylase | 1.17 | 6.95 | 2.50E-02 |
| STM3486 | aroB | 3-dehydroquinate synthase | 1.49 | 8.37 | 4.92E-02 |
| STM3502 | ompR | osmolarity response regulator | 1.03 | 6.64 | 1.53E-02 |
| STM3568 | rpoH | RNA polymerase factor sigma-32 | 1.75 | 9.09 | 2.59E-02 |
| STM3569 | ftsX | cell division protein FtsX | 1.16 | 7.28 | 2.40E-02 |
| STM3570 | ftsE | cell division protein FtsE | 1.55 | 7.26 | 3.21E-03 |
| STM3575 | yhhN | inner membrane protein | 1.27 | 6.44 | 8.80E-03 |
| STM3578 | yhhP | sulfur transfer protein SirA | 1.64 | 6.03 | 3.58E-04 |
| STM3596 | yhiR | hypothetical protein | 1.10 | 6.85 | 1.48E-02 |
| STM3647 | yiaF | outer membrane lipoprotein | 1.54 | 8.40 | 2.95E-02 |
| STM3649 | cspA | major cold shock protein | 2.02 | 8.56 | 9.00E-03 |
| STM3655 | glyS | glycyl-tRNA synthetase subunit beta | 1.57 | 9.20 | 3.92E-02 |
| STM3656 | glyQ | glycyl-tRNA synthetase subunit alpha | 1.89 | 7.73 | 8.20E-03 |
| STM3683 | selA | selenocysteine synthase | 1.09 | 6.86 | 1.21E-02 |
| STM3699 | cysE | serine acetyltransferase | 1.09 | 7.34 | 4.39E-02 |
| STM3700 | gpsA | NAD(P)H-dependent glycerol-3-phosphatedehydrogen ase | 1.95 | 8.12 | 7.85E-03 |
| STM3701 | secB | preprotein translocase subunit SecB | 2.11 | 8.70 | 9.72E-03 |
| STM3703 | yibN | rhodanese-like sulfurtransferase | 1.16 | 6.91 | 3.01E-02 |
| STM3704 | pmgI | phosphoglyceromutase | 1.88 | 9.94 | 2.64E-02 |
| STM3710 | rfaD | ADP-L-glycero-D-mann o-heptose-6-epimerase | 1.74 | 8.10 | 2.12E-02 |
| STM3711 | rfaF | ADP-heptose--LPS heptosyltransferase | 1.70 | 7.87 | 1.54E-02 |
| STM3725 | coaD | phosphopantetheine adenylyltransferase | 1.20 | 6.45 | 4.36E-03 |
| STM3728 | rpmB | 50S ribosomal protein L28 | 2.34 | 9.45 | 1.79E-02 |
| STM3731 | dut | deoxyuridine 5'-triphosphatenucleotidohydro lase | 1.27 | 6.65 | 1.33E-02 |
| STM3740 | gmk | guanylate kinase | 1.59 | 7.22 | 3.41E-03 |
| STM3741 | rpoZ | DNA-directed RNA polymerase subunit omega | 2.49 | 6.54 | 1.34E-06 |
| STM3808.S | ibpB | heat shock chaperone IbpB | 1.01 | 5.85 | 1.12E-02 |
| STM3839 | rpmH | 50S ribosomal protein L34 | 1.82 | 6.72 | 1.94E-03 |
| STM3857 | pstS | phosphate ABC transporter substrate-bindingprotein | 2.13 | 8.76 | 1.11E-02 |
| STM3861 | glmS | glucosamine--fructos e-6-phosphateaminotransferase | 2.17 | 10.23 | 7.91E-03 |
| STM3862 | glmU | bifunctional N-acetylglucosamine-1-phosphat euridyltransferase/glucosamine -1-phosphateacetyltransferase | 1.69 | 9.21 | 2.50E-02 |
| STM3864 | atpC | F0F1 ATP synthase subunit epsilon | 2.42 | 9.64 | 1.14E-02 |
| STM3865 | atpD | F0F1 ATP synthase subunit beta | 2.38 | 11.28 | 1.21E-02 |
| STM3867 | atpA | F0F1 ATP synthase subunit alpha | 2.31 | 11.39 | 1.38E-02 |
| STM3868 | atpH | F0F1 ATP synthase subunit delta | 2.21 | 9.08 | 1.13E-02 |
| STM3870 | atpE | F0F1 ATP synthase subunit C | 2.81 | 9.32 | 1.79E-03 |
| STM3871 | atpB | F0F1 ATP synthase subunit A | 1.70 | 8.89 | 2.40E-02 |
| STM3872 | atpI | F0F1 ATP synthase subunit I | 1.22 | 7.00 | 1.46E-02 |
| STM3875 | mioC | flavodoxin | 1.05 | 6.37 | 3.15E-02 |
| STM3877 | asnA | asparagine synthetase AsnA | 1.67 | 8.59 | 4.34E-02 |
| STM3897 | yifA | transcriptional regulator HdfR | 1.52 | 7.07 | 2.67E-02 |
| STM3910 | ppiC | peptidyl-prolyl cis-trans isomerase C | 1.36 | 5.55 | 2.10E-03 |
| STM3914 | rhlB | ATP-dependent RNA helicase RhlB | 1.47 | 8.47 | 4.17E-02 |
| STM3915 | trxA | thioredoxin | 1.81 | 8.32 | 2.63E-02 |
| STM3937 | hemD | uroporphyrinogen-III synthase | 1.22 | 6.41 | 3.57E-03 |
| STM3938 | hemC | porphobilinogen deaminase | 1.75 | 7.64 | 1.71E-02 |
| STM3943 | cyaY | frataxin-like protein | 1.82 | 6.19 | 7.89E-05 |
| STM3947 | dapF | diaminopimelate epimerase | 1.51 | 7.50 | 2.12E-02 |
| STM3948 | yigA | hypothetical protein | 1.20 | 6.84 | 1.79E-02 |
| STM3950 | yigB | flavin mononucleotide phosphatase | 1.05 | 6.09 | 1.71E-02 |
| STM3973 | tatA | twin arginine translocase protein A | 1.36 | 7.30 | 1.81E-02 |
| STM3995 | yihD | hypothetical protein | 1.31 | 6.20 | 2.09E-02 |
| STM4007 | glnA | glutamine synthetase | 1.91 | 10.65 | 3.57E-02 |
| STM4034 | fdhE | formate dehydrogenase accessory protein FdhE | 1.40 | 6.98 | 3.57E-03 |
| STM4058 | cpxA | two-component sensor protein | 1.20 | 7.53 | 4.67E-02 |
| STM4060 | cpxP | repressor CpxP | 1.18 | 7.10 | 3.70E-02 |
| STM4062 | pfkA | 6-phosphofructokinas e | 1.61 | 9.60 | 4.18E-02 |
| STM4084 | fpr | ferredoxin-NADP reductase | 1.00 | 6.69 | 3.03E-02 |
| STM4088 | yiiU | hypothetical protein | 2.07 | 8.39 | 1.48E-02 |
| STM4092 | hslV | ATP-dependent protease peptidase subunit | 1.14 | 7.28 | 3.37E-02 |
| STM4096 | rpmE | 50S ribosomal protein L31 | 2.14 | 8.55 | 1.66E-02 |
| STM4099 | metJ | transcriptional repressor protein MetJ | 1.25 | 6.70 | 1.43E-02 |
| STM4106 | katG | hydroperoxidase | 2.09 | 10.35 | 1.29E-02 |
| STM4119 | ppc | phosphoenolpyruvate carboxylase | 1.88 | 9.47 | 3.59E-02 |
| STM4120 | argE | acetylornithine deacetylase | 1.40 | 6.81 | 3.84E-02 |
| STM4125 | oxyR | DNA-binding transcriptional regulator OxyR | 1.73 | 8.05 | 1.65E-02 |
| STM4147 | secE | preprotein translocase subunit SecE | 1.87 | 8.29 | 1.56E-02 |
| STM4148 | nusG | transcription antitermination protein NusG | 2.07 | 8.30 | 1.11E-02 |
| STM4149 | rplK | 50S ribosomal protein L11 | 2.31 | 10.03 | 2.56E-02 |
| STM4151 | rplJ | 50S ribosomal protein L10 | 2.31 | 10.71 | 3.65E-02 |
| STM4152 | rplL | 50S ribosomal protein L7/L12 | 2.52 | 10.43 | 3.03E-02 |
| STM4153 | rpoB | DNA-directed RNA polymerase subunit beta | 1.85 | 11.45 | 3.66E-02 |
| STM4154 | rpoC | DNA-directed RNA polymerase subunit beta' | 2.13 | 11.76 | 1.50E-02 |
| STM4171 | yjaH | inner membrane protein | 1.12 | 6.28 | 5.48E-03 |
| STM4265 | soxS | DNA-binding transcriptional regulator SoxS | 1.58 | 6.47 | 3.22E-03 |
| STM4294 | yjdE | arginine:agmatin antiporter | 1.94 | 9.63 | 1.82E-02 |
| STM4325 | dcuA | anaerobic C4-dicarboxylate transporter | 2.21 | 10.12 | 8.76E-03 |
| STM4330 | groEL | chaperonin GroEL | 2.23 | 12.33 | 2.68E-02 |
| STM4331 | yjeI | outer membrane lipoprotein | 2.78 | 7.92 | 1.51E-03 |
| STM4340 | frdD | fumarate reductase subunit D | 2.04 | 9.17 | 2.20E-02 |
| STM4341 | frdC | fumarate reductase subunit C | 2.04 | 7.84 | 2.91E-02 |
| STM4360 | miaA | tRNA delta(2)-isopentenylpyrophosph atetransferase | 1.56 | 7.69 | 2.00E-02 |
| STM4362 | hflX | GTPase HflX | 1.52 | 8.55 | 4.40E-02 |
| STM4364 | hflC | FtsH protease regulator HflC | 1.42 | 8.22 | 4.61E-02 |
| STM4366 | purA | adenylosuccinate synthetase | 2.24 | 9.77 | 5.05E-03 |
| STM4367 | yjeB | transcriptional repressor NsrR | 1.62 | 6.42 | 1.88E-03 |
| STM4369 | yjfH | 23S rRNA (guanosine-2'-O-)-methyltransf erase | 1.47 | 6.85 | 2.22E-03 |
| STM4379 | yjfO | biofilm stress and motility protein A | 1.02 | 4.76 | 2.75E-02 |
| STM4380 | yjfP | esterase | 1.07 | 6.17 | 3.80E-02 |
| STM4394 | rplI | 50S ribosomal protein L9 | 2.45 | 9.69 | 8.61E-03 |
| STM4397 | fklB | peptidyl-prolyl cis-trans isomerase | 1.47 | 7.09 | 5.58E-03 |
| STM4411 | ytfP | hypothetical protein | 1.06 | 6.59 | 2.03E-02 |
| STM4478 | STM4478 | hypothetical protein | 1.19 | 2.92 | 4.75E-02 |
| STM4512 | iadA | isoaspartyl dipeptidase | 1.67 | 7.43 | 6.18E-03 |
| STM4532 | yjiY | carbon starvation protein | 1.66 | 9.43 | 3.16E-02 |
| STM4544 | dnaT | primosomal protein DnaI | 1.01 | 6.16 | 2.60E-02 |
| STM4549 | STM4549 | hypothetical protein | 1.75 | 6.39 | 1.26E-03 |
| STM4563 | yjjU | phosphoesterase | 1.29 | 6.81 | 3.58E-03 |
| STM4564 | yjjV | deoxyribonuclease YjjV | 1.18 | 5.70 | 3.11E-03 |
| STM4570 | deoD | purine nucleoside phosphorylase | 1.44 | 7.78 | 4.03E-02 |
| STM4581 | yjjK | ABC transporter ATP-binding protein | 1.57 | 8.84 | 3.78E-02 |
| STM4585 | gpmB | phosphoglycerate mutase | 1.41 | 6.90 | 4.51E-03 |
| STM4586 | rob | transcriptional regulator | 1.44 | 7.68 | 3.73E-02 |
| STM4598 | arcA | two-component response regulator | 1.79 | 9.23 | 2.90E-02 |

Table S2: *Salmonella* Typhimurium N-15 genes higher expressed in co-culture with RBL67.

| **ORF** | **Gene** | **Function** | **logFC** | **logCPM** | **FDR** |
| --- | --- | --- | --- | --- | --- |
| STM0010 | htgA | hypothetical protein | -1.26 | 4.76 | 8.28E-03 |
| STM0011 | yaaI | hypothetical protein | -2.01 | 4.23 | 3.67E-05 |
| STM0014 | STM0014 | transcriptional regulator | -1.40 | 5.43 | 2.92E-02 |
| STM0015 | STM0015 | bacteriophage protein | -2.25 | 3.39 | 2.43E-04 |
| STM0016 | STM0016 | hypothetical protein | -2.49 | 4.95 | 2.35E-04 |
| STM0017 | STM0017 | hypothetical protein | -2.22 | 4.20 | 3.28E-04 |
| STM0018 | STM0018 | exochitinase | -1.51 | 6.41 | 2.24E-04 |
| STM0019 | STM0019 | hydroxymethyltransferase | -1.90 | 6.90 | 6.33E-05 |
| STM0020 | STM0020 | hypothetical protein | -2.63 | 4.65 | 2.68E-06 |
| STM0022 | bcfB | fimbrial chaperone | -1.91 | 4.67 | 2.36E-04 |
| STM0023 | bcfC | fimbrial usher | -1.83 | 6.84 | 2.39E-04 |
| STM0024 | bcfD | fimbrial subunit | -1.68 | 5.61 | 2.28E-04 |
| STM0025 | bcfE | fimbrial subunit | -1.72 | 4.59 | 3.12E-04 |
| STM0027 | bcfG | fimbrial chaperone | -1.56 | 4.72 | 1.62E-03 |
| STM0028 | bcfH | thiol-disulfide isomerase | -1.55 | 5.51 | 1.73E-03 |
| STM0028.1n | STM0028.1n | hypothetical protein | -1.40 | 4.28 | 5.74E-03 |
| STM0029 | STM0029 | transcriptional regulator | -1.89 | 3.38 | 1.09E-03 |
| STM0030 | STM0030 | transcriptional regulator | -2.00 | 5.25 | 2.11E-03 |
| STM0031 | STM0031 | transcriptional regulator | -2.31 | 4.71 | 4.44E-03 |
| STM0032 | STM0032 | arylsulfatase | -1.91 | 6.53 | 1.09E-04 |
| STM0033 | STM0033 | 5'-nucleotidase | -1.32 | 6.37 | 2.20E-03 |
| STM0034 | STM0034 | outer membrane/exported protein | -2.21 | 5.07 | 1.79E-04 |
| STM0035 | STM0035 | arylsulfatase | -1.25 | 6.44 | 2.35E-03 |
| STM0036 | STM0036 | arylsulfatase regulator | -1.45 | 5.73 | 5.92E-04 |
| STM0037 | STM0037 | hypothetical protein | -2.15 | 5.64 | 3.40E-06 |
| STM0038 | STM0038 | arylsulfatase | -1.58 | 6.55 | 3.24E-04 |
| STM0041 | STM0041 | glycosyl hydrolase | -1.85 | 6.36 | 1.64E-05 |
| STM0042 | STM0042 | sodium galactoside symporter | -2.19 | 6.06 | 5.93E-05 |
| STM0044 | yaaY | hypothetical protein | -1.49 | 2.60 | 1.21E-02 |
| STM0053 | STM0053 | signal transduction histidine kinase | -1.54 | 6.62 | 3.80E-04 |
| STM0055 | STM0055 | oxaloacetate decarboxylase | -1.93 | 6.26 | 1.14E-02 |
| STM0056 | STM0056 | oxaloacetate decarboxylase subunit gamma | -2.00 | 3.59 | 1.21E-03 |
| STM0057 | STM0057 | citrate-sodium symporter | -2.33 | 5.49 | 1.52E-05 |
| STM0058 | citC2 | citrate lyase synthetase | -1.52 | 5.61 | 7.65E-04 |
| STM0059 | citD2 | citrate lyase subunit gamma | -2.23 | 3.44 | 7.45E-05 |
| STM0060 | citE2 | citrate lyase subunit beta | -2.20 | 5.17 | 2.31E-05 |
| STM0061 | citF2 | citrate lyase subunit alpha/citrate-ACPtransferase | -1.90 | 6.19 | 1.98E-05 |
| STM0062 | citX2 | hypothetical protein | -2.18 | 4.43 | 5.05E-05 |
| STM0063 | citG2 | triphosphoribosyl-de phospho-CoA synthase | -1.84 | 5.64 | 7.32E-05 |
| STM0070 | caiD | carnitinyl-CoA dehydratase | -1.68 | 4.87 | 2.62E-04 |
| STM0071 | caiC | crotonobetaine/carni tine-CoA ligase | -1.35 | 6.44 | 1.43E-03 |
| STM0074 | caiT | L-carnitine/gamma-bu tyrobetaine antiporter | -1.34 | 6.32 | 1.68E-03 |
| STM0075 | fixA | electron transfer flavoprotein FixA | -2.50 | 5.20 | 1.80E-06 |
| STM0076 | fixB | electron transfer flavoprotein FixB | -1.67 | 5.09 | 6.49E-04 |
| STM0077 | fixC | oxidoreductase FixC | -1.79 | 5.49 | 3.94E-04 |
| STM0078 | fixX | ferredoxin | -1.78 | 3.51 | 1.05E-03 |
| STM0079 | yaaU | transporter | -1.47 | 5.91 | 2.73E-04 |
| STM0081 | STM0081 | hypothetical protein | -1.67 | 4.91 | 2.71E-04 |
| STM0084 | STM0084 | sulfatase | -2.12 | 6.36 | 2.95E-06 |
| STM0086 | kefC | glutathione-regulate d potassium-efflux systemprotein KefC | -1.41 | 6.94 | 4.29E-03 |
| STM0098 | STM0098 | hypothetical protein | -1.22 | 4.31 | 8.94E-03 |
| STM0100 | STM0100 | hypothetical protein | -1.96 | 3.60 | 1.87E-03 |
| STM0101 | araD | L-ribulose-5-phospha te 4-epimerase | -1.79 | 4.82 | 3.12E-04 |
| STM0102 | araA | L-arabinose isomerase | -1.89 | 5.81 | 2.45E-05 |
| STM0103 | araB | ribulokinase | -1.24 | 5.84 | 5.06E-03 |
| STM0110 | leuD | isopropylmalate isomerase small subunit | -1.41 | 4.64 | 2.40E-03 |
| STM0115 | leuO | leucine transcriptional activator | -2.87 | 5.25 | 3.54E-08 |
| STM0142 | hofC | type IV pilin biogenesis protein | -1.13 | 5.90 | 3.52E-03 |
| STM0143 | hofB | hypothetical protein | -2.22 | 5.45 | 2.47E-06 |
| STM0144 | ppdD | major pilin subunit | -2.18 | 4.07 | 9.43E-05 |
| STM0149 | STM0149 | Na+/galactoside symporter | -2.20 | 6.04 | 5.15E-06 |
| STM0155 | STM0155 | outer membrane protein | -1.52 | 3.76 | 4.21E-03 |
| STM0157 | yacH | outer membrane protein | -1.45 | 6.27 | 7.54E-04 |
| STM0161 | kdgT | 2-keto-3-deoxyglucon ate permease | -1.90 | 5.43 | 3.32E-04 |
| STM0162 | STM0162 | inner membrane protein | -1.68 | 5.98 | 1.49E-04 |
| STM0174 | stiH | fimbrial protein precurosr | -1.67 | 6.43 | 1.58E-04 |
| STM0175 | stiC | fimbrial usher | -2.24 | 6.60 | 3.18E-06 |
| STM0176 | stiB | fimbrial chaperone | -2.49 | 4.68 | 2.56E-06 |
| STM0177 | stiA | fimbrial subunit | -1.82 | 4.39 | 7.25E-04 |
| STM0191 | fhuA | ferrichrome outer membrane transporter | -1.24 | 6.98 | 8.94E-03 |
| STM0192 | fhuC | iron-hydroxamate transporter ATP-bindingsubunit | -1.52 | 4.49 | 9.70E-04 |
| STM0193 | fhuD | iron-hydroxamate transporter substrate-bindingsubunit | -1.75 | 4.51 | 2.99E-04 |
| STM0194 | fhuB | iron-hydroxamate transporter permease subunit | -1.21 | 5.95 | 1.20E-02 |
| STM0195 | stfA | fimbrial subunit | -2.02 | 4.35 | 4.28E-05 |
| STM0196 | stfC | fimbrial outer membrane usher | -2.12 | 6.70 | 1.23E-05 |
| STM0197 | stfD | periplasmic fimbrial chaperone | -2.10 | 4.73 | 4.98E-05 |
| STM0198 | stfE | minor fimbrial subunit | -2.50 | 5.13 | 1.97E-04 |
| STM0199 | stfF | minor fimbrial subunit | -1.70 | 4.24 | 2.71E-03 |
| STM0200 | stfG | minor fimbrial subunit | -1.29 | 3.94 | 2.33E-02 |
| STM0257 | STM0257 | drug efflux protein | -1.35 | 5.38 | 1.15E-03 |
| STM0267 | STM0267 | hypothetical protein | -1.27 | 4.98 | 7.19E-03 |
| STM0268 | STM0268 | hypothetical protein | -1.40 | 5.93 | 2.92E-03 |
| STM0269 | STM0269 | hypothetical protein | -1.63 | 3.73 | 1.28E-03 |
| STM0271 | STM0271 | hypothetical protein | -1.99 | 5.63 | 4.46E-05 |
| STM0272 | STM0272 | chaperone ATPase | -1.49 | 6.57 | 1.83E-03 |
| STM0273 | STM0273 | hypothetical protein | -1.90 | 4.36 | 2.02E-04 |
| STM0274 | STM0274 | hypothetical protein | -1.74 | 6.04 | 2.15E-05 |
| STM0274A | STM0274A | invasol SirA | -2.85 | 2.97 | 1.54E-05 |
| STM0275.s | STM0275.s | hypothetical protein | -2.21 | 4.32 | 2.42E-04 |
| STM0276 | STM0276 | hypothetical protein | -1.47 | 3.70 | 1.61E-02 |
| STM0277 | STM0277 | hypothetical protein | -2.47 | 4.76 | 1.43E-05 |
| STM0278 | STM0278 | hypothetical protein | -2.32 | 3.79 | 3.81E-05 |
| STM0279 | STM0279 | hypothetical protein | -2.64 | 3.99 | 5.01E-06 |
| STM0280 | STM0280 | outer membrane lipoprotein | -2.90 | 4.39 | 2.68E-06 |
| STM0281 | STM0281 | hypothetical protein | -2.05 | 5.48 | 2.34E-04 |
| STM0282 | STM0282 | hypothetical protein | -2.23 | 4.97 | 3.86E-06 |
| STM0283 | STM0283 | inner membrane protein | -1.89 | 5.62 | 4.93E-04 |
| STM0284 | STM0284 | Shiga-like toxin A subunit | -2.49 | 5.07 | 2.34E-04 |
| STM0285 | STM0285 | inner membrane protein | -1.26 | 6.80 | 1.67E-02 |
| STM0286 | STM0286 | hypothetical protein | -1.33 | 5.32 | 1.09E-02 |
| STM0287 | STM0287 | hypothetical protein | -1.63 | 5.09 | 1.28E-03 |
| STM0288 | STM0288 | hypothetical protein | -1.50 | 5.05 | 3.01E-03 |
| STM0289 | STM0289 | hypothetical protein | -1.65 | 6.62 | 4.82E-04 |
| STM0290 | STM0290 | hypothetical protein | -1.89 | 3.83 | 6.73E-04 |
| STM0291 | STM0291 | RHS-like protein | -1.73 | 5.44 | 2.34E-04 |
| STM0294 | STM0294 | hypothetical protein | -2.18 | 4.50 | 1.24E-02 |
| STM0294.1N | STM0294.1N | hypothetical protein | -3.00 | 2.64 | 6.25E-06 |
| STM0295 | STM0295 | hypothetical protein | -2.20 | 4.14 | 6.32E-03 |
| STM0299 | safA | major pilus subunit SafA | -1.79 | 5.01 | 4.25E-04 |
| STM0300 | safB | fimbrial assembly chaperone | -1.69 | 4.60 | 3.80E-04 |
| STM0301 | safC | fimbrial usher | -1.44 | 7.66 | 3.39E-02 |
| STM0302 | safD | fimbrial subunit | -1.07 | 5.08 | 1.46E-02 |
| STM0303 | ybeJ | xylanase/chitin deacetylase | -1.95 | 5.15 | 1.68E-05 |
| STM0304 | sinR | transcriptional regulator | -1.52 | 5.40 | 4.39E-03 |
| STM0306 | STM0306 | adhesin/invasin protein PagN | -1.64 | 4.88 | 2.65E-04 |
| STM0307 | STM0307 | VirG-like protein | -1.11 | 4.47 | 4.18E-02 |
| STM0309 | fadE | acyl-CoA dehydrogenase | -1.28 | 6.48 | 2.75E-03 |
| STM0320 | phoE | outer membrane phosphoporin protein E | -1.03 | 5.73 | 2.92E-02 |
| STM0325 | STM0325 | IS3 transposase | -1.31 | 5.42 | 1.32E-02 |
| STM0328.s | STM0328.s | permease | -2.68 | 6.18 | 2.91E-05 |
| STM0329 | STM0329 | isopropylmalate isomerase large subunit | -2.55 | 6.06 | 1.51E-05 |
| STM0330 | STM0330 | 3-isopropylmalate isomerase | -1.55 | 5.19 | 1.32E-02 |
| STM0331 | STM0331 | fumarylacetoacetate hydrolase | -1.62 | 5.38 | 2.88E-04 |
| STM0332 | STM0332 | hydrolase/acyltransf erase | -1.75 | 5.56 | 7.91E-05 |
| STM0333 | STM0333 | transcriptional regulator | -1.51 | 5.90 | 3.98E-04 |
| STM0334 | STM0334 | hypothetical protein | -1.45 | 3.84 | 2.43E-03 |
| STM0335 | STM0335 | outer membrane protein | -1.74 | 3.60 | 3.95E-03 |
| STM0336 | stbE | fimbrial chaperone | -1.75 | 4.54 | 2.34E-04 |
| STM0337 | stbD | fimbrial usher | -1.54 | 5.60 | 6.80E-04 |
| STM0338 | stbC | fimbrial usher | -1.57 | 6.81 | 5.65E-04 |
| STM0339 | stbB | fimbrial chaperone | -2.16 | 4.73 | 4.64E-04 |
| STM0340 | stbA | fimbrial major subunit | -2.79 | 3.69 | 2.95E-06 |
| STM0341 | STM0341 | inner membrane protein | -1.66 | 4.77 | 7.43E-04 |
| STM0342 | STM0342 | hypothetical protein | -1.34 | 3.10 | 4.03E-02 |
| STM0343 | STM0343 | hypothetical protein | -1.61 | 6.12 | 1.33E-04 |
| STM0346 | STM0346 | outer membrane protein | -2.28 | 4.54 | 4.51E-04 |
| STM0347 | STM0347 | response regulator | -2.17 | 4.91 | 2.48E-04 |
| STM0348 | STM0348 | inner membrane protein | -2.75 | 3.13 | 1.08E-05 |
| STM0349 | STM0349 | outer membrane lipoprotein | -1.09 | 4.15 | 3.21E-02 |
| STM0350.S | STM0350.S | outer membrane efflux-like protein | -1.81 | 5.31 | 9.25E-05 |
| STM0351 | STM0351 | cation efflux system protein | -2.14 | 6.77 | 1.00E-05 |
| STM0352.S | STM0352.S | cation efflux pump | -2.23 | 5.35 | 6.18E-05 |
| STM0353 | STM0353 | cation transport ATPase | -2.28 | 6.62 | 4.26E-06 |
| STM0354 | STM0354 | transcriptional regulator | -1.10 | 4.46 | 4.04E-02 |
| STM0356 | STM0356 | inner membrane protein | -2.32 | 5.56 | 7.08E-07 |
| STM0360 | STM0360 | cytochrome BD2 subunit I | -2.38 | 5.57 | 5.92E-05 |
| STM0361 | STM0361 | cytochrome BD2 subunit II | -1.55 | 5.62 | 4.50E-04 |
| STM0362 | STM0362 | hypothetical protein | -1.35 | 3.58 | 7.47E-03 |
| STM0364 | foxA | ferrioxamine receptor | -1.46 | 6.68 | 6.78E-04 |
| STM0367 | prpR | prp operon regulator | -2.21 | 5.51 | 3.52E-06 |
| STM0368 | prpB | 2-methylisocitrate lyase | -1.75 | 4.68 | 2.88E-04 |
| STM0369 | prpC | methylcitrate synthase | -2.55 | 5.29 | 4.79E-07 |
| STM0370 | prpD | 2-methylcitrate dehydratase | -2.22 | 5.55 | 2.68E-06 |
| STM0371 | prpE | propionyl-CoA synthetase | -1.86 | 6.23 | 2.59E-05 |
| STM0374 | yaiV | DNA-binding transcriptional regulator | -1.32 | 5.39 | 3.24E-03 |
| STM0381 | STM0381 | inner membrane protein | -1.25 | 4.56 | 8.53E-03 |
| STM0382 | STM0382 | permease | -1.15 | 5.47 | 7.35E-03 |
| STM0403 | yajB | acyl carrier protein phosphodiesterase | -1.12 | 4.93 | 1.62E-02 |
| STM0427 | phnU | 2-aminoethylphosphon ate transporter | -1.50 | 4.57 | 1.28E-03 |
| STM0431 | phnW | 2-aminoethylphosphon ate--pyruvate transaminase | -1.48 | 5.22 | 1.76E-03 |
| STM0432 | phnX | phosphonoacetaldehyd e hydrolase | -1.23 | 4.71 | 1.07E-02 |
| STM0437 | STM0437 | hypothetical protein | -2.06 | 6.18 | 1.22E-04 |
| STM0438 | STM0438 | hypothetical protein | -1.86 | 5.70 | 1.78E-03 |
| STM0440 | cyoD | cytochrome o ubiquinol oxidase subunit IV | -1.14 | 4.63 | 1.67E-02 |
| STM04665 | STM04665 | hypothetical protein | -2.34 | 3.44 | 4.73E-04 |
| STM0469 | rpmE2 | 50S ribosomal protein L31 | -1.65 | 3.60 | 2.67E-03 |
| STM0493 | fsr | transporter | -1.11 | 5.47 | 8.76E-03 |
| STM0497 | STM0497 | hypothetical protein | -1.81 | 5.55 | 7.53E-04 |
| STM04985 | STM04985 | hypothetical protein | -1.22 | 4.18 | 1.76E-02 |
| STM0508 | ybbP | inner membrane protein | -1.09 | 6.66 | 1.10E-02 |
| STM0514 | ybbS | DNA-binding transcriptional activator AllS | -1.50 | 4.82 | 9.97E-04 |
| STM0517 | gcl | glyoxylate carboligase | -1.76 | 3.96 | 2.38E-04 |
| STM0530 | ylbE | hypothetical protein | -2.35 | 5.16 | 2.50E-05 |
| STM0531 | ylbF | hypothetical protein | -2.48 | 4.60 | 1.47E-06 |
| STM0532 | arcC | carbamate kinase | -2.03 | 5.40 | 5.88E-05 |
| STM0539 | STM0539 | inner membrane protein | -1.45 | 4.79 | 7.33E-03 |
| STM0540 | ybcI | membrane-bound metal-dependent hydrolase | -1.18 | 4.95 | 9.04E-03 |
| STM05445 | STM05445 | purine nucleoside phosphorylase | -1.70 | 5.06 | 6.04E-03 |
| STM0550 | fimY | regulatory protein | -1.32 | 5.42 | 1.75E-03 |
| STM0551 | STM0551 | hypothetical protein | -1.01 | 4.12 | 4.69E-02 |
| STM0552 | fimW | fimbrial protein | -1.51 | 4.68 | 9.98E-03 |
| STM05625 | STM05625 | hypothetical protein | -1.05 | 4.46 | 2.45E-02 |
| STM0564 | STM0564 | pyridine nucleotide-disulfide oxidoreductase | -1.63 | 6.37 | 9.87E-05 |
| STM05645 | STM05645 | hypothetical protein | -1.07 | 4.28 | 2.20E-02 |
| STM0566 | STM0566 | inner membrane protein | -1.26 | 5.21 | 8.53E-03 |
| STM0571 | STM0571 | inner membrane protein | -1.07 | 6.57 | 1.32E-02 |
| STM0572 | STM0572 | phosphosugar isomerase | -1.74 | 4.98 | 1.43E-04 |
| STM0573 | STM0573 | inner membrane protein | -1.39 | 4.64 | 2.48E-02 |
| STM0574 | STM0574 | PTS system mannose-specific transporter subunitIID | -2.54 | 4.56 | 1.64E-05 |
| STM0575 | STM0575 | PTS system mannose-specific transporter subunitIIC | -2.07 | 4.01 | 4.38E-05 |
| STM0576 | STM0576 | PTS system mannose-specific transporter subunitIIAB | -2.34 | 3.34 | 7.84E-05 |
| STM0577 | STM0577 | PTS system mannose-specific transporter subunitIIAB | -2.24 | 4.44 | 7.98E-05 |
| STM0583 | ybdK | carboxylate-amine ligase | -1.46 | 5.21 | 7.19E-04 |
| STM0584 | entD | phosphopantetheinylt ransferase component ofenterobactin synthase multienzyme complex | -1.78 | 4.84 | 8.20E-04 |
| STM0585 | fepA | outer membrane receptor FepA | -1.80 | 6.63 | 5.87E-05 |
| STM0586 | fes | enterobactin/ferric enterobactin esterase | -2.21 | 5.28 | 4.15E-05 |
| STM0587 | ybdZ | hypothetical protein | -2.49 | 2.35 | 1.08E-04 |
| STM0588 | entF | enterobactin synthase subunit F | -1.46 | 6.80 | 3.16E-03 |
| STM0591 | fepG | iron-enterobactin transporter permease | -1.16 | 4.77 | 2.35E-02 |
| STM05910 | STM05910 | hypothetical protein | -2.70 | 5.31 | 1.47E-06 |
| STM0592 | fepD | iron-enterobactin transporter membrane protein | -1.60 | 4.84 | 1.56E-03 |
| STM0593 | ybdA | enterobactin exporter EntS | -1.84 | 5.14 | 6.89E-05 |
| STM0594 | fepB | iron-enterobactin transporter periplasmicbinding protein | -1.09 | 4.33 | 2.33E-02 |
| STM0595 | entC | isochorismate synthase | -1.58 | 4.80 | 8.13E-04 |
| STM0596 | entE | enterobactin synthase subunit E | -2.11 | 5.51 | 2.10E-05 |
| STM0597 | entB | 2,3-dihydro-2,3-dihy droxybenzoate synthetase | -1.57 | 4.62 | 1.28E-03 |
| STM0598 | entA | 2,3-dihydroxybenzoat e-2,3-dehydrogenase | -1.44 | 4.83 | 3.86E-03 |
| STM0599 | ybdB | hypothetical protein | -1.49 | 3.73 | 2.27E-03 |
| STM0600 | cstA | carbon starvation protein | -1.33 | 6.62 | 1.80E-03 |
| STM06000 | STM06000 | hypothetical protein | -1.65 | 4.29 | 1.92E-03 |
| STM0610 | STM0610 | anaerobic dehydrogenase component | -2.71 | 4.19 | 1.29E-04 |
| STM0611 | STM0611 | oxidoreductase protein | -1.68 | 6.33 | 6.10E-05 |
| STM0612 | STM0612 | e-S-cluster-containi ng hydrogenase subunit 1 | -1.86 | 4.18 | 1.22E-04 |
| STM0615 | ybdR | dehydrogenase | -1.66 | 5.32 | 2.06E-04 |
| STM0618 | citT | citrate/succinate transport antiport protein | -1.44 | 6.04 | 3.15E-04 |
| STM0620 | citX | 2-(5''-triphosphorib osyl)-3'-dephosphocoenzyme-Asy nthase | -1.46 | 3.21 | 4.74E-03 |
| STM0621 | citF | citrate lyase subunit alpha/citrate-ACPtransferase | -1.86 | 5.36 | 1.88E-03 |
| STM0623 | citD | citrate lyase subunit gamma | -1.54 | 2.77 | 3.90E-03 |
| STM0624 | citC | citrate lyase synthetase | -2.26 | 4.77 | 2.00E-05 |
| STM0625 | dpiB | sensory histidine kinase | -1.76 | 6.17 | 2.97E-05 |
| STM0649.S | STM0649.S | hypothetical protein | -2.45 | 3.56 | 1.23E-05 |
| STM0650 | STM0650 | hypothetical protein | -2.05 | 5.17 | 2.67E-05 |
| STM0651 | STM0651 | 2-keto-3-deoxyglucon ate permease | -1.38 | 5.76 | 1.83E-03 |
| STM0654 | ybeQ | hypothetical protein | -1.20 | 5.56 | 5.72E-03 |
| STM0655 | ybeR | hypothetical protein | -2.54 | 4.88 | 2.68E-06 |
| STM0656 | ybeS | molecular chaperone | -2.19 | 5.61 | 5.13E-06 |
| STM0657 | ybeU | hypothetical protein | -1.62 | 5.08 | 3.41E-03 |
| STM0658 | ybeV | molecular chaperone | -1.60 | 5.76 | 1.56E-04 |
| STM0687 | ybfM | chitoporin | -1.69 | 5.14 | 1.90E-04 |
| STM0689 | citA | citrate-proton symporter | -1.60 | 5.46 | 3.84E-04 |
| STM0690 | citB | citrate utilization protein b | -2.51 | 4.91 | 6.09E-07 |
| STM0691 | STM0691 | tricarballylate dehydrogenase | -1.37 | 5.07 | 2.32E-03 |
| STM0704 | kdpC | potassium-transporti ng ATPase subunit C | -1.69 | 5.14 | 3.23E-04 |
| STM0705 | kdpB | potassium-transporti ng ATPase subunit B | -1.54 | 5.80 | 3.72E-04 |
| STM0706 | kdpA | potassium-transporti ng ATPase subunit A | -2.05 | 5.75 | 1.39E-04 |
| STM0717 | STM0717 | inner membrane protein | -1.37 | 3.73 | 1.06E-02 |
| STM0718 | STM0718 | hypothetical protein | -1.84 | 4.40 | 6.17E-04 |
| STM0719 | STM0719 | UDP-galactopyranose mutase | -1.69 | 4.54 | 3.33E-03 |
| STM0720 | STM0720 | glycosyl transferase family protein | -2.10 | 4.43 | 1.22E-03 |
| STM0721 | STM0721 | glycosyl transferase family protein | -1.90 | 5.13 | 1.45E-02 |
| STM0722 | STM0722 | ABC transporter permease | -2.53 | 4.96 | 1.87E-04 |
| STM0723 | STM0723 | polysaccharide/polyo l phosphate ABC transporterATPase | -2.26 | 4.86 | 3.11E-04 |
| STM0724 | STM0724 | glycosyltransferase | -2.23 | 5.67 | 2.67E-03 |
| STM0725 | STM0725 | glycosyltransferase | -2.74 | 4.25 | 6.36E-04 |
| STM0726 | STM0726 | glycosyl transferase family protein | -2.13 | 5.93 | 9.89E-03 |
| STM0762 | STM0762 | fumarate hydratase | -1.58 | 5.25 | 8.59E-04 |
| STM0763.s | STM0763.s | transcriptional regulator | -1.47 | 4.44 | 2.10E-02 |
| STM0764 | STM0764 | transcriptional regulator | -1.49 | 4.79 | 2.30E-03 |
| STM0765 | STM0765 | cation transporter | -2.66 | 5.35 | 1.58E-05 |
| STM0766 | dcoC | oxaloacetate decarboxylase subunit gamma | -2.05 | 3.37 | 2.93E-03 |
| STM0769 | STM0769 | hypothetical protein | -1.53 | 3.76 | 2.30E-03 |
| STM0770 | STM0770 | iron ABC transporter permease | -1.75 | 6.11 | 3.34E-04 |
| STM0777 | STM0777 | inner membrane protein | -2.26 | 4.70 | 1.64E-05 |
| STM0791 | hutH | histidine ammonia-lyase | -1.40 | 5.60 | 8.70E-04 |
| STM0793 | bioA | adenosylmethionine-- 8-amino-7-oxononanoateaminotra nsferase | -2.03 | 5.06 | 8.53E-06 |
| STM0794 | bioB | biotin synthetase | -1.93 | 5.09 | 1.06E-04 |
| STM0795 | bioF | 8-amino-7-oxononanoa te synthase | -1.36 | 5.12 | 4.29E-03 |
| STM0796 | bioC | biotin biosynthesis protein BioC | -2.11 | 4.05 | 1.18E-04 |
| STM0808 | ybhM | integral membrane protein | -2.07 | 4.36 | 7.87E-05 |
| STM0809 | STM0809 | inner membrane protein | -2.50 | 5.35 | 4.32E-04 |
| STM0810 | STM0810 | inner membrane protein | -2.60 | 4.24 | 5.13E-06 |
| STM0811 | ybhN | hypothetical protein | -1.73 | 5.02 | 9.85E-05 |
| STM0812 | ybhO | cardiolipin synthase 2 | -1.87 | 4.80 | 2.73E-04 |
| STM0813 | ybhP | hypothetical protein | -1.51 | 4.40 | 4.44E-03 |
| STM0836 | ybiR | transporter | -1.02 | 6.07 | 2.47E-02 |
| STM0839 | STM0839 | inner membrane protein | -2.30 | 4.90 | 5.24E-05 |
| STM0843 | pflF | pyruvate formate lyase | -1.38 | 6.04 | 1.34E-03 |
| STM0844 | pflE | pyruvate formate lyase activating enzyme | -1.70 | 4.78 | 4.07E-04 |
| STM0850 | STM0850 | glutathione ABC transporter permease GsiC | -1.07 | 5.37 | 2.27E-02 |
| STM0854 | STM0854 | hypothetical protein | -1.78 | 3.77 | 1.67E-02 |
| STM0855 | STM0855 | electron transfer protein subunit beta | -2.63 | 4.80 | 3.52E-06 |
| STM0856 | STM0856 | electron transfer protein subunit alpha | -2.36 | 4.77 | 1.32E-05 |
| STM0857 | STM0857 | acyl-CoA dehydrogenase | -1.87 | 5.35 | 1.94E-04 |
| STM0858 | STM0858 | dehydrogenase | -2.21 | 5.51 | 1.18E-04 |
| STM0859 | STM0859 | transcriptional regulator | -1.35 | 5.25 | 2.94E-03 |
| STM0860 | STM0860 | inner membrane protein | -2.20 | 4.28 | 2.35E-05 |
| STM0869 | STM0869 | regulatory protein | -1.08 | 4.86 | 1.70E-02 |
| STM0877 | potF | putrescine ABC transporter substrate-bindingprotein | -1.01 | 5.10 | 4.92E-02 |
| STM0878 | potG | putrescine ABC transporter ATP-binding protein | -2.02 | 5.39 | 7.82E-06 |
| STM0879 | potH | putrescine ABC transporter permease | -1.61 | 4.98 | 2.01E-03 |
| STM0885 | STM0885 | inner membrane protein | -1.73 | 3.62 | 2.13E-03 |
| STM0886 | STM0886 | sulfatase | -1.26 | 5.38 | 2.79E-03 |
| STM0907 | STM0907 | chitinase | -1.09 | 5.09 | 9.96E-03 |
| STM0908 | STM0908 | hypothetical protein | -2.16 | 4.05 | 2.15E-05 |
| STM0935 | poxB | pyruvate dehydrogenase | -1.04 | 5.60 | 1.43E-02 |
| STM0947 | STM0947 | integrase | -2.04 | 4.62 | 1.30E-04 |
| STM0951 | STM0951 | hypothetical protein | -1.07 | 4.43 | 1.91E-02 |
| STM0969 | ycaM | amino-acid transporter | -1.36 | 6.09 | 1.17E-03 |
| STM0976 | ycaP | inner membrane protein | -1.75 | 5.11 | 1.08E-04 |
| STM0983 | ycaI | hypothetical protein | -1.34 | 6.33 | 1.53E-03 |
| STM1002 | STM1002 | diaminopropionate ammonia-lyase | -2.23 | 4.93 | 1.18E-05 |
| STM1003 | STM1003 | transcriptional regulator | -2.56 | 5.41 | 3.68E-05 |
| STM1007 | STM1007 | hypothetical protein | -1.23 | 3.84 | 3.58E-02 |
| STM1008.S | STM1008.S | hypothetical protein | -1.09 | 6.70 | 9.48E-03 |
| STM1009 | STM1009 | exodeoxyribonuclease | -1.60 | 6.84 | 6.20E-04 |
| STM1010 | STM1010 | hypothetical protein | -1.74 | 4.41 | 5.27E-04 |
| STM1010.1n | STM1010.1n | hypothetical protein | -1.57 | 4.16 | 1.38E-03 |
| STM1013 | STM1013 | regulatory protein | -1.68 | 2.57 | 2.28E-03 |
| STM1014 | STM1014 | regulatory protein | -2.15 | 3.91 | 4.09E-05 |
| STM1015 | STM1015 | replication protein | -1.76 | 4.05 | 1.21E-03 |
| STM1016 | STM1016 | hypothetical protein | -2.16 | 2.53 | 2.21E-04 |
| STM1017 | STM1017 | hypothetical protein | -1.50 | 2.72 | 8.75E-03 |
| STM1021 | STM1021 | hypothetical protein | -2.42 | 3.97 | 4.73E-06 |
| STM1022 | STM1022 | molecular chaperone | -2.40 | 4.94 | 3.18E-06 |
| STM1025 | STM1025 | hypothetical protein | -1.21 | 4.69 | 1.07E-02 |
| STM1026 | STM1026 | hypothetical protein | -1.30 | 4.68 | 7.97E-03 |
| STM1028 | STM1028 | lysozyme | -1.70 | 4.38 | 4.37E-04 |
| STM1029 | STM1029 | hypothetical protein | -1.34 | 4.75 | 1.69E-02 |
| STM1030 | STM1030 | hypothetical protein | -1.12 | 4.28 | 1.80E-02 |
| STM1031 | STM1031 | hypothetical protein | -1.55 | 6.42 | 2.21E-04 |
| STM1032 | STM1032 | hypothetical protein | -1.33 | 5.52 | 5.01E-03 |
| STM1033 | STM1033 | Clp protease-like protein | -1.20 | 6.73 | 6.29E-03 |
| STM1034 | STM1034 | recombinase A | -1.19 | 2.80 | 3.19E-02 |
| STM1035 | STM1035 | ATP-binding sugar transporter-like protein | -1.75 | 4.49 | 2.23E-04 |
| STM1036 | STM1036 | minor tail protein | -1.55 | 4.26 | 2.14E-03 |
| STM1037 | STM1037 | minor tail protein | -1.88 | 4.29 | 9.06E-04 |
| STM1038 | STM1038 | major tail protein | -1.68 | 5.17 | 1.77E-04 |
| STM1039 | STM1039 | minor tail protein | -1.24 | 3.81 | 1.83E-02 |
| STM1040 | STM1040 | minor tail protein | -1.29 | 3.18 | 2.35E-02 |
| STM1041 | STM1041 | minor tail protein | -1.48 | 7.02 | 2.14E-03 |
| STM1045 | STM1045 | minor tail protein | -1.68 | 4.95 | 2.01E-04 |
| STM1046 | STM1046 | tail assembly protein | -1.29 | 4.66 | 3.97E-03 |
| STM1048 | STM1048 | host specificity protein J | -1.07 | 6.81 | 2.96E-02 |
| STM1048.1N | STM1048.1N | hypothetical protein | -1.39 | 4.82 | 2.37E-03 |
| STM1049 | STM1049 | tail fiber protein | -1.10 | 6.57 | 1.00E-02 |
| STM1050 | STM1050 | tail fiber assembly like-protein | -1.56 | 4.70 | 7.12E-04 |
| STM1051 | sseI | secreted effector protein | -2.10 | 5.01 | 8.44E-05 |
| STM1053 | STM1053 | hypothetical protein | -1.57 | 5.36 | 3.22E-04 |
| STM1056 | STM1056 | MsgA-like protein | -1.98 | 3.13 | 4.30E-03 |
| STM1077 | yccT | hypothetical protein | -1.09 | 4.10 | 2.66E-02 |
| STM1088 | pipB | secreted effector protein | -1.42 | 5.22 | 3.29E-02 |
| STM1090 | pipC | pathogenicity island-encoded protein C | -1.73 | 3.47 | 3.48E-04 |
| STM1091 | sopB | inositol phosphate phosphatase SopB | -2.24 | 5.85 | 2.32E-06 |
| STM1094 | pipD | dipeptidase | -1.05 | 5.84 | 6.64E-03 |
| STM1098 | hpaC | 4-hydroxyphenylaceta te catabolism | -2.02 | 3.26 | 1.07E-04 |
| STM1099 | hpaB | 4-hydroxyphenylaceta te catabolism | -2.20 | 5.28 | 3.09E-05 |
| STM1101 | hpaG | 4-hydroxyphenylaceta te catabolism | -2.40 | 5.07 | 2.47E-06 |
| STM1102 | hpaE | 5-carboxymethyl-2-hy droxymuconate semialdehydedehydrogenase | -1.57 | 5.40 | 9.70E-04 |
| STM1103 | hpaD | 4-hydroxyphenylaceta te catabolism | -1.64 | 4.66 | 1.84E-03 |
| STM1104 | hpaF | 4-hydroxyphenylaceta te catabolism | -2.13 | 3.46 | 6.88E-05 |
| STM1105 | hpaH | 2-oxo-hepta-3-ene-1, 7-dioic acid hydratase | -1.67 | 4.42 | 4.40E-04 |
| STM1106 | hpaI | 4-hydroxyphenylaceta te catabolism | -1.21 | 4.07 | 4.03E-02 |
| STM1107 | hpaX | 4-hydroxyphenylaceta te catabolism | -1.53 | 5.10 | 1.13E-03 |
| STM1108 | hpaA | 4-hydroxyphenylaceta te catabolism | -1.87 | 4.13 | 3.73E-04 |
| STM1109 | STM1109 | hypothetical protein | -1.02 | 4.63 | 2.28E-02 |
| STM1114 | scsB | suppression of copper sensitivity protein | -1.37 | 5.95 | 2.34E-03 |
| STM1115 | scsC | copper sensitivity suppression protein | -1.54 | 3.69 | 1.92E-03 |
| STM1125 | putP | major sodium/proline symporter | -1.23 | 5.65 | 2.67E-03 |
| STM1128 | STM1128 | sodium/glucose cotransporter | -1.51 | 5.76 | 5.64E-04 |
| STM1129 | STM1129 | N-acetylmannosamine- 6-phosphate 2-epimerase | -1.94 | 4.10 | 5.67E-04 |
| STM1130 | STM1130 | N-acetylneuraminic acid mutarotase | -1.54 | 4.86 | 8.94E-03 |
| STM1131 | STM1131 | outer membrane protein | -2.65 | 4.31 | 9.50E-04 |
| STM1132 | STM1132 | sialic acid transporter | -2.03 | 5.92 | 1.39E-04 |
| STM1133 | STM1133 | dehydrogenase | -1.05 | 5.75 | 1.07E-02 |
| STM1139 | csgG | curli operon transcriptional regulator | -1.45 | 4.81 | 2.82E-03 |
| STM1140 | csgF | curli assembly protein CsgF | -3.08 | 3.03 | 1.34E-06 |
| STM1141 | csgE | curli assembly protein CsgE | -3.08 | 2.88 | 1.71E-06 |
| STM1142 | csgD | DNA-binding transcriptional regulator CsgD | -2.26 | 3.45 | 3.74E-04 |
| STM1143 | csgB | curlin minor subunit | -2.10 | 3.07 | 4.25E-04 |
| STM1145 | csgC | autoagglutination protein | -1.40 | 3.46 | 8.79E-03 |
| STM1156 | yceA | hypothetical protein | -1.43 | 4.75 | 5.33E-03 |
| STM1157 | yceI | hypothetical protein | -1.13 | 4.28 | 1.59E-02 |
| STM1158 | STM1158 | inner membrane protein | -2.11 | 3.97 | 2.14E-04 |
| STM1204 | fhuE | ferric-rhodotorulic acid outer membranetransporter | -1.52 | 5.93 | 5.91E-04 |
| STM1224 | sifA | secreted effector protein SifA | -2.13 | 4.87 | 3.23E-04 |
| STM1239 | STM1239 | hypothetical protein | -1.76 | 4.57 | 1.46E-03 |
| STM1240 | envF | envelope lipoprotein | -2.60 | 4.37 | 1.20E-05 |
| STM1252 | STM1252 | hypothetical protein | -1.26 | 5.19 | 2.93E-03 |
| STM1263 | STM1263 | hypothetical protein | -1.38 | 4.61 | 2.67E-03 |
| STM1265 | STM1265 | response regulator | -2.04 | 3.75 | 2.50E-04 |
| STM1269 | STM1269 | chorismate mutase | -1.75 | 3.86 | 4.37E-03 |
| STM1273 | STM1273 | nitric oxide reductase | -1.24 | 4.91 | 6.32E-03 |
| STM1278 | yeaN | amino acid/amine transport protein | -1.29 | 4.90 | 8.87E-03 |
| STM1285 | yeaG | serine protein kinase | -1.01 | 5.88 | 1.06E-02 |
| STM1287 | STM1287 | arylsulfatase regulator | -1.44 | 5.16 | 7.50E-04 |
| STM1304 | astA | arginine succinyltransferase | -1.92 | 4.68 | 4.89E-05 |
| STM1305 | astD | succinylglutamic semialdehyde dehydrogenase | -1.72 | 5.22 | 2.02E-04 |
| STM1306 | astB | succinylarginine dihydrolase | -1.99 | 5.45 | 2.92E-05 |
| STM1307 | astE | succinylglutamate desuccinylase | -1.97 | 4.83 | 7.48E-05 |
| STM1313 | celB | PTS system N,N'-diacetylchitobiose-specif ictransporter subunit IIC | -2.09 | 5.38 | 9.47E-06 |
| STM1314 | celC | PTS system N,N'-diacetylchitobiose-specif ictransporter subunit IIA | -1.11 | 4.14 | 1.92E-02 |
| STM1315 | celD | DNA-binding transcriptional regulator ChbR | -1.21 | 4.36 | 1.53E-02 |
| STM1316 | celF | phospho-beta-glucosi dase/cellobiose-6-phosphatehyd rolase | -1.69 | 5.14 | 9.89E-04 |
| STM1318 | katE | hydroperoxidase II | -1.22 | 6.14 | 1.84E-03 |
| STM1328 | STM1328 | lipid A modifying protein | -1.41 | 5.35 | 1.28E-03 |
| STM1329 | STM1329 | inner membrane protein | -2.29 | 3.23 | 2.06E-04 |
| STM1345 | ydiU | hypothetical protein | -1.07 | 5.61 | 1.14E-02 |
| STM1350 | ydiD | short chain acyl-CoA synthetase | -1.71 | 5.51 | 1.18E-04 |
| STM1352 | ydiS | hypothetical protein | -1.69 | 5.02 | 3.60E-04 |
| STM1353 | ydiR | electron transfer flavoprotein subunit YdiR | -1.33 | 4.80 | 2.75E-03 |
| STM1354 | ydiQ | electron transfer flavoprotein YdiQ | -1.21 | 4.25 | 1.36E-02 |
| STM1355 | ydiP | transcriptional regulator | -1.63 | 4.11 | 1.14E-03 |
| STM1356 | ydiO | acyl-CoA dehydrogenase | -1.36 | 5.20 | 2.43E-03 |
| STM1357.S | ydiF | acetyl-CoA/acetoacet yl-CoA transferase subunitbeta | -2.02 | 5.14 | 2.58E-04 |
| STM1360 | ydiN | transporter | -2.59 | 5.32 | 4.28E-05 |
| STM1361 | ydiM | transporter | -2.34 | 4.93 | 5.17E-05 |
| STM1362 | ydiL | hypothetical protein | -2.75 | 3.24 | 2.34E-05 |
| STM1369 | sufA | iron-sulfur cluster assembly scaffold protein | -1.16 | 3.55 | 2.11E-02 |
| STM1370 | sufB | cysteine desulfurase activator complex subunitSufB | -1.56 | 5.30 | 2.84E-04 |
| STM1371 | sufC | cysteine desulfurase subunit ATPase | -1.46 | 4.46 | 2.30E-03 |
| STM1373 | sufS | bifunctional cysteine desulfurase/selenocysteinelyas e | -1.05 | 5.34 | 2.41E-02 |
| STM1374 | ynhA | cysteine desufuration protein SufE | -1.30 | 4.29 | 1.45E-02 |
| STM1379 | orf48 | amino acid permease | -1.38 | 5.39 | 1.56E-03 |
| STM1381 | orf245 | hypothetical protein | -1.72 | 3.75 | 1.27E-03 |
| STM1382 | orf408 | regulatory protein | -2.55 | 4.80 | 7.15E-06 |
| STM1383 | ttrA | tetrathionate reductase complex subunit A | -1.83 | 6.30 | 2.10E-05 |
| STM1384 | ttrC | tetrathionate reductase complex subunit C | -1.84 | 4.31 | 7.91E-04 |
| STM1385 | ttrB | tetrathionate reductase complex subunit B | -1.81 | 3.89 | 5.91E-04 |
| STM1391 | ssrB | transcriptional activator | -1.25 | 4.27 | 5.83E-03 |
| STM1392 | ssrA | sensor kinase | -1.98 | 6.30 | 2.68E-06 |
| STM1393 | ssaB | secreted effector protein | -2.19 | 3.82 | 1.95E-03 |
| STM1394 | ssaC | outer membrane secretin precursor | -2.14 | 5.06 | 3.85E-04 |
| STM1395 | ssaD | virulence protein | -2.38 | 5.87 | 2.67E-05 |
| STM1396 | ssaE | secretion system effector | -1.88 | 3.72 | 5.40E-04 |
| STM1399 | sscA | secretion system chaperone | -1.90 | 4.12 | 1.01E-04 |
| STM1400 | sseC | translocation machinery protein SseC | -2.31 | 6.16 | 1.85E-06 |
| STM1401 | sseD | translocation machinery protein SseD | -1.80 | 4.25 | 3.57E-04 |
| STM1402 | sseE | secreted effector protein | -1.80 | 3.94 | 5.65E-04 |
| STM1403 | sscB | secretion system chaperone | -1.95 | 3.65 | 8.81E-04 |
| STM1404 | sseF | secreted effector protein | -1.75 | 4.18 | 4.96E-04 |
| STM1405 | sseG | secreted effector protein | -1.47 | 4.13 | 4.94E-03 |
| STM1408 | ssaI | type III secretion system apparatus protein | -2.07 | 2.56 | 4.29E-04 |
| STM1409 | ssaJ | needle complex inner membrane lipoprotein | -2.62 | 4.20 | 2.34E-04 |
| STM1410 | STM1410 | hypothetical protein | -1.94 | 4.65 | 3.90E-03 |
| STM1411 | ssaK | type III secretion system apparatus protein | -2.04 | 3.86 | 3.49E-04 |
| STM1412 | ssaL | type III secretion system apparatus protein | -1.55 | 5.26 | 1.60E-03 |
| STM1413 | ssaM | type III secretion system apparatus protein | -1.54 | 3.26 | 1.09E-02 |
| STM1414 | ssaV | secretion system apparatus protein SsaV | -2.52 | 6.30 | 1.46E-06 |
| STM1415 | ssaN | type III secretion system ATPase | -1.73 | 6.04 | 2.89E-05 |
| STM1416 | ssaO | type III secretion system apparatus protein | -1.52 | 3.39 | 4.12E-03 |
| STM1417 | ssaP | type III secretion system apparatus protein | -1.49 | 4.05 | 2.47E-03 |
| STM1419 | ssaR | type III secretion system protein | -1.38 | 4.48 | 4.42E-03 |
| STM1420 | ssaS | type III secretion system apparatus proteinSsaS | -2.39 | 3.54 | 3.75E-04 |
| STM1421 | ssaT | type III secretion system apparatus protein | -2.80 | 4.68 | 8.06E-06 |
| STM1422 | ssaU | secretion system apparatus protein SsaU | -1.34 | 5.37 | 2.68E-02 |
| STM1472 | STM1472 | hypothetical protein | -1.77 | 5.55 | 1.18E-04 |
| STM1473 | ompN | outer membrane protein N precursor | -2.09 | 4.87 | 1.52E-05 |
| STM1477 | ydgI | amino acid transporter | -1.03 | 5.70 | 5.00E-02 |
| STM1482 | ydgF | multidrug efflux system protein MdtJ | -1.11 | 3.72 | 3.47E-02 |
| STM1492 | STM1492 | ABC transporter permease | -1.83 | 4.18 | 4.55E-04 |
| STM1493 | STM1493 | ABC transporter substrate-binding protein | -2.43 | 4.64 | 6.49E-06 |
| STM1494 | STM1494 | transport system permease component | -2.68 | 4.16 | 6.94E-06 |
| STM1505 | rspA | bifunctional D-altronate/D-mannonatedehydra tase | -2.36 | 4.89 | 1.69E-05 |
| STM1506 | rspB | dehydrogenase | -2.06 | 4.61 | 3.13E-05 |
| STM1514 | ydeJ | competence damage-inducible protein A | -2.02 | 3.78 | 2.27E-04 |
| STM1515 | ydeI | hypothetical protein | -1.18 | 3.87 | 2.06E-02 |
| STM1528 | STM1528 | outer membrane protein | -1.33 | 4.24 | 3.31E-03 |
| STM1530 | STM1530 | outer membrane protein | -1.61 | 5.49 | 4.58E-04 |
| STM1531 | STM1531 | hydrogenase | -1.25 | 3.84 | 2.15E-02 |
| STM1536 | STM1536 | hydrogenase maturation protease | -1.04 | 4.65 | 3.85E-02 |
| STM1537 | STM1537 | Ni/Fe hydrogenase 1 b-type cytochrome subunit | -1.01 | 4.79 | 2.55E-02 |
| STM1538 | STM1538 | hydrogenase-1 large subunit | -1.66 | 5.42 | 1.74E-04 |
| STM1539 | STM1539 | hydrogenase-1 small subunit | -1.49 | 5.33 | 3.32E-04 |
| STM1542 | STM1542 | zinc-binding dehydrogenase | -1.41 | 5.43 | 1.09E-03 |
| STM1543 | STM1543 | transporter | -2.68 | 4.82 | 3.71E-05 |
| STM1544 | pqaA | PhoPQ-regulated protein | -2.13 | 5.60 | 4.64E-04 |
| STM1545 | STM1545 | multidrug efflux protein | -2.04 | 5.30 | 1.97E-05 |
| STM1546 | STM1546 | hypothetical protein | -1.39 | 5.43 | 9.70E-04 |
| STM1551.1n | STM1551.1n | hypothetical protein | -1.73 | 4.01 | 1.44E-03 |
| STM1552 | STM1552 | hypothetical protein | -1.06 | 4.95 | 1.75E-02 |
| STM1554 | STM1554 | coiled-coil protein | -2.53 | 3.95 | 2.75E-05 |
| STM1555 | STM1555 | transcriptional regulator | -3.38 | 3.63 | 2.45E-09 |
| STM1556 | STM1556 | Na+/H+ antiporter | -1.55 | 5.49 | 2.71E-04 |
| STM1557 | STM1557 | aminotransferase | -1.42 | 5.19 | 1.45E-03 |
| STM1559 | STM1559 | glycosyl hydrolase | -1.07 | 5.92 | 7.85E-03 |
| STM1560 | STM1560 | alpha amylase | -1.52 | 5.71 | 5.92E-04 |
| STM1571 | yddG | hypothetical protein | -1.18 | 5.04 | 6.11E-03 |
| STM1576 | narU | nitrate extrusion protein | -1.86 | 5.01 | 1.48E-04 |
| STM1577 | narZ | nitrate reductase 2 subunit alpha | -1.68 | 6.35 | 1.83E-04 |
| STM1578 | narY | nitrate reductase 2 subunit beta | -1.84 | 5.47 | 1.10E-04 |
| STM1579 | narW | nitrate reductase 2 subunit delta | -2.25 | 4.80 | 2.42E-05 |
| STM1580 | narV | nitrate reductase 2 subunit gamma | -1.37 | 4.56 | 3.40E-03 |
| STM1587 | yncD | outer membrane receptor | -1.20 | 5.67 | 3.62E-03 |
| STM1588 | yncC | DNA-binding transcriptional regulator | -1.79 | 3.77 | 5.42E-04 |
| STM1601 | ugtL | hypothetical protein | -1.68 | 3.81 | 1.62E-03 |
| STM1602 | sifB | secreted effector protein | -2.35 | 4.53 | 2.41E-03 |
| STM1612 | STM1612 | cellulase protein | -1.26 | 5.19 | 5.04E-03 |
| STM1614 | STM1614 | PTS system transporter subunit IIC | -1.94 | 5.03 | 3.31E-05 |
| STM1620 | STM1620 | (S)-2-hydroxy-acid oxidase | -2.08 | 5.01 | 3.30E-04 |
| STM1621 | STM1621 | hypothetical protein | -1.72 | 3.46 | 1.94E-03 |
| STM1624 | STM1624 | hypothetical protein | -1.06 | 5.08 | 1.90E-02 |
| STM1625 | ydcI | transcriptional regulator | -1.04 | 4.23 | 3.13E-02 |
| STM1630 | STM1630 | inner membrane protein | -3.10 | 4.53 | 4.56E-04 |
| STM1631 | sseJ | secreted effector protein SseJ | -1.95 | 5.13 | 3.73E-03 |
| STM1633 | STM1633 | extracellular solute-binding protein | -2.72 | 4.39 | 1.74E-04 |
| STM1634 | STM1634 | ABC transporter permease | -3.20 | 3.40 | 1.49E-06 |
| STM1635 | STM1635 | polar amino acid ABC transporter ATPase | -2.43 | 3.85 | 4.40E-04 |
| STM1636 | STM1636 | ABC transporter membrane protein | -2.46 | 4.01 | 3.70E-04 |
| STM1637 | STM1637 | inner membrane protein | -2.56 | 5.84 | 2.95E-06 |
| STM1657 | STM1657 | methyl-accepting chemotaxis protein | -1.08 | 5.27 | 1.56E-02 |
| STM1667 | STM1667 | thiol peroxidase | -1.24 | 3.41 | 4.60E-02 |
| STM1668 | STM1668 | antivirulence protein ZirS | -2.46 | 3.48 | 7.50E-04 |
| STM1669 | STM1669 | invasin-like protein | -2.27 | 5.69 | 2.60E-06 |
| STM1670 | STM1670 | lipoprotein | -1.53 | 3.93 | 4.00E-03 |
| STM1671 | STM1671 | regulatory protein | -2.10 | 4.16 | 7.22E-05 |
| STM1698 | STM1698 | effector kinase SteC | -1.50 | 5.54 | 5.71E-03 |
| STM1701 | yciW | hypothetical protein | -1.47 | 4.89 | 2.42E-03 |
| STM1703 | yciR | RNase II stability modulator | -1.05 | 5.86 | 1.68E-02 |
| STM1723 | trpE | anthranilate synthase component I | -1.25 | 4.65 | 5.48E-03 |
| STM1724 | trpD | bifunctional glutamineamidotransferase/anth ranilate phosphoribosyltransferase | -1.07 | 4.76 | 4.73E-02 |
| STM1729 | yciF | hypothetical protein | -2.65 | 3.29 | 1.90E-05 |
| STM1730 | yciE | hypothetical protein | -2.15 | 3.81 | 1.38E-03 |
| STM1765 | narK | nitrite extrusion protein | -1.81 | 5.50 | 7.04E-05 |
| STM1771 | chaA | calcium/sodium:proto n antiporter | -1.63 | 5.10 | 7.47E-04 |
| STM1797 | ymgE | transglycosylase-ass ociated protein | -1.95 | 3.13 | 1.17E-03 |
| STM1827.S | STM1827.S | diguanylate cyclase/phosphodiesterase | -1.41 | 5.44 | 1.04E-03 |
| STM1836 | STM1836 | penicillin-binding protein 3 | -1.02 | 5.62 | 1.43E-02 |
| STM1843 | STM1843 | transporter | -1.64 | 5.32 | 2.37E-04 |
| STM1853 | pphA | serine/threonine protein phosphatase 1 | -1.36 | 4.36 | 6.84E-03 |
| STM1855 | sopE2 | type III-secreted effector protein | -1.85 | 4.23 | 4.47E-04 |
| STM1859 | STM1859 | hypothetical protein | -1.33 | 2.98 | 1.13E-02 |
| STM1862 | pagO | integral membrane protein | -2.07 | 4.73 | 1.90E-04 |
| STM1863 | STM1863 | inner membrane protein | -1.96 | 2.20 | 1.03E-03 |
| STM1864 | STM1864 | inner membrane protein | -1.62 | 4.04 | 1.15E-03 |
| STM1868 | mig-3 | phage-tail assembly-like protein | -1.26 | 5.01 | 5.16E-03 |
| STM1869 | STM1869 | phage-tail assembly-like protein | -2.27 | 4.16 | 5.04E-05 |
| STM1869A | STM1869A | hypothetical protein | -2.17 | 4.21 | 2.67E-05 |
| STM1870 | STM1870 | hypothetical protein | -2.47 | 5.03 | 1.84E-05 |
| STM1896 | STM1896 | hypothetical protein | -1.65 | 4.68 | 4.71E-03 |
| STM1910 | STM1910 | penicillin-binding protein | -1.15 | 5.78 | 4.82E-03 |
| STM1929 | otsB | trehalose-6-phosphat e phosphatase | -1.37 | 4.90 | 8.75E-03 |
| STM1940 | STM1940 | cell wall-associated hydrolase | -1.20 | 5.76 | 2.84E-03 |
| STM1957 | tnpA_2 | transposase for IS200 | -1.82 | 8.46 | 1.97E-02 |
| STM1982 | rcsA | colanic acid capsular biosynthesis activationprotein A | -1.86 | 4.90 | 1.42E-04 |
| STM1986 | yedP | mannosyl-3-phosphogl ycerate phosphatase | -1.19 | 4.62 | 1.30E-02 |
| STM1990 | yedA | hypothetical protein | -1.08 | 4.97 | 1.24E-02 |
| STM1995 | ompS | porin | -1.13 | 4.92 | 1.90E-02 |
| STM1997 | umuC | DNA polymerase V subunit UmuC | -1.19 | 5.90 | 2.47E-03 |
| STM2007 | STM2007 | hypothetical protein | -2.03 | 5.87 | 1.55E-05 |
| STM2008 | STM2008 | hypothetical protein | -1.80 | 5.26 | 1.82E-04 |
| STM2013 | yeeO | hypothetical protein | -1.11 | 6.02 | 4.59E-03 |
| STM2021 | cbiQ | vitamin B12 biosynthetic protein | -1.50 | 4.74 | 1.41E-03 |
| STM2022 | cbiN | cobalt transport protein CbiN | -1.26 | 2.99 | 3.69E-02 |
| STM2023 | cbiM | cobalt transport protein CbiM | -1.50 | 5.33 | 2.39E-03 |
| STM2024 | cbiL | cobalt-precorrin-2 C(20)-methyltransferase | -1.38 | 4.03 | 3.50E-03 |
| STM2025 | cbiK | vitamin B12 biosynthetic protein | -1.31 | 4.23 | 6.16E-03 |
| STM2026 | cbiJ | cobalt-precorrin-6x reductase | -1.56 | 4.79 | 2.06E-03 |
| STM2027 | cbiH | precorrin-3B C(17)-methyltransferase | -1.59 | 4.47 | 7.22E-04 |
| STM2028 | cbiG | cobalamin biosynthesis protein CbiG | -1.40 | 4.76 | 2.47E-03 |
| STM2029 | cbiF | vitamin B12 biosynthetic protein | -1.32 | 4.48 | 5.11E-03 |
| STM2030 | cbiT | cobalt-precorrin-6Y C(15)-methyltransferase | -1.69 | 3.89 | 4.97E-04 |
| STM2031 | cbiE | cobalt-precorrin-6Y C(5)-methyltransferase | -2.09 | 3.71 | 3.30E-04 |
| STM2032 | cbiD | cobalt-precorrin-6A synthase | -1.61 | 4.74 | 3.42E-04 |
| STM2033 | cbiC | cobalt-precorrin-8X methylmutase | -2.15 | 3.91 | 9.22E-05 |
| STM2034 | cbiB | cobalamin biosynthesis protein | -1.68 | 4.66 | 5.67E-04 |
| STM2035 | cbiA | cobyrinic acid a,c-diamide synthase | -1.33 | 5.86 | 2.53E-03 |
| STM2037 | pduF | propanediol diffusion facilitator | -1.55 | 4.79 | 1.22E-03 |
| STM2038 | pduA | polyhedral body protein PduA | -1.88 | 3.32 | 1.04E-03 |
| STM2039 | pduB | polyhedral body protein | -1.86 | 4.24 | 9.59E-05 |
| STM2040 | pduC | propanediol dehydratase large subunit | -2.19 | 5.72 | 1.47E-06 |
| STM2041 | pduD | propanediol dehydratase medium subunit | -1.56 | 4.19 | 5.25E-03 |
| STM2042 | pduE | propanediol dehydratase small subunit | -1.91 | 3.33 | 5.83E-04 |
| STM2043 | pduG | propanediol dehydratase reactivation protein | -1.61 | 5.20 | 9.12E-04 |
| STM2044 | pduH | propanediol dehydratase reactivation protein | -2.12 | 2.71 | 4.14E-04 |
| STM2045 | pduJ | polyhedral body protein | -1.63 | 2.53 | 3.22E-03 |
| STM2046 | pduK | polyhedral body protein | -2.06 | 3.43 | 7.45E-05 |
| STM2047 | pduL | phosphate propanoyltransferase PduL | -1.93 | 3.30 | 3.88E-04 |
| STM2048 | pduM | propanediol utilization protein PduM | -1.67 | 2.61 | 3.33E-03 |
| STM2050 | pduO | propanediol utilization protein | -1.33 | 4.23 | 9.58E-03 |
| STM2051 | pduP | CoA-dependent propionaldehyde dehydrogenase | -1.81 | 5.37 | 3.22E-05 |
| STM2052 | pduQ | propanol dehydrogenase | -1.42 | 4.35 | 1.40E-03 |
| STM2053 | pduS | polyhedral body protein | -1.45 | 4.48 | 2.09E-03 |
| STM2054 | pduT | polyhedral body protein | -1.65 | 3.57 | 7.76E-04 |
| STM2055 | pduU | polyhedral body protein | -1.45 | 4.13 | 1.22E-02 |
| STM2056 | pduV | propanediol utilization protein | -1.86 | 2.87 | 8.77E-04 |
| STM2057 | pduW | propionate kinase | -1.74 | 4.67 | 2.91E-04 |
| STM2066 | sopA | E3 ubiquitin-protein ligase SopA | -1.17 | 6.57 | 5.44E-03 |
| STM2099 | wcaM | colanic acid biosynthesis protein | -1.90 | 5.95 | 2.01E-04 |
| STM2100 | wcaL | glycosyl transferase family protein | -2.06 | 5.03 | 1.88E-05 |
| STM2101 | wcaK | pyruvyl transferase | -2.02 | 4.99 | 3.90E-05 |
| STM2102 | wzxC | colanic acid exporter | -2.47 | 5.41 | 2.31E-06 |
| STM2103 | wcaJ | UDP-glucose lipid carrier transferase | -2.16 | 4.99 | 3.85E-05 |
| STM2104 | cpsG | phosphomannomutase | -1.63 | 5.20 | 1.32E-02 |
| STM2105.S | manC | mannose-1-phosphate guanylyltransferase | -1.97 | 5.38 | 7.55E-04 |
| STM2106 | wcaI | glycosyl transferase family protein | -2.14 | 4.79 | 8.79E-06 |
| STM2107 | wcaH | GDP-mannose mannosyl hydrolase | -1.59 | 3.68 | 2.45E-02 |
| STM2108 | wcaG | GDP-fucose synthetase | -1.52 | 4.49 | 2.32E-03 |
| STM2109 | gmd | GDP-D-mannose dehydratase | -2.70 | 5.50 | 1.54E-05 |
| STM2110 | wcaF | colanic acid biosynthesis acetyltransferaseWcaF | -2.14 | 3.78 | 1.86E-04 |
| STM2111 | wcaE | glycosyl transferase family protein | -2.85 | 4.07 | 2.68E-06 |
| STM2112 | wcaD | colanic acid biosynthesis protein | -2.18 | 4.97 | 5.00E-04 |
| STM2113 | wcaC | glycosyl transferase family protein | -1.92 | 4.74 | 2.06E-04 |
| STM2114 | wcaB | colanic acid biosynthesis acetyltransferaseWcaB | -1.74 | 3.38 | 1.04E-03 |
| STM2115 | wcaA | glycosyl transferase family protein | -1.72 | 4.47 | 2.24E-04 |
| STM2116 | wzc | tyrosine kinase | -2.03 | 6.04 | 4.21E-06 |
| STM2117 | wzb | tyrosine phosphatase | -2.45 | 3.70 | 1.66E-05 |
| STM2118 | wza | outer membrane polysaccharide export protein | -2.37 | 5.30 | 1.21E-03 |
| STM2125 | yegD | chaperone | -1.58 | 4.93 | 8.92E-04 |
| STM2127 | yegN | multidrug efflux system subunit MdtB | -1.18 | 6.22 | 4.36E-03 |
| STM2128 | yegO | multidrug efflux system subunit MdtC | -1.62 | 6.38 | 1.64E-04 |
| STM2129 | yegB | multidrug efflux system protein MdtE | -1.03 | 5.31 | 2.28E-02 |
| STM2133 | STM2133 | hypothetical protein | -2.58 | 4.62 | 5.55E-05 |
| STM2134 | STM2134 | inner membrane protein | -1.61 | 3.64 | 8.86E-03 |
| STM2135 | STM2135 | inner membrane protein | -1.39 | 5.90 | 6.73E-04 |
| STM2137 | STM2137 | hypothetical protein | -1.95 | 5.02 | 4.84E-04 |
| STM2138 | STM2138 | hypothetical protein | -1.88 | 4.15 | 2.05E-03 |
| STM2139 | STM2139 | inner membrane protein | -1.50 | 3.51 | 3.33E-03 |
| STM2139.2n | STM2139.2n | hypothetical protein | -3.13 | 3.11 | 1.46E-06 |
| STM2142 | yegT | transporter | -1.61 | 5.57 | 1.14E-03 |
| STM2149 | stcD | outer membrane lipoprotein | -2.23 | 5.35 | 4.82E-04 |
| STM2150 | stcC | outer membrane protein | -1.79 | 6.26 | 4.74E-04 |
| STM2151 | stcB | periplasmic chaperone protein | -2.56 | 4.31 | 8.38E-06 |
| STM2152 | stcA | fimbrial-like protein | -1.73 | 4.30 | 5.29E-04 |
| STM2156 | yehR | lipoprotein | -1.97 | 3.75 | 2.33E-03 |
| STM2162 | yehW | proline/glycine betaine ABC transporterpermease | -1.01 | 4.71 | 3.59E-02 |
| STM2163 | yehX | proline/glycine betaine ABC transporter ATPase | -1.32 | 4.89 | 3.74E-03 |
| STM2164 | yehY | proline/glycine betaine ABC transporterpermease | -1.33 | 5.36 | 2.04E-03 |
| STM2169 | yohC | transporter | -1.62 | 3.75 | 6.84E-03 |
| STM2172 | yohG | multidrug resistance outer membrane proteinMdtQ | -1.64 | 5.47 | 3.49E-04 |
| STM2175 | STM2175 | salicylate hydroxylase | -1.09 | 5.42 | 1.70E-02 |
| STM2176 | STM2176 | glutathione S-transferase | -1.96 | 4.77 | 4.85E-05 |
| STM2177 | STM2177 | flutathione S-transferase | -2.05 | 4.19 | 4.80E-05 |
| STM2178 | STM2178 | 1,2-dioxygenase | -2.03 | 4.79 | 2.05E-04 |
| STM2179 | STM2179 | sugar transporter | -2.14 | 4.90 | 5.20E-04 |
| STM2188 | mglC | beta-methylgalactosi de transporter innermembrane protein | -1.96 | 5.28 | 9.36E-05 |
| STM2189 | mglA | galactose/methyl galaxtoside transporterATP-binding protein | -2.00 | 5.16 | 8.06E-04 |
| STM2190 | mglB | galactose-binding transport protein | -1.32 | 5.18 | 1.83E-03 |
| STM2191 | galS | DNA-binding transcriptional regulator GalS | -1.13 | 5.06 | 1.10E-02 |
| STM2197 | STM2197 | phosphoserine phosphatase | -2.54 | 4.31 | 2.95E-06 |
| STM2198 | STM2198 | regulatory protein | -1.97 | 4.99 | 2.08E-03 |
| STM2199 | cirA | colicin I receptor | -1.48 | 6.15 | 4.25E-04 |
| STM2207 | setB | proton efflux pump | -1.10 | 5.76 | 3.54E-02 |
| STM2220 | yejG | hypothetical protein | -1.01 | 5.03 | 2.48E-02 |
| STM2231 | STM2231 | virulence protein | -2.42 | 3.60 | 1.67E-05 |
| STM2232 | oafA | O-antigen acetylase | -1.52 | 5.96 | 4.26E-03 |
| STM2233 | STM2233 | hypothetical protein | -2.39 | 4.21 | 1.50E-04 |
| STM2234 | STM2234 | phage tail fiber assembly protein | -1.97 | 4.27 | 2.84E-04 |
| STM2235 | STM2235 | hypothetical protein | -1.69 | 6.18 | 4.39E-05 |
| STM2236 | STM2236 | hypothetical protein | -2.09 | 4.22 | 6.52E-05 |
| STM2237 | STM2237 | inner membrane protein | -2.33 | 2.87 | 5.08E-05 |
| STM2239 | STM2239 | phage antiterminator | -1.64 | 4.56 | 2.54E-03 |
| STM2240 | STM2240 | hypothetical protein | -1.53 | 7.14 | 2.49E-03 |
| STM2242 | STM2242 | phage tail fiber protein | -2.73 | 3.08 | 7.08E-06 |
| STM2243 | STM2243 | tail fiber protein of phage | -2.01 | 4.34 | 5.31E-05 |
| STM2255 | napC | cytochrome c-type protein NapC | -1.57 | 4.78 | 5.83E-04 |
| STM2256 | napB | citrate reductase cytochrome c-type subunit | -1.15 | 5.24 | 1.87E-02 |
| STM2257 | napH | quinol dehydrogenase membrane component | -1.80 | 5.80 | 2.06E-04 |
| STM2260 | napD | assembly protein for periplasmic nitratereductase | -1.30 | 3.29 | 1.55E-02 |
| STM2261 | napF | ferredoxin-type protein | -1.11 | 4.20 | 3.30E-02 |
| STM2263 | yojI | multidrug transporter membraneprotein/ATP-binding component | -1.46 | 5.82 | 4.07E-04 |
| STM2273 | STM2273 | dehydratase | -1.95 | 5.91 | 2.02E-04 |
| STM2274 | STM2274 | permease | -2.59 | 5.43 | 2.67E-05 |
| STM2275 | STM2275 | regulatory protein | -1.83 | 4.39 | 4.58E-04 |
| STM2280 | STM2280 | permease | -1.13 | 5.70 | 7.45E-03 |
| STM2287 | sseL | deubiquitinase SseL | -1.97 | 4.85 | 6.90E-05 |
| STM2290 | yfaV | transporter | -1.23 | 5.53 | 5.07E-03 |
| STM2291 | yfaW | galactonate dehydratase | -1.12 | 6.38 | 6.33E-03 |
| STM2292 | yfaX | transcriptional regulator | -1.39 | 4.74 | 2.25E-03 |
| STM2315 | yfbK | hypothetical protein | -1.22 | 6.12 | 3.42E-03 |
| STM2329 | STM2329 | hypothetical protein | -2.53 | 3.28 | 1.43E-05 |
| STM2340 | STM2340 | transketolase | -1.28 | 5.04 | 4.28E-03 |
| STM2341 | STM2341 | transketolase | -1.76 | 4.63 | 3.10E-04 |
| STM2343 | STM2343 | hypothetical protein | -1.90 | 3.39 | 1.49E-03 |
| STM2344 | STM2344 | PTS system transporter subunit IIA | -1.80 | 4.41 | 6.11E-03 |
| STM2358 | STM2358 | hypothetical protein | -1.47 | 5.06 | 1.79E-03 |
| STM2359 | STM2359 | amino acid transporter | -1.71 | 5.44 | 1.22E-04 |
| STM2360 | STM2360 | diaminopimelate decarboxylase | -1.80 | 5.57 | 4.61E-05 |
| STM2373 | STM2373 | hypothetical protein | -2.21 | 3.57 | 5.04E-05 |
| STM2375 | STM2375 | hypothetical protein | -1.75 | 3.02 | 5.11E-03 |
| STM2376 | STM2376 | hypothetical protein | -1.55 | 4.50 | 2.08E-03 |
| STM2377 | STM2377 | inner membrane protein | -1.68 | 5.24 | 2.07E-02 |
| STM2389 | fadI | 3-ketoacyl-CoA thiolase | -1.76 | 5.51 | 3.55E-03 |
| STM2393 | yfdC | hypothetical protein | -1.73 | 5.31 | 3.96E-04 |
| STM2395 | pgtE | outer membrane protease | -1.91 | 5.28 | 3.08E-05 |
| STM2396 | pgtA | activator | -1.29 | 6.45 | 1.61E-03 |
| STM2398 | pgtC | phosphoglycerate transport regulatory proteinprecursor | -1.52 | 5.23 | 6.73E-04 |
| STM2399 | pgtP | phosphoglycerate transporter | -2.29 | 5.62 | 6.42E-07 |
| STM2400 | STM2400 | inner membrane protein | -1.35 | 3.93 | 4.91E-03 |
| STM2420 | xapR | DNA-binding transcriptional activator XapR | -1.94 | 5.82 | 2.36E-04 |
| STM2421 | xapB | xanthosine permease | -2.11 | 5.81 | 1.25E-05 |
| STM2423 | yfeN | hypothetical protein | -2.43 | 5.13 | 2.35E-05 |
| STM2441 | cysA | sulfate/thiosulfate transporter subunit | -1.11 | 5.45 | 8.37E-03 |
| STM2442 | cysW | sulfate/thiosulfate transporter permeasesubunit | -2.20 | 4.91 | 3.59E-06 |
| STM2444 | cysP | thiosulfate transporter subunit | -1.63 | 4.87 | 8.13E-04 |
| STM2455 | eutK | carboxysome structural protein | -1.30 | 4.59 | 4.88E-03 |
| STM2456 | eutL | carboxysome structural protein | -1.18 | 4.46 | 1.81E-02 |
| STM2458 | eutB | ethanolamine ammonia-lyase heavy chain | -1.76 | 5.99 | 3.85E-05 |
| STM2459 | eutA | reactivating factor for ethanolamine ammonialyase | -1.28 | 4.90 | 8.29E-03 |
| STM2460 | eutH | transporter | -1.56 | 5.70 | 5.65E-04 |
| STM2462 | eutJ | ethanolamine utilization protein | -1.35 | 4.65 | 1.14E-02 |
| STM2463 | eutE | aldehyde oxidoreductase | -1.55 | 5.24 | 7.96E-04 |
| STM2464 | eutN | ethanolamine utilization protein EutN | -1.29 | 3.59 | 1.95E-02 |
| STM2466 | eutD | phosphotransacetylas e | -1.28 | 4.52 | 3.81E-02 |
| STM2467 | eutT | cobalamin adenosyltransferase | -1.10 | 4.21 | 2.49E-02 |
| STM2468 | eutQ | ethanolamine utilization protein | -1.82 | 4.46 | 2.43E-04 |
| STM2469 | eutP | ethanolamine utilization protein | -1.61 | 3.50 | 4.51E-03 |
| STM2470 | eutS | carboxysome structural protein | -2.13 | 2.78 | 3.13E-04 |
| STM2473 | talA | transaldolase A | -1.35 | 5.44 | 1.62E-03 |
| STM2476 | ypfG | hypothetical protein | -1.02 | 5.59 | 1.22E-02 |
| STM2503 | STM2503 | diguanylate cyclase | -1.26 | 6.41 | 2.59E-03 |
| STM2508 | STM2508 | hypothetical protein | -1.30 | 3.61 | 1.58E-02 |
| STM2509 | STM2509 | transposase | -1.46 | 4.21 | 3.45E-03 |
| STM2513 | shdA | AIDA autotransporter-like protein | -1.40 | 7.88 | 4.34E-02 |
| STM2516 | sinI | outer membrane protein | -2.07 | 5.38 | 1.97E-05 |
| STM2517 | sinH | intimin-like protein | -2.53 | 6.07 | 1.22E-06 |
| STM2556 | hmpA | nitric oxide dioxygenase | -1.12 | 5.40 | 5.65E-03 |
| STM2566 | STM2566 | hypothetical protein | -2.14 | 3.40 | 1.18E-04 |
| STM2570 | STM2570 | PTS system transporter subunit IIB | -1.12 | 6.29 | 5.01E-03 |
| STM2573 | STM2573 | 2-dehydropantoate 2-reductase | -1.30 | 5.61 | 1.86E-03 |
| STM2574 | STM2574 | permease | -2.60 | 6.05 | 9.69E-05 |
| STM2575 | STM2575 | transcriptional regulator | -2.00 | 5.19 | 1.07E-04 |
| STM2584 | gogB | hypothetical protein | -1.15 | 6.45 | 2.73E-02 |
| STM2586 | STM2586 | phage tail assembly-like protein | -1.37 | 4.47 | 8.53E-03 |
| STM2587 | STM2587 | phage tail assembly-like protein | -1.52 | 5.22 | 1.43E-03 |
| STM2589 | STM2589 | host specificity protein-J-like | -1.56 | 7.54 | 1.20E-02 |
| STM2591 | STM2591 | tail assembly protein K-like | -1.51 | 4.91 | 1.67E-03 |
| STM2592 | STM2592 | phage tail component L-like protein | -1.07 | 5.83 | 8.20E-03 |
| STM2593 | STM2593 | phage tail component M-like protein | -1.20 | 4.19 | 1.23E-02 |
| STM2600 | STM2600 | minor tail protein Z-like | -1.07 | 6.15 | 5.50E-03 |
| STM2601 | STM2601 | minor capsid protein FII | -1.05 | 4.82 | 1.35E-02 |
| STM2602 | STM2602 | DNA packaging-like protein | -1.21 | 4.57 | 1.07E-02 |
| STM2606 | STM2606 | head-tail preconnector-like protein | -1.02 | 7.01 | 2.98E-02 |
| STM2609 | STM2609 | DNA packaging-like protein | -1.48 | 4.75 | 8.43E-04 |
| STM2611.S | STM2611.S | endopeptidase-like protein | -1.33 | 4.24 | 5.11E-03 |
| STM2614 | STM2614 | hypothetical protein | -1.73 | 3.33 | 3.84E-03 |
| STM2623 | STM2623 | hypothetical protein | -1.48 | 2.71 | 8.46E-03 |
| STM2624 | STM2624 | hypothetical protein | -1.84 | 2.54 | 2.40E-03 |
| STM2625 | STM2625 | replication protein | -1.93 | 4.08 | 1.05E-03 |
| STM2626 | STM2626 | replication protein 15-like | -2.04 | 4.04 | 3.10E-05 |
| STM2627 | STM2627 | cI-like protein | -1.94 | 2.59 | 6.99E-04 |
| STM2629 | STM2629 | hypothetical protein | -1.26 | 3.04 | 2.50E-02 |
| STM2632 | STM2632 | exodeoxyribonuclease VIII-like protein | -1.77 | 6.79 | 1.29E-04 |
| STM2654 | kgtP | alpha-ketoglutarate transporter | -1.67 | 6.20 | 6.18E-05 |
| STM2655 | STM2655 | hypothetical protein | -1.80 | 3.98 | 1.56E-04 |
| STM2691 | STM2691 | ABC transporter ATP-binding protein | -2.29 | 6.34 | 6.10E-05 |
| STM2692 | STM2692 | HlyD family secretion protein | -1.41 | 5.66 | 1.09E-03 |
| STM2705 | STM2705 | hypothetical protein | -1.57 | 4.19 | 2.32E-03 |
| STM2706 | STM2706 | phage tail-like protein | -1.31 | 4.44 | 3.78E-03 |
| STM2740 | STM2740 | integrase-like protein | -1.51 | 5.64 | 1.64E-03 |
| STM2741 | STM2741 | hypothetical protein | -2.23 | 5.12 | 8.23E-06 |
| STM2745 | STM2745 | inner membrane protein | -1.11 | 6.22 | 4.76E-02 |
| STM2746 | STM2746 | ATPase | -1.98 | 4.96 | 3.48E-03 |
| STM2747 | STM2747 | hypothetical protein | -2.25 | 4.63 | 1.09E-03 |
| STM2753 | STM2753 | dehydrogenase | -1.11 | 6.02 | 1.02E-02 |
| STM2754 | STM2754 | hexulose 6 phosphate synthase | -2.44 | 5.79 | 7.95E-04 |
| STM2755 | STM2755 | hexulose 6 phosphate synthase | -2.66 | 5.47 | 2.05E-06 |
| STM2756 | STM2756 | sugar phosphate aminotransferase | -2.16 | 4.50 | 2.20E-04 |
| STM2757 | STM2757 | hypothetical protein | -2.31 | 5.26 | 3.49E-05 |
| STM2758 | STM2758 | PTS system transporter subunit IIBC | -2.33 | 6.05 | 3.42E-06 |
| STM2759 | STM2759 | dipeptide/oligopepti de/nickel ABC-type ABCtransporter substrate-binding protein | -1.58 | 5.91 | 1.90E-04 |
| STM2760 | STM2760 | integrase | -2.02 | 4.23 | 1.09E-04 |
| STM2761 | STM2761 | inner membrane protein | -2.07 | 5.78 | 7.50E-04 |
| STM2762 | STM2762 | inner membrane protein | -1.72 | 5.38 | 2.73E-03 |
| STM2770 | fljA | phase-1 flagellin repressor | -1.63 | 4.16 | 2.08E-03 |
| STM2772 | hin | DNA-invertase Hin | -1.36 | 4.56 | 3.78E-03 |
| STM2773 | iroB | glycosyl transferase family protein | -1.51 | 5.78 | 4.83E-04 |
| STM2774 | iroC | ABC transporter ATP-binding protein | -1.56 | 7.14 | 3.60E-03 |
| STM2775 | iroD | enterochelin esterase-like protein | -1.84 | 5.31 | 1.74E-03 |
| STM2776 | iroE | hydrolase | -1.94 | 5.00 | 2.67E-05 |
| STM2777 | iroN | outer membrane receptor FepA | -1.54 | 6.36 | 4.40E-04 |
| STM2780 | pipB2 | secreted effector protein PipB2 | -1.37 | 5.72 | 1.14E-03 |
| STM2785 | tctD | regulatory protein TctD | -1.67 | 5.43 | 1.53E-04 |
| STM2786 | STM2786 | tricarboxylic transport | -1.68 | 4.72 | 4.37E-04 |
| STM2787 | STM2787 | tricarboxylic transport | -1.50 | 3.09 | 6.89E-03 |
| STM2789 | STM2789 | hypothetical protein | -2.61 | 5.27 | 2.18E-07 |
| STM2790 | ygaF | hydroxyglutarate oxidase | -1.98 | 5.53 | 4.05E-05 |
| STM2791 | gabD | succinate-semialdehy de dehydrogenase I | -1.94 | 5.34 | 1.01E-04 |
| STM2793 | gabP | gamma-aminobutyrate transporter | -2.22 | 5.37 | 2.64E-05 |
| STM2794 | ygaE | DNA-binding transcriptional regulator CsiR | -1.68 | 4.37 | 1.01E-03 |
| STM2795 | ygaU | LysM domain/BON superfamily protein | -1.78 | 4.78 | 1.35E-03 |
| STM2804 | STM2804 | hypothetical protein | -1.09 | 3.45 | 3.47E-02 |
| STM2804.1n | STM2804.1n | hypothetical protein | -1.37 | 2.92 | 2.14E-02 |
| STM2806 | nrdI | ribonucleotide reductase stimulatory protein | -2.63 | 3.67 | 1.46E-06 |
| STM2807 | nrdE | ribonucleotide-dipho sphate reductase subunitalpha | -1.58 | 6.05 | 9.55E-05 |
| STM2808 | nrdF | ribonucleotide-dipho sphate reductase subunitbeta | -1.30 | 5.52 | 2.72E-03 |
| STM2809 | proV | glycine betaine transporter ATP-binding subunit | -1.17 | 5.74 | 3.13E-02 |
| STM2810 | proW | glycine betaine transporter membrane protein | -1.23 | 5.24 | 5.02E-03 |
| STM2811 | proX | glycine betaine transporter periplasmic subunit | -1.76 | 5.52 | 1.22E-04 |
| STM2816 | STM2816 | glycoporin | -2.00 | 6.01 | 1.55E-05 |
| STM2832 | srlA | PTS system glucitol/sorbitol-specifictran sporter subunit IIC | -2.07 | 4.54 | 1.73E-03 |
| STM2833 | srlE | PTS system glucitol/sorbitol-specifictran sporter subunit IICB | -1.74 | 5.07 | 1.44E-04 |
| STM2834 | slrB | PTS system glucitol/sorbitol-specifictran sporter subunit IIA | -1.70 | 3.45 | 1.63E-03 |
| STM2836 | gutM | DNA-binding transcriptional activator GutM | -1.38 | 3.09 | 9.74E-03 |
| STM2840 | STM2840 | anaerobic nitric oxide reductaseflavorubredoxin | -1.97 | 5.90 | 7.50E-06 |
| STM2861 | sitA | periplasmic binding protein | -1.63 | 4.85 | 3.58E-04 |
| STM2862 | sitB | ATP-binding protein | -1.45 | 5.21 | 8.92E-04 |
| STM2863 | sitC | permease | -1.80 | 5.48 | 3.14E-05 |
| STM2871 | prgK | needle complex inner membrane lipoprotein | -1.07 | 6.11 | 1.46E-02 |
| STM2873 | prgI | needle complex major subunit | -1.13 | 4.71 | 4.96E-02 |
| STM2874 | prgH | needle complex inner membrane protein | -1.48 | 6.78 | 1.47E-03 |
| STM2876 | hilA | invasion protein regulator | -1.89 | 6.55 | 7.58E-05 |
| STM2877 | iagB | invasion protein precursor | -1.11 | 4.43 | 2.68E-02 |
| STM2878 | sptP | protein tyrosine phosphatase/GTPase activatingprotein | -1.10 | 7.17 | 2.88E-02 |
| STM2879 | sicP | secretion chaperone | -1.09 | 5.36 | 7.66E-03 |
| STM2881 | iacP | acyl carrier protein | -1.50 | 4.28 | 1.62E-03 |
| STM2882 | sipA | cell invasion protein SipA | -1.68 | 7.74 | 1.10E-02 |
| STM2883 | sipD | cell invasion protein SipD | -1.59 | 6.11 | 2.98E-04 |
| STM2885 | sipB | cell invasion protein SipB | -1.35 | 7.75 | 4.18E-02 |
| STM2886 | sicA | secretion chaperone SicA | -1.38 | 5.30 | 2.72E-03 |
| STM2887 | spaS | surface presentation of antigens protein SpaS | -2.29 | 5.61 | 1.37E-04 |
| STM2888 | spaR | needle complex export protein | -1.90 | 5.05 | 1.97E-05 |
| STM2889 | spaQ | needle complex export protein | -1.50 | 3.88 | 1.87E-02 |
| STM2890 | spaP | surface presentation of antigens protein SpaP | -2.06 | 5.77 | 1.22E-06 |
| STM2891 | spaO | surface presentation of antigens protein SpaO | -1.51 | 6.50 | 8.13E-04 |
| STM2892 | invJ | needle length control protein | -2.03 | 6.75 | 2.24E-05 |
| STM2893 | invI | needle complex assembly protein | -2.33 | 5.74 | 4.90E-06 |
| STM2894 | invC | ATP synthase SpaL | -1.09 | 6.54 | 1.00E-02 |
| STM2895 | invB | secretion chaperone | -1.64 | 5.70 | 3.30E-04 |
| STM2896 | invA | needle complex export protein | -1.81 | 7.28 | 6.38E-04 |
| STM2897 | invE | invasion protein | -1.80 | 6.42 | 1.64E-05 |
| STM2898 | invG | outer membrane secretin InvG | -1.88 | 7.15 | 4.32E-04 |
| STM2899 | invF | invasion regulatory protein | -1.25 | 5.95 | 2.43E-03 |
| STM2900 | invH | needle complex outer membrane lipoproteinprecursor | -1.51 | 5.62 | 6.93E-04 |
| STM2907 | pphB | serine/threonine-spe cific protein phosphatase 2 | -1.02 | 5.27 | 2.98E-02 |
| STM2908 | STM2908 | hypothetical protein | -1.70 | 4.91 | 5.34E-04 |
| STM2911 | STM2911 | permease | -1.64 | 5.85 | 2.85E-04 |
| STM2913 | STM2913 | permease | -1.29 | 6.42 | 2.54E-03 |
| STM2914 | STM2914 | nucleoside-diphospha te-sugar epimerase | -2.13 | 5.25 | 6.59E-04 |
| STM2915 | ygbM | hypothetical protein | -2.51 | 4.97 | 4.62E-06 |
| STM2916 | ygbL | aldolase | -2.53 | 4.21 | 2.05E-06 |
| STM2917 | ygbK | tRNA synthase | -1.79 | 4.98 | 2.10E-03 |
| STM2918 | ygbJ | 3-hydroxyisobutyrate dehydrogenase | -2.19 | 4.75 | 1.75E-04 |
| STM2921 | STM2921 | 3-octaprenyl-4-hydro xybenzoate carboxy-lyase | -1.64 | 4.57 | 5.45E-03 |
| STM2922 | STM2922 | 3-polyprenyl-4-hydro xybenzoate decarboxylase | -1.78 | 6.06 | 1.52E-05 |
| STM2933 | cysC | adenylylsulfate kinase | -1.27 | 4.83 | 7.94E-03 |
| STM2935 | cysD | sulfate adenylyltransferase subunit 2 | -1.99 | 5.04 | 2.50E-05 |
| STM2937 | ygbF | hypothetical protein | -1.74 | 5.03 | 9.00E-04 |
| STM2938 | STM2938 | hypothetical protein | -1.29 | 5.36 | 2.35E-03 |
| STM2939 | ygcH | hypothetical protein | -1.14 | 4.95 | 1.09E-02 |
| STM2942 | STM2942 | transposase | -1.34 | 5.01 | 2.99E-03 |
| STM2943 | STM2943 | hypothetical protein | -1.76 | 6.71 | 1.44E-04 |
| STM2944 | ygcB | helicase | -2.00 | 6.85 | 5.40E-05 |
| STM2945 | sopD | secreted effector protein SopD | -2.24 | 5.42 | 1.94E-04 |
| STM2946 | cysH | phosphoadenosine phosphosulfate reductase | -1.45 | 4.26 | 4.38E-03 |
| STM2947 | cysI | sulfite reductase subunit beta | -1.43 | 5.89 | 1.91E-03 |
| STM2948 | cysJ | sulfite reductase subunit alpha | -2.09 | 6.15 | 6.68E-06 |
| STM2950 | STM2950 | metal-dependent hydrolase | -1.46 | 6.04 | 2.38E-04 |
| STM2961 | ygcY | D-glucarate dehydratase | -1.30 | 5.53 | 1.26E-03 |
| STM2962 | gudT | D-glucarate permease | -1.87 | 6.09 | 7.77E-06 |
| STM2973 | fucO | L-1,2-propanediol oxidoreductase | -1.31 | 6.06 | 3.99E-03 |
| STM2974 | fucA | L-fuculose phosphate aldolase | -2.07 | 4.76 | 4.76E-05 |
| STM2976 | fucI | L-fucose isomerase | -2.13 | 5.84 | 5.13E-06 |
| STM2977 | fucK | L-fuculokinase | -1.57 | 5.16 | 4.24E-04 |
| STM2978 | fucU | L-fucose-binding protein | -1.32 | 3.75 | 7.35E-03 |
| STM2992 | argA | N-acetylglutamate synthase | -1.18 | 6.08 | 2.93E-03 |
| STM2997 | ppdC | hypothetical protein | -1.84 | 4.59 | 1.03E-04 |
| STM2998 | ygdB | hypothetical protein | -1.33 | 4.79 | 4.38E-03 |
| STM2999 | ppdB | hypothetical protein | -1.49 | 4.72 | 4.00E-03 |
| STM3000 | ppdA | hypothetical protein | -2.05 | 4.08 | 4.58E-04 |
| STM3004.1n | STM3004.1n | hypothetical protein | -3.51 | 2.82 | 5.95E-08 |
| STM3013 | lysA | diaminopimelate decarboxylase | -1.20 | 6.02 | 2.92E-03 |
| STM3016 | araE | L-arabinose/proton symport protein | -1.89 | 5.96 | 8.93E-06 |
| STM3017 | kduD | 2-deoxy-D-gluconate 3-dehydrogenase | -1.41 | 5.72 | 1.02E-03 |
| STM3018 | kduI | 5-keto-4-deoxyuronat e isomerase | -1.40 | 5.22 | 1.38E-03 |
| STM3019 | yqeF | acetyl-CoA acetyltransferase | -1.75 | 6.02 | 2.51E-04 |
| STM3023 | yohL | hypothetical protein | -1.07 | 4.25 | 2.06E-02 |
| STM3024 | yohM | nickel/cobalt efflux protein RcnA | -1.13 | 5.66 | 1.26E-02 |
| STM3025 | STM3025 | hypothetical protein | -1.38 | 5.58 | 3.31E-03 |
| STM3025.1N | STM3025.1N | hypothetical protein | -2.09 | 4.05 | 5.78E-05 |
| STM3026 | STM3026 | outer membrane protein | -2.05 | 5.69 | 5.68E-04 |
| STM3027 | stdC | fimbrial chaperone | -2.51 | 4.68 | 7.12E-07 |
| STM3028 | stdB | outer membrane usher protein | -1.88 | 6.21 | 6.89E-05 |
| STM3029.S | stdA | fimbrial-like protein | -2.36 | 5.09 | 3.29E-05 |
| STM3030 | STM3030 | hypothetical protein | -1.71 | 5.27 | 1.42E-04 |
| STM3031 | STM3031 | Ail/OmpX-like protein | -1.81 | 4.59 | 1.72E-04 |
| STM3052 | STM3052 | outer membrane protein | -1.92 | 5.77 | 2.12E-03 |
| STM3066 | yggA | arginine exporter protein | -1.74 | 5.43 | 7.60E-05 |
| STM3079.S | STM3079.S | hydrolase/acyltransf erase | -2.20 | 5.40 | 1.92E-06 |
| STM3080 | STM3080 | mannitol dehydrogenase | -1.61 | 4.31 | 2.18E-02 |
| STM3081 | STM3081 | malate/L-lactate dehydrogenase | -2.13 | 5.16 | 2.15E-05 |
| STM3082 | STM3082 | zinc-binding dehydrogenase | -1.57 | 5.34 | 6.25E-04 |
| STM3083 | STM3083 | mannitol dehydrogenase | -2.12 | 5.83 | 3.40E-06 |
| STM3085 | STM3085 | outer membrane lipoprotein | -1.82 | 4.78 | 3.96E-04 |
| STM3089 | yqgD | inner membrane protein | -2.63 | 3.22 | 6.68E-06 |
| STM3099 | yggR | twitching motility protein | -2.00 | 5.31 | 2.10E-05 |
| STM3105 | yggM | hypothetical protein | -2.07 | 5.50 | 2.59E-05 |
| STM3115 | yqgA | inner membrane protein | -1.19 | 5.32 | 5.38E-03 |
| STM3118 | STM3118 | acetyl-CoA hydrolase | -1.66 | 6.36 | 1.29E-04 |
| STM3119 | STM3119 | monoamine oxidase | -1.28 | 4.73 | 8.92E-03 |
| STM3121 | STM3121 | transcriptional regulator | -1.18 | 5.86 | 3.64E-03 |
| STM3123 | STM3123 | arylsulfatase regulator | -2.15 | 5.35 | 3.54E-06 |
| STM3124 | STM3124 | response regulator | -1.80 | 5.06 | 2.48E-04 |
| STM3125 | STM3125 | hypothetical protein | -1.70 | 5.00 | 1.53E-04 |
| STM3126 | STM3126 | amino acid transporter | -1.92 | 6.78 | 4.76E-05 |
| STM3128 | STM3128 | oxidoreductase | -1.03 | 6.02 | 1.78E-02 |
| STM3129 | STM3129 | NAD-dependent aldehyde dehydrogenase | -1.22 | 6.12 | 2.30E-03 |
| STM3132 | STM3132 | xylanase/chitin deacetylase | -2.14 | 5.60 | 2.95E-06 |
| STM3133 | STM3133 | amidohydrolase | -3.00 | 5.02 | 6.77E-05 |
| STM3142 | STM3142 | periplasmic ferrichrome-binding protein | -1.21 | 5.86 | 8.15E-03 |
| STM3166.S | STM3166.S | cation transporter | -2.41 | 5.51 | 5.67E-05 |
| STM3169 | STM3169 | periplasmic dicarboxylate-binding protein | -2.58 | 5.02 | 1.34E-06 |
| STM3170 | STM3170 | inner membrane protein | -2.89 | 4.18 | 3.61E-07 |
| STM3193 | STM3193 | disulfide bond formation protein DsbL | -2.28 | 4.92 | 1.52E-05 |
| STM3194 | STM3194 | disulfide oxidoreductase | -1.37 | 5.25 | 1.30E-03 |
| STM3198 | STM3198 | inner membrane protein | -1.79 | 4.10 | 1.03E-03 |
| STM3199 | yqiK | hypothetical protein | -1.37 | 6.99 | 3.63E-03 |
| STM3217 | aer | aerotaxis sensor receptor | -1.27 | 6.57 | 6.44E-03 |
| STM3218 | oat | putrescine--2-oxoglu tarate aminotransferase | -2.16 | 6.41 | 2.68E-06 |
| STM3219 | fadH | 2,4-dienoyl-CoA reductase | -2.07 | 6.40 | 3.26E-05 |
| STM3222 | ygjQ | integral membrane protein | -1.27 | 4.80 | 4.80E-03 |
| STM3240 | tdcG | L-serine deaminase | -1.04 | 5.96 | 1.06E-02 |
| STM3241 | tdcE | pyruvate formate-lyase 4/2-ketobutyrateformate-lyase | -1.28 | 6.75 | 7.14E-03 |
| STM3242 | tdcD | propionate/acetate kinase | -2.18 | 5.74 | 2.98E-05 |
| STM3243 | tdcC | threonine/serine transporter TdcC | -1.58 | 6.51 | 3.06E-04 |
| STM3244 | tdcB | threonine dehydratase | -1.70 | 5.25 | 1.51E-03 |
| STM3245 | tdcA | DNA-binding transcriptional activator TdcA | -1.95 | 5.78 | 7.05E-06 |
| STM3247 | garK | glycerate kinase | -1.19 | 6.19 | 6.37E-03 |
| STM3250 | garD | galactarate dehydratase | -1.33 | 5.95 | 1.08E-03 |
| STM3254 | STM3254 | fructose-1-phosphate kinase | -3.22 | 4.84 | 6.68E-06 |
| STM3255 | STM3255 | PTS system fructose-specific transporter subunitIIB | -2.22 | 6.09 | 6.49E-06 |
| STM3256 | STM3256 | PTS system mannitol/fructose-specifictran sporter subunit IIA/phosphocarrier protein FPr | -2.00 | 5.54 | 3.11E-05 |
| STM3257 | STM3257 | tagatose 6-phosphate kinase 1 | -1.91 | 6.01 | 7.15E-06 |
| STM3258 | STM3258 | PTS system galactitol-specific transportersubunit IIA | -2.17 | 4.32 | 1.97E-05 |
| STM3259 | STM3259 | PTS system galactitol-specific transportersubunit IIB | -2.12 | 4.13 | 9.15E-04 |
| STM3260 | STM3260 | PTS system galactitol-specific transportersubunit IIC | -2.21 | 5.98 | 2.17E-06 |
| STM3261 | STM3261 | galactitol-1-phospha te dehydrogenase | -1.85 | 5.72 | 3.37E-05 |
| STM3277 | STM3277 | inner membrane protein | -2.42 | 5.05 | 1.10E-04 |
| STM3278 | STM3278 | hypothetical protein | -1.94 | 4.26 | 3.78E-03 |
| STM3329 | yhcC | FeS oxidoreductase | -1.30 | 5.86 | 2.37E-03 |
| STM3332 | yhcG | hypothetical protein | -2.13 | 5.73 | 5.01E-06 |
| STM3338 | nanT | sialic acid transporter | -1.26 | 6.14 | 2.42E-03 |
| STM3339 | nanA | N-acetylneuraminate lyase | -2.20 | 5.46 | 1.03E-04 |
| STM3343 | STM3343 | hypothetical protein | -1.37 | 5.73 | 1.13E-03 |
| STM3350 | STM3350 | inner membrane protein | -1.34 | 5.64 | 2.89E-03 |
| STM3351 | oadB | sodium ion pump oxaloacetate decarboxylasesubunit beta | -1.30 | 5.51 | 4.51E-02 |
| STM3352 | oadA | oxaloacetate decarboxylase | -1.90 | 4.72 | 3.56E-04 |
| STM3354 | STM3354 | L(+)-tartrate dehydratase subunit beta | -1.94 | 5.40 | 1.62E-03 |
| STM3355 | STM3355 | tartrate dehydratase subunit alpha | -2.21 | 5.58 | 3.07E-05 |
| STM3356 | STM3356 | cation transporter | -2.74 | 6.05 | 2.42E-05 |
| STM3364 | yhcP | p-hydroxybenzoic acid efflux subunit AaeB | -1.55 | 6.90 | 7.82E-04 |
| STM3365 | yhcQ | p-hydroxybenzoic acid efflux subunit AaeA | -1.08 | 5.57 | 2.23E-02 |
| STM3366 | yhcR | hypothetical protein | -1.66 | 3.12 | 2.79E-03 |
| STM3386 | yhdJ | methyltransferase | -1.67 | 5.81 | 2.13E-04 |
| STM3388 | STM3388 | signal transduction protein | -1.54 | 6.63 | 2.76E-04 |
| STM3389 | envR | DNA-binding transcriptional regulator EnvR | -2.33 | 4.59 | 7.15E-04 |
| STM3390 | acrE | acriflavine resistance protein E | -1.68 | 5.39 | 3.75E-04 |
| STM3391 | acrF | multidrug transport protein | -1.83 | 7.49 | 2.52E-03 |
| STM3442 | hopD | leader peptidase HopD | -2.30 | 4.59 | 7.15E-06 |
| STM3458 | yheR | glutathione-regulate d potassium-efflux systemancillary protein KefG | -1.31 | 4.94 | 1.48E-02 |
| STM3470 | fic | cell filamentation protein Fic | -1.06 | 4.79 | 1.44E-02 |
| STM3476 | nirC | nitrite transporter NirC | -1.66 | 5.45 | 1.28E-04 |
| STM3478 | bigA | surface-exposed virulence protein | -1.48 | 8.35 | 3.60E-02 |
| STM3488 | hofQ | outer membrane porin HofQ | -1.46 | 6.12 | 2.36E-04 |
| STM3489 | yrfA | inner membrane protein | -1.99 | 5.37 | 5.65E-04 |
| STM3490 | yrfB | inner membrane protein | -2.18 | 4.42 | 9.21E-05 |
| STM3491 | yrfC | inner membrane protein | -2.08 | 5.36 | 7.32E-05 |
| STM3492 | yrfD | hypothetical protein | -1.67 | 5.05 | 7.33E-04 |
| STM3499 | yhgE | inner membrane protein | -1.27 | 6.75 | 4.21E-03 |
| STM3512 | gntT | high-affinity gluconate permease | -1.17 | 5.95 | 8.16E-03 |
| STM3527 | STM3527 | hypothetical protein | -1.25 | 5.68 | 4.09E-03 |
| STM3528 | STM3528 | phosphate-binding protein | -1.04 | 7.08 | 3.17E-02 |
| STM3531 | STM3531 | dihydroxyacid dehydratase | -1.97 | 6.56 | 1.82E-05 |
| STM3532 | STM3532 | dihydrodipicolinate synthetase | -1.11 | 6.08 | 7.18E-03 |
| STM3541 | gntU | low affinity gluconate transporter | -1.50 | 5.97 | 2.06E-04 |
| STM3542 | gntK | gluconate kinase | -1.82 | 4.90 | 1.07E-04 |
| STM3547.Sc | STM3547.Sc | transcriptional regulator | -2.59 | 5.44 | 1.04E-05 |
| STM3548 | STM3548 | hypothetical protein | -2.37 | 5.12 | 2.15E-05 |
| STM3549 | STM3549 | inner membrane protein | -2.56 | 5.80 | 6.68E-06 |
| STM3550 | STM3550 | phosphotriesterase | -2.09 | 6.00 | 8.23E-06 |
| STM3551 | ggt | gamma-glutamyltransp eptidase | -1.84 | 6.48 | 3.07E-05 |
| STM3554 | ugpC | glycerol-3-phosphate transporter ATP-bindingsubunit | -1.14 | 6.48 | 8.90E-03 |
| STM3555 | ugpE | glycerol-3-phosphate transporter membraneprotein | -2.08 | 5.39 | 2.02E-05 |
| STM3556 | ugpA | glycerol-3-phosphate transporter permease | -2.31 | 5.00 | 1.64E-05 |
| STM3557 | ugpB | glycerol-3-phosphate transporter periplasmicbinding protein | -1.17 | 6.06 | 1.18E-02 |
| STM3558 | STM3558 | death-on-curing protein | -1.68 | 4.89 | 1.12E-03 |
| STM3560 | livF | leucine/isoleucine/v aline transporterATP-binding subunit | -1.21 | 5.00 | 6.29E-03 |
| STM3561 | livG | leucine/isoleucine/v aline transporterATP-binding subunit | -2.08 | 5.60 | 1.49E-06 |
| STM3562 | livM | leucine/isoleucine/v aline transporter permeasesubunit | -1.73 | 6.00 | 4.76E-05 |
| STM3563 | livH | branched-chain amino acid transporter permeaseLivH | -1.64 | 5.37 | 2.65E-04 |
| STM3564 | livK | high-affinity branched-chain amino acidtransporter | -1.33 | 5.77 | 8.75E-04 |
| STM3582 | yhhT | permease | -2.10 | 6.22 | 1.98E-05 |
| STM3599 | STM3599 | anaerobic C4-dicarboxylate transporter | -2.92 | 6.06 | 2.05E-06 |
| STM3600 | STM3600 | sugar kinase | -1.84 | 5.37 | 2.24E-04 |
| STM3601 | STM3601 | phosphosugar isomerase | -1.52 | 5.67 | 5.60E-04 |
| STM3605 | STM3605 | phage endolysin | -1.89 | 4.03 | 2.37E-04 |
| STM3606 | yhjB | transcriptional regulator | -2.12 | 4.63 | 1.74E-04 |
| STM3614 | dctA | C4-dicarboxylate transporter DctA | -1.37 | 6.39 | 6.33E-04 |
| STM3623 | yhjT | inner membrane protein | -1.74 | 3.93 | 2.08E-03 |
| STM3625 | yhjV | transporter | -2.54 | 6.83 | 3.54E-05 |
| STM3626 | dppF | dipeptide ABC transporter ATP-binding subunitDppF | -1.21 | 5.67 | 1.53E-02 |
| STM3627 | dppD | dipeptide ABC transporter ATP-binding subunitDppD | -1.57 | 5.75 | 1.28E-04 |
| STM3628 | dppC | dipeptide ABC transporter permease DppC | -1.60 | 5.50 | 1.31E-04 |
| STM3629 | dppB | dipeptide ABC transporter permease DppB | -1.57 | 5.90 | 3.73E-04 |
| STM3631 | STM3631 | xanthine permease | -1.86 | 6.16 | 7.45E-05 |
| STM3632 | STM3632 | hypothetical protein | -1.45 | 6.10 | 2.13E-03 |
| STM3635 | yhjW | phosphoethanolamine transferase | -1.02 | 6.73 | 1.88E-02 |
| STM3636 | lpfE | long polar fimbrial minor protein | -1.79 | 5.34 | 2.01E-04 |
| STM3637 | lpfD | long polar fimbrial protein | -1.45 | 6.27 | 4.07E-04 |
| STM3638 | lpfC | long polar fimbrial outer membrane usherprotein | -1.61 | 7.24 | 2.21E-03 |
| STM3639 | lpfB | long polar fimbrial chaperone precursor | -2.14 | 4.43 | 1.34E-04 |
| STM3640 | lpfA | long polar fimbrial protein A precursor | -2.50 | 5.32 | 1.22E-06 |
| STM3648 | yiaG | transcriptional regulator | -1.24 | 4.20 | 1.71E-02 |
| STM3653 | STM3653 | acetyltransferase | -1.69 | 4.92 | 2.67E-03 |
| STM3658 | yiaH | inner membrane protein | -2.22 | 5.81 | 1.41E-06 |
| STM3659 | yiaB | inner membrane protein | -2.28 | 4.74 | 8.07E-05 |
| STM3660 | xylB | xylulokinase | -1.16 | 6.10 | 3.90E-03 |
| STM3661 | xylA | xylose isomerase | -2.09 | 6.42 | 4.09E-06 |
| STM3662 | xylR | xylose operon regulatory protein | -1.50 | 6.06 | 1.16E-03 |
| STM3664 | malS | periplasmic alpha-amylase | -2.15 | 7.09 | 1.18E-04 |
| STM3668 | yiaK | 2,3-diketo-L-gulonat e reductase | -2.38 | 5.23 | 7.11E-06 |
| STM3669 | yiaL | hypothetical protein | -1.48 | 4.46 | 2.10E-02 |
| STM3670 | STM3670 | hypothetical protein | -2.14 | 5.69 | 1.82E-06 |
| STM3671 | yiaM | 2,3-diketo-L-gulonat e TRAP transporter smallpermease YiaM | -2.19 | 4.98 | 3.23E-04 |
| STM3672 | yiaN | hypothetical protein | -2.23 | 5.69 | 1.95E-04 |
| STM3673 | yiaO | periplasmic dicarboxylate-binding protein | -2.49 | 5.91 | 1.14E-03 |
| STM3674 | lyxK | L-xylulose kinase | -1.95 | 6.78 | 5.45E-03 |
| STM3675 | sgbH | 3-keto-L-gulonate-6- phosphate decarboxylase | -2.64 | 4.55 | 8.35E-05 |
| STM3676 | sgbU | L-xylulose 5-phosphate 3-epimerase | -2.53 | 4.87 | 2.06E-05 |
| STM3677 | sgbE | L-ribulose-5-phospha te 4-epimerase | -1.67 | 5.13 | 6.17E-04 |
| STM3678 | STM3678 | regulatory protein | -1.95 | 5.00 | 4.61E-05 |
| STM3679 | STM3679 | hypothetical protein | -1.69 | 6.26 | 8.16E-05 |
| STM3680 | aldB | aldehyde dehydrogenase B | -1.50 | 6.24 | 2.03E-04 |
| STM3681 | STM3681 | transcriptional regulator | -1.29 | 5.44 | 3.20E-03 |
| STM3690 | STM3690 | inner membrane lipoprotein | -2.56 | 5.63 | 2.47E-03 |
| STM3691 | STM3691 | trimeric autotransporter adhesin | -1.96 | 8.48 | 5.30E-03 |
| STM3692 | lldP | L-lactate permease | -2.30 | 6.10 | 1.46E-06 |
| STM3693 | lldR | DNA-binding transcriptional repressor LldR | -2.11 | 5.07 | 4.76E-05 |
| STM3694 | lldD | L-lactate dehydrogenase | -1.41 | 5.83 | 9.12E-04 |
| STM3697 | STM3697 | L-talarate/galactara te dehydratase | -2.14 | 5.89 | 1.99E-06 |
| STM3707 | yibD | glycosyl transferase family protein | -1.24 | 6.15 | 2.56E-03 |
| STM3729 | radC | DNA repair protein RadC | -1.55 | 5.18 | 3.75E-04 |
| STM3736 | STM3736 | transcriptional regulator | -1.18 | 6.32 | 2.83E-03 |
| STM3737 | STM3737 | Zn-dependent hydrolase | -1.89 | 5.85 | 2.59E-05 |
| STM3739 | ligB | NAD-dependent DNA ligase LigB | -1.27 | 7.32 | 1.72E-02 |
| STM3749 | yicI | alpha-xylosidase | -1.42 | 7.00 | 3.41E-03 |
| STM3750 | yicJ | transporter | -2.46 | 6.24 | 2.56E-06 |
| STM3756 | rmbA | hypothetical protein | -2.59 | 4.35 | 1.65E-04 |
| STM3757 | misL | autotransporter | -1.79 | 7.51 | 4.38E-03 |
| STM3758 | fidL | inner membrane protein | -1.45 | 4.50 | 4.30E-03 |
| STM3759.S | marT | transcriptional regulator | -1.79 | 5.02 | 9.70E-04 |
| STM3763 | mgtB | Mg2+ transporter | -1.95 | 7.64 | 4.20E-03 |
| STM3764 | mgtC | protein MgtC | -1.69 | 4.92 | 2.30E-03 |
| STM3767 | STM3767 | hypothetical protein | -1.03 | 5.71 | 3.69E-02 |
| STM3768 | STM3768 | selenocysteine synthase | -1.67 | 5.65 | 2.45E-04 |
| STM3769.S | STM3769.S | PTS system mannose-specific transporter subunitIID | -1.76 | 5.39 | 2.92E-04 |
| STM3770 | STM3770 | PTS system mannose-specific transporter subunitIIC | -2.22 | 5.45 | 4.41E-06 |
| STM3771 | STM3771 | PTS system mannose-specific transporter subunitIIB | -1.93 | 4.68 | 2.39E-04 |
| STM3772 | STM3772 | PTS system mannose-specific transporter subunitIIA | -1.77 | 3.70 | 1.21E-03 |
| STM3774 | STM3774 | inner membrane protein | -2.11 | 3.14 | 2.07E-04 |
| STM3776 | nepI | ribonucleoside transporter | -1.18 | 6.45 | 4.05E-03 |
| STM3779 | STM3779 | PTS system phosphocarrier protein HPr | -2.07 | 3.47 | 2.01E-03 |
| STM3781 | STM3781 | sugar kinase | -2.19 | 6.83 | 7.84E-05 |
| STM3782 | STM3782 | PTS system mannitol/fructose-specifictran sporter subunit IIC | -1.97 | 6.50 | 7.61E-05 |
| STM3783 | STM3783 | PTS system mannitol/fructose-specifictran sporter subunit IIB | -1.92 | 4.66 | 8.13E-04 |
| STM3784 | STM3784 | PTS system mannitol/fructose-specifictran sporter subunit IIA | -1.31 | 4.99 | 5.83E-03 |
| STM3786 | yicN | inner membrane protein | -1.82 | 5.77 | 4.16E-04 |
| STM3787 | uhpT | sugar phosphate antiporter | -1.42 | 6.40 | 4.18E-03 |
| STM3788 | uhpC | regulatory protein UhpC | -1.18 | 6.07 | 3.09E-02 |
| STM3791 | STM3791 | hypothetical protein | -1.41 | 5.91 | 7.08E-04 |
| STM3792 | STM3792 | L-fucose permease | -2.06 | 6.21 | 5.13E-06 |
| STM3793 | STM3793 | sugar kinase | -1.78 | 5.25 | 4.29E-04 |
| STM3795 | ilvN | acetolactate synthase 1 regulatory subunit | -1.05 | 4.48 | 1.86E-02 |
| STM3821 | torD | chaperone protein TorD | -1.81 | 5.25 | 4.17E-04 |
| STM3822 | torA | trimethylamine N-oxide reductase subunit | -1.65 | 7.05 | 1.26E-03 |
| STM3823 | torC | trimethylamine N-oxide reductase cytochromec-like subunit | -1.75 | 6.20 | 8.03E-05 |
| STM3827 | dgoT | D-galactonate transport protein | -2.26 | 6.33 | 1.34E-06 |
| STM3828 | dgoA | galactonate dehydratase | -2.21 | 6.04 | 2.68E-06 |
| STM3829 | dgoK | 2-oxo-3-deoxygalacto nate kinase | -1.86 | 5.64 | 1.16E-04 |
| STM3832 | STM3832 | permease | -2.76 | 5.89 | 3.61E-07 |
| STM3833 | STM3833 | mandelate racemase | -2.05 | 6.17 | 5.54E-06 |
| STM3847 | yidY | multidrug efflux system protein MdtL | -1.59 | 6.86 | 8.13E-04 |
| STM3858 | STM3858 | PTS system fructose-specific transporter subunitIIBC | -1.09 | 6.99 | 3.47E-02 |
| STM3863 | STM3863 | permease | -1.40 | 6.15 | 2.55E-03 |
| STM3881 | rbsD | D-ribose pyranase | -1.29 | 5.31 | 1.79E-03 |
| STM3882 | rbsA | D-ribose transporter ATP-binding protein | -1.48 | 6.72 | 8.43E-04 |
| STM3887 | yieO | tranport protein | -1.19 | 7.13 | 1.81E-02 |
| STM3899 | yifB | ATP-dependent protease | -1.06 | 6.70 | 1.21E-02 |
| STM3906 | STM3906 | hypothetical protein | -1.02 | 5.13 | 1.57E-02 |
| STM3911 | STM3911 | hypothetical protein | -2.06 | 4.21 | 3.49E-04 |
| STM3912 | rep | ATP-dependent DNA helicase Rep | -1.03 | 7.25 | 4.45E-02 |
| STM3940 | STM3940 | inner membrane protein | -1.53 | 4.62 | 4.85E-03 |
| STM3941 | STM3941 | inner membrane protein | -2.52 | 4.44 | 2.68E-06 |
| STM3942 | STM3942 | hypothetical protein | -1.69 | 4.59 | 3.49E-04 |
| STM3944 | STM3944 | inner membrane protein | -1.69 | 4.46 | 5.77E-04 |
| STM3953 | yigF | inner membrane protein | -2.05 | 4.51 | 3.57E-03 |
| STM3954 | yigG | hypothetical protein | -1.79 | 4.30 | 1.40E-02 |
| STM3956 | yigI | hypothetical protein | -1.87 | 4.56 | 1.70E-04 |
| STM3963 | yigM | transporter | -1.30 | 5.82 | 3.51E-03 |
| STM3964 | metR | metE/metH regulator | -1.12 | 5.30 | 1.13E-02 |
| STM3965 | metE | 5-methyltetrahydropt eroyltriglutamate/homocysteine S-methyltransferase | -1.50 | 7.42 | 9.57E-03 |
| STM3966 | STM3966 | arylsulfatase regulator | -1.42 | 5.99 | 7.36E-04 |
| STM3980 | STM3980 | outer membrane protein | -1.66 | 4.55 | 1.84E-03 |
| STM3981 | STM3981 | hypothetical protein | -1.52 | 6.42 | 2.98E-04 |
| STM3982 | fadA | 3-ketoacyl-CoA thiolase | -2.01 | 6.27 | 1.59E-05 |
| STM3983 | fadB | multifunctional fatty acid oxidation complexsubunit alpha | -2.40 | 6.58 | 6.94E-06 |
| STM3998 | yihG | acyltransferase | -1.01 | 6.39 | 2.47E-02 |
| STM4010 | STM4010 | hydrolase | -1.24 | 5.52 | 2.68E-03 |
| STM4011 | STM4011 | inner membrane protein | -2.34 | 5.38 | 2.73E-06 |
| STM4012 | STM4012 | coproporphyrinogen III oxidase | -2.29 | 5.45 | 7.77E-06 |
| STM4013.S | STM4013.S | membrane-associated metal-dependent hydrolase | -2.24 | 5.60 | 2.37E-05 |
| STM4014 | STM4014 | hypothetical protein | -2.61 | 4.88 | 4.79E-07 |
| STM4015 | STM4015 | hypothetical protein | -1.76 | 5.90 | 3.11E-04 |
| STM4016 | ompL | outer membrane porin L | -2.38 | 4.84 | 3.23E-04 |
| STM4017 | yihO | GPH family transport protein | -2.06 | 6.69 | 2.01E-03 |
| STM4018 | yihP | GPH family transport protein | -2.00 | 6.35 | 7.82E-06 |
| STM4019 | yihQ | alpha-glucosidase | -1.82 | 6.71 | 1.18E-04 |
| STM4020.S | yihR | aldose-1-epimerase | -1.47 | 5.70 | 6.13E-04 |
| STM4021 | yihS | isomerase | -1.95 | 6.16 | 1.46E-05 |
| STM4022 | yihT | aldolase | -1.78 | 4.94 | 2.48E-04 |
| STM4023 | yihU | oxidoreductase | -2.22 | 5.48 | 7.85E-05 |
| STM4024.S | yihV | sugar kinase | -1.93 | 6.24 | 6.68E-06 |
| STM4039 | STM4039 | inner membrane lipoprotein | -1.62 | 5.72 | 1.38E-04 |
| STM4040 | yiiG | hypothetical protein | -3.07 | 5.61 | 1.23E-05 |
| STM4041 | STM4041 | inner membrane protein | -1.88 | 3.17 | 1.22E-03 |
| STM4045 | rhaD | rhamnulose-1-phospha te aldolase | -1.62 | 5.84 | 2.10E-03 |
| STM4046 | rhaA | L-rhamnose isomerase | -2.28 | 5.40 | 5.13E-06 |
| STM4047 | rhaB | rhamnulokinase | -2.45 | 6.04 | 1.49E-06 |
| STM4048 | rhaS | transcriptional activator RhaS | -1.97 | 5.67 | 2.54E-05 |
| STM4049 | rhaR | transcriptional activator RhaR | -1.73 | 5.45 | 1.01E-04 |
| STM4050 | rhaT | rhamnose-proton symporter | -2.06 | 6.17 | 2.50E-05 |
| STM4051 | STM4051 | outer membrane protein | -2.19 | 5.04 | 3.12E-04 |
| STM4052 | STM4052 | C4-dicarboxylate transport system | -2.10 | 6.25 | 2.27E-06 |
| STM4053 | STM4053 | C4-dicarboxylate transport system | -2.46 | 4.35 | 2.50E-05 |
| STM4054 | STM4054 | periplasmic dicarboxylate-binding protein | -2.45 | 5.68 | 1.47E-05 |
| STM4065 | STM4065 | Na+/galactoside symporter | -2.48 | 6.66 | 1.49E-04 |
| STM4066 | STM4066 | aminoimidazole riboside kinase | -2.14 | 6.34 | 7.87E-05 |
| STM4070 | STM4070 | hypothetical protein | -1.21 | 3.00 | 1.74E-02 |
| STM4071 | STM4071 | mannose-6-phosphate isomerase | -1.06 | 3.71 | 4.64E-02 |
| STM4072 | ydeV | autoinducer-2 (AI-2) kinase LsrK | -1.67 | 6.24 | 1.13E-04 |
| STM4073 | STM4073 | transcriptional repressor LysR | -1.29 | 6.00 | 5.94E-03 |
| STM4074 | STM4074 | autoinducer 2 import sysem ATP-binding proteinLsrA | -1.69 | 6.04 | 5.45E-04 |
| STM4075 | STM4075 | autoinducer 2 import system permease LsrC | -2.15 | 6.11 | 2.89E-04 |
| STM4076 | STM4076 | autoinducer 2 import system permease LsrD | -2.22 | 5.90 | 4.78E-06 |
| STM4077 | STM4077 | autoinducer 2-binding protein LsrB | -1.85 | 5.91 | 2.02E-05 |
| STM4079.S | STM4079.S | autoinducer-2 (AI-2) modifying protein LsrG | -1.65 | 4.70 | 2.29E-03 |
| STM4087 | glpF | glycerol diffusion protein | -1.56 | 6.11 | 4.54E-04 |
| STM4098 | STM4098 | arylsulfate sulfotransferase | -2.02 | 6.26 | 5.54E-06 |
| STM4102 | STM4102 | mechanosensitive channel protein | -1.60 | 5.70 | 4.90E-04 |
| STM4103 | STM4103 | hypothetical protein | -1.39 | 5.89 | 2.60E-03 |
| STM4104 | STM4104 | 5'-nucleotidase | -1.73 | 6.74 | 2.08E-04 |
| STM4110 | ptsA | PEP-protein phosphotransferase | -1.04 | 7.10 | 3.87E-02 |
| STM4112 | frwC | PTS system fructose-like transporter subunitIIC | -1.73 | 5.79 | 7.45E-05 |
| STM4113 | frwB | PTS system fructose-like transporter subunitIIB | -1.39 | 5.25 | 1.69E-03 |
| STM4141 | STM4141 | hypothetical protein | -2.18 | 3.91 | 1.90E-04 |
| STM4156 | STM4156 | hypothetical protein | -1.62 | 3.60 | 6.77E-03 |
| STM4157 | STM4157 | hypothetical protein | -1.48 | 6.14 | 5.47E-03 |
| STM4159 | thiH | thiamine biosynthesis protein ThiH | -1.83 | 6.36 | 9.39E-05 |
| STM4160 | thiG | thiazole synthase | -1.43 | 5.19 | 2.05E-02 |
| STM4161 | STM4161 | sulfur carrier protein ThiS | -1.56 | 2.34 | 1.01E-02 |
| STM4162 | thiF | thiamine biosynthesis protein ThiF | -2.11 | 4.78 | 3.13E-05 |
| STM4163 | thiE | thiamine-phosphate pyrophosphorylase | -1.81 | 4.41 | 1.33E-04 |
| STM4164 | thiC | hydroxymethylpyrimid ine phosphate synthase ThiC | -1.42 | 6.87 | 1.53E-03 |
| STM4172 | zraP | zinc resistance protein | -1.06 | 5.04 | 1.66E-02 |
| STM4182 | metA | homoserine O-succinyltransferase | -1.50 | 6.40 | 2.42E-04 |
| STM4183 | aceB | malate synthase | -1.99 | 6.37 | 7.84E-05 |
| STM4184 | aceA | isocitrate lyase | -1.85 | 5.83 | 1.64E-05 |
| STM4185 | aceK | bifunctional isocitrate dehydrogenasekinase/phosphatas e protein | -1.55 | 6.67 | 4.93E-04 |
| STM4196 | STM4196 | hypothetical protein | -1.03 | 5.64 | 1.57E-02 |
| STM4198 | STM4198 | hypothetical protein | -1.18 | 4.62 | 1.56E-02 |
| STM4199 | STM4199 | hypothetical protein | -2.19 | 5.70 | 7.32E-06 |
| STM4200 | STM4200 | phage tail fiber protein H | -1.57 | 7.06 | 7.85E-03 |
| STM4201 | STM4201 | phage tail protein | -2.27 | 4.66 | 2.27E-04 |
| STM4202 | STM4202 | phage baseplate protein | -1.98 | 4.83 | 4.38E-05 |
| STM4203 | STM4203 | phage baseplate protein | -2.47 | 4.07 | 2.01E-06 |
| STM4204 | STM4204 | inner membrane protein | -1.53 | 6.66 | 1.07E-03 |
| STM4205 | STM4205 | phage glycosyltransferase | -1.59 | 5.78 | 4.83E-04 |
| STM4207 | STM4207 | phage baseplate component | -1.64 | 5.44 | 3.78E-04 |
| STM4208 | STM4208 | hypothetical protein | -2.05 | 5.44 | 7.46E-06 |
| STM4209 | STM4209 | inner membrane protein | -2.13 | 3.24 | 3.78E-04 |
| STM4210 | STM4210 | methyl-accepting chemotaxis protein | -2.21 | 6.11 | 7.92E-06 |
| STM4211 | STM4211 | phage tail protein | -2.01 | 6.23 | 1.40E-05 |
| STM4212 | STM4212 | phage tail core protein | -1.91 | 4.86 | 2.99E-04 |
| STM4213 | STM4213 | phage tail sheath protein | -1.85 | 6.07 | 1.97E-05 |
| STM4214 | STM4214 | hypothetical protein | -2.06 | 2.54 | 6.59E-04 |
| STM4215 | STM4215 | hypothetical protein | -2.12 | 3.85 | 9.39E-05 |
| STM4216 | STM4216 | inner membrane protein | -2.13 | 3.84 | 2.29E-03 |
| STM4217 | STM4217 | soluble lytic murein transglycosylase | -1.55 | 4.27 | 7.75E-03 |
| STM4218 | STM4218 | inner membrane protein | -1.70 | 3.36 | 2.72E-03 |
| STM4219.S | STM4219.S | hypothetical protein | -1.03 | 4.67 | 2.70E-02 |
| STM4223 | yjbF | outer membrane lipoprotein | -2.65 | 4.55 | 1.44E-06 |
| STM4224 | yjbG | hypothetical protein | -1.79 | 4.96 | 3.80E-04 |
| STM4225 | yjbH | outer membrane lipoprotein | -1.55 | 7.34 | 5.07E-03 |
| STM4226 | yjbA | phosphate-starvation -inducible protein PsiE | -1.35 | 5.01 | 3.90E-03 |
| STM4227 | malG | maltose transporter permease | -1.02 | 5.55 | 2.44E-02 |
| STM4228 | malF | maltose transporter membrane protein | -1.75 | 6.36 | 3.05E-05 |
| STM4230 | malK | maltose/maltodextrin transporter ATP-bindingprotein | -1.59 | 6.29 | 4.29E-03 |
| STM4231 | lamB | maltoporin | -1.30 | 6.02 | 1.42E-03 |
| STM4257 | STM4257 | hypothetical protein | -3.10 | 5.92 | 3.59E-10 |
| STM4258 | STM4258 | methyl-accepting chemotaxis protein | -2.67 | 6.64 | 5.51E-07 |
| STM4259 | STM4259 | ABC exporter outer membrane protein | -2.27 | 6.57 | 1.34E-05 |
| STM4260 | STM4260 | cation efflux pump | -2.41 | 6.03 | 1.44E-06 |
| STM4262 | STM4262 | bacteriocin/lantibio tic ABC transporter | -2.14 | 6.43 | 1.31E-04 |
| STM4264 | yjcC | diguanylate cyclase/phosphodiesterase | -1.07 | 6.85 | 3.34E-02 |
| STM4273 | actP | acetate permease | -1.40 | 6.78 | 8.38E-03 |
| STM4274 | yjcH | inner membrane protein | -2.80 | 3.88 | 2.60E-06 |
| STM4275 | acs | acetyl-CoA synthetase | -1.97 | 6.39 | 7.64E-06 |
| STM4277 | nrfA | cytochrome c552 | -1.27 | 6.35 | 4.17E-03 |
| STM4280 | nrfD | formate-dependent nitrate reductase | -1.63 | 5.80 | 3.55E-04 |
| STM4281 | nrfE | formate-dependent nitrite reductase | -1.68 | 7.10 | 1.91E-03 |
| STM4297 | melR | DNA-binding transcriptional regulator MelR | -1.59 | 5.84 | 2.24E-04 |
| STM4298 | melA | alpha-galactosidase | -2.42 | 6.30 | 2.97E-06 |
| STM4299 | melB | melibiose:sodium symporter | -1.70 | 6.58 | 1.10E-04 |
| STM4305.S | STM4305.S | anaerobic dimethylsulfoxide reductase subunit A | -1.02 | 6.92 | 2.75E-02 |
| STM4306 | STM4306 | anaerobic dimethylsulfoxide reductase subunit B | -1.32 | 5.99 | 2.47E-03 |
| STM4309 | STM4309 | hypothetical protein | -2.15 | 6.27 | 3.79E-06 |
| STM4310 | STM4310 | inner membrane protein | -1.08 | 6.00 | 2.27E-02 |
| STM4312 | STM4312 | hypothetical protein | -1.64 | 4.53 | 6.89E-04 |
| STM4313 | STM4313 | hypothetical protein | -1.09 | 4.13 | 1.91E-02 |
| STM4314 | STM4314 | regulatory protein | -1.77 | 3.99 | 5.45E-03 |
| STM4315 | STM4315 | DNA-binding protein | -1.21 | 6.20 | 2.84E-02 |
| STM4345 | yjeM | amino-acid transport protein | -1.81 | 6.42 | 1.22E-04 |
| STM4346 | yjeO | inner membrane protein | -1.74 | 5.43 | 3.13E-05 |
| STM4370 | yjfI | hypothetical protein | -2.59 | 3.59 | 1.54E-05 |
| STM4371 | yjfJ | phage shock protein A | -1.96 | 4.67 | 5.65E-04 |
| STM4372 | STM4372 | potassium channels | -2.40 | 5.97 | 6.29E-06 |
| STM4373 | yjfK | hypothetical protein | -2.67 | 4.98 | 7.20E-07 |
| STM4374 | yjfL | inner membrane protein | -1.82 | 3.99 | 6.57E-04 |
| STM4375 | yjfM | inner membrane protein | -1.81 | 5.00 | 3.90E-05 |
| STM4376 | yjfC | glutathionylspermidi ne synthase | -1.77 | 5.78 | 2.64E-05 |
| STM4377 | aidB | isovaleryl CoA dehydrogenase | -1.17 | 6.69 | 7.19E-03 |
| STM4382 | yjfR | L-ascorbate 6-phosphate lactonase | -1.85 | 5.67 | 1.85E-04 |
| STM4384 | sgaB | PTS system L-ascorbate-specific transportersubunit IIB | -1.84 | 3.82 | 3.58E-04 |
| STM4385 | ptxA | PTS system L-ascorbate-specific transportersubunit IIA | -1.95 | 4.57 | 1.07E-04 |
| STM4386 | ulaD | 3-keto-L-gulonate-6- phosphate decarboxylase | -1.17 | 5.09 | 6.83E-03 |
| STM4388 | sgaE | L-ribulose-5-phospha te 4-epimerase | -1.59 | 5.60 | 1.75E-03 |
| STM4390 | STM4390 | hypothetical protein | -2.15 | 3.03 | 4.25E-04 |
| STM4395 | yifZ | permease | -1.69 | 5.61 | 8.40E-05 |
| STM4399 | ytfE | iron-sulfur cluster repair di-iron protein | -1.47 | 4.79 | 2.82E-03 |
| STM4400 | ytfF | cationic amino acid transporter | -1.83 | 5.20 | 5.67E-05 |
| STM4401 | ytfG | reductase | -1.41 | 5.23 | 3.52E-03 |
| STM4413 | STM4413 | metallo-dependent hydrolase | -2.31 | 6.24 | 6.99E-06 |
| STM4418 | STM4418 | sugar transporter | -1.93 | 6.52 | 1.33E-05 |
| STM4419 | STM4419 | sugar transporter | -2.14 | 6.94 | 4.38E-05 |
| STM4420 | STM4420 | inner membrane protein | -1.02 | 6.30 | 1.03E-02 |
| STM4423 | STM4423 | DNA-binding protein | -1.69 | 5.90 | 9.76E-04 |
| STM4424.S | STM4424.S | endonuclease | -2.69 | 5.10 | 5.27E-04 |
| STM4425 | STM4425 | dehydrogenase | -2.32 | 6.37 | 1.47E-06 |
| STM4426 | srfJ | lysosomal glucosyl ceramidase-like protein | -1.81 | 5.86 | 4.51E-05 |
| STM4427 | STM4427 | endonuclease | -1.12 | 6.38 | 6.03E-03 |
| STM4428 | STM4428 | major facilitator superfamily transporter | -1.07 | 5.43 | 1.64E-02 |
| STM4433 | STM4433 | myo-inositol 2-dehydrogenase | -2.13 | 6.13 | 6.68E-06 |
| STM4434 | STM4434 | permease | -1.95 | 6.42 | 2.83E-03 |
| STM4435 | STM4435 | hypothetical protein | -2.41 | 5.06 | 6.23E-06 |
| STM4436 | STM4436 | endonuclease | -1.95 | 6.11 | 1.50E-04 |
| STM4440 | STM4440 | hypothetical protein | -2.52 | 3.45 | 7.16E-06 |
| STM4441 | STM4441 | hypothetical protein | -1.94 | 3.59 | 5.77E-04 |
| STM4442 | STM4442 | hypothetical protein | -1.97 | 3.64 | 2.85E-04 |
| STM4443 | STM4443 | inner membrane protein | -1.85 | 5.05 | 1.64E-04 |
| STM4444 | STM4444 | inner membrane protein | -2.00 | 5.04 | 3.81E-04 |
| STM4445 | STM4445 | dihydroorotase | -1.81 | 5.85 | 1.76E-04 |
| STM4446 | STM4446 | selenocysteine synthase | -1.90 | 6.03 | 1.81E-04 |
| STM4447 | STM4447 | hypothetical protein | -1.38 | 4.85 | 3.21E-03 |
| STM4448 | STM4448 | PTS system mannitol/fructose-specifictran sporter subunit IIA | -1.10 | 7.24 | 3.24E-02 |
| STM4453 | treC | trehalose-6-phosphat e hydrolase | -1.69 | 5.99 | 8.58E-05 |
| STM4456 | mgtA | magnesium-transporti ng ATPase MgtA | -1.42 | 7.30 | 7.56E-03 |
| STM4463 | STM4463 | arginine repressor | -1.76 | 4.13 | 1.41E-03 |
| STM4464 | STM4464 | arginine repressor | -1.32 | 6.23 | 1.87E-03 |
| STM4465 | STM4465 | ornithine carbamoyltransferase | -2.25 | 5.06 | 5.67E-05 |
| STM4466 | STM4466 | carbamate kinase | -1.85 | 5.06 | 1.11E-04 |
| STM4467 | STM4467 | arginine deiminase | -2.73 | 5.95 | 3.94E-06 |
| STM4469 | argI | ornithine carbamoyltransferase subunit I | -1.76 | 5.66 | 3.42E-05 |
| STM4472 | ytgA | inner membrane protein | -1.90 | 4.65 | 9.41E-04 |
| STM4474 | yjgN | inner membrane protein | -1.99 | 6.61 | 9.85E-06 |
| STM4481 | idnR | L-idonate regulator | -1.11 | 5.92 | 9.70E-03 |
| STM4482 | idnT | L-idonate transport protein | -1.68 | 5.57 | 1.64E-04 |
| STM4483 | idnO | gluconate 5-dehydrogenase | -2.04 | 4.79 | 5.88E-05 |
| STM4484 | idnD | L-idonate 5-dehydrogenase | -1.88 | 5.38 | 2.50E-05 |
| STM4485 | idnK | D-gluconate kinase | -1.66 | 4.48 | 7.35E-04 |
| STM4486 | yjgB | alcohol dehydrogenase | -1.03 | 5.51 | 1.14E-02 |
| STM4488 | STM4488 | integrase | -1.30 | 3.18 | 1.70E-02 |
| STM4502 | STM4502 | hypothetical protein | -2.25 | 5.28 | 2.02E-05 |
| STM4503 | STM4503 | inner membrane protein | -1.30 | 5.83 | 3.28E-03 |
| STM4504 | STM4504 | hypothetical protein | -2.23 | 5.23 | 1.64E-05 |
| STM4505 | STM4505 | hypothetical protein | -1.15 | 5.51 | 4.54E-03 |
| STM4516 | yjiN | inner membrane protein | -1.11 | 5.67 | 7.13E-03 |
| STM4517 | yjiO | transporter | -1.09 | 6.46 | 2.09E-02 |
| STM4519 | STM4519 | NAD-dependent aldehyde dehydrogenase | -1.21 | 5.97 | 2.65E-03 |
| STM4521 | yjiS | hypothetical protein | -1.51 | 2.77 | 9.16E-03 |
| STM4522 | STM4522 | inner membrane protein | -2.72 | 5.51 | 1.07E-04 |
| STM4523 | yjiW | endoribonuclease SymE | -1.33 | 3.43 | 1.32E-02 |
| STM4534 | STM4534 | transcriptional regulator | -1.45 | 7.31 | 9.63E-03 |
| STM4535 | STM4535 | PTS system mannose-specific transporter subunitIIA | -2.66 | 4.30 | 3.29E-05 |
| STM4536 | STM4536 | PTS system mannose-specific transporter subunitIIB | -2.12 | 4.51 | 6.33E-03 |
| STM4537 | STM4537 | PTS system mannose-specific transporter subunitIIC | -2.56 | 4.95 | 2.42E-04 |
| STM4538 | STM4538 | PTS system mannose-specific transporter subunitIID | -2.27 | 5.15 | 3.40E-06 |
| STM4539 | STM4539 | glucosamine-fructose -6-phosphateaminotransferase | -2.16 | 5.33 | 2.98E-04 |
| STM4540.S | STM4540.S | glucosamine-fructose -6-phosphateaminotransferase | -1.30 | 5.72 | 1.21E-03 |
| STM4547 | yjjQ | transcriptional regulator | -2.80 | 4.94 | 3.09E-05 |
| STM4561 | osmY | hypothetical protein | -1.07 | 5.22 | 1.23E-02 |
| STM4572 | stjB | fimbrial usher protein | -1.44 | 6.89 | 2.01E-03 |
| STM4573 | stjC | periplasmic chaperone protein | -2.37 | 5.03 | 3.18E-06 |
| STM4575 | STM4575 | outer membrane protein | -1.52 | 5.91 | 7.19E-04 |
| STM4590 | creD | hypothetical protein | -1.14 | 6.20 | 4.37E-03 |
| STM4591 | sthE | major fimbrial subunit | -1.74 | 6.03 | 2.67E-05 |
| STM4593 | sthB | fimbrial usher protein | -2.25 | 6.75 | 6.68E-06 |
| STM4594 | sthA | fimbrial chaperone | -1.82 | 5.15 | 5.27E-03 |
| STM4595 | STM4595 | fimbrial chaperone | -1.96 | 4.28 | 2.48E-04 |
| STM4596 | STM4596 | inner membrane protein | -1.09 | 5.26 | 1.13E-02 |
